# Shared Plant-human Biology: Herbicide Effects and New Biomarkers Perspectives

**DOI:** 10.1007/s40572-026-00523-z

**Published:** 2026-04-02

**Authors:** Aline de Souza Espindola, Christine Gibson Parks, Josino Costa Moreira

**Affiliations:** 1 Occupational and Environmental Health Branch, Public Health Institute, Universidade Federal do, Rio de Janeiro, Brazil; 2 National Institute of Environmental Health Sciences US, National Institutes of Health, Durham, NC, United States of America

**Keywords:** Herbicides; humans, Plants, Biomarkers of effect, Immune regulation

## Abstract

**Purpose of Review:**

Herbicide exposure has been associated with acute poisoning and several chronic diseases. Humans and plants share biological processes targeted by herbicides. Investigating these shared pathways can uncover crucial insights into the underlying mechanisms of herbicides in humans, identify potential biomarkers of herbicide-induced effects, and advance our understanding of related biological processes.

**Recent Findings:**

The tubulin and key enzymes of metabolic pathways, such as hydroxyphenylpyruvate dioxygenase (HPPD), acetyl-CoA carboxylase (ACC), glutamine synthetase (GS), and protoporphyrinogen oxidase (PPO), have been identified as molecular targets of herbicides in humans. These molecules exhibit relative selectivity for herbicide exposure and can be quantified using well-established techniques, thereby representing potential effect biomarkers. Some of these enzymes play crucial roles in regulating all aspects of the immune response. Furthermore, these shared enzymes, as well as other enzymes involved in plant-specific metabolic pathways that are not present in humans, are found in bacteria and fungi of the gut microbiota, therefore also representing potential targets of herbicides.

**Summary:**

This study highlights molecular targets of herbicides in humans, which may serve as early indicators of biological changes preceding adverse effects or disease. Some of these enzymes are involved in key steps of the regulation of immune responses, which may thereby be disrupted by herbicide exposure. These targets, along with other plant-specific molecules, are also present in gut microbiota microorganisms, where herbicide exposure may disrupt their function and provide a mechanistic link to intestinal dysbiosis, a condition increasingly associated with chronic diseases.

**Supplementary Information:**

The online version contains supplementary material available at 10.1007/s40572-026-00523-z.

## Introduction

Herbicides control or eliminate undesirable plants in agricultural fields, gardens, and others. Their use has risen globally, mainly due to the necessity of increasing food production and supporting the adoption of genetic modifications, leading to the development of herbicide-resistant weeds [1–3]. Indeed, in 2021, global consumption of various types of pesticides was estimated at 3,540,000 million tons, with herbicides accounting for nearly 50% of this total[4], though use varies significantly from region to region based on agricultural practices, crop types, economic factors, and government regulations [5–7]. 

Exposure to herbicides, particularly among agricultural workers, can lead to both acute and chronic health problems. Acute poisoning may result from accidental or intentional exposure, manifesting in a myriad of symptoms [8–14] that can vary depending on the type of herbicide, its concentration, and the duration of exposure [15]. In addition to acute effects, long-term occupational and environmental exposure to various herbicides has been associated with an increased risk of certain cancers, including non-Hodgkin lymphoma and colorectal cancer[16–18], Parkinson’s disease[19], chronic kidney disease[20–22], rheumatoid arthritis, and systemic lupus erythematosus (SLE) [23, 24]. 

Plants and humans have similar metabolic processes, such as cellular respiration and synthesizing proteins, nucleic acids, and lipids [25–29]. Exploring these shared biological processes can provide insights into the molecular targets of herbicide exposure in humans, identify potential biomarkers of herbicide-induced effects, and shed light on the underlying mechanisms of herbicide toxicity. We conducted a comprehensive review of the mechanisms of action of herbicides in plants and of metabolic processes shared between plants and humans, aiming to propose molecular alterations common between plants and humans as potential early biomarkers of herbicide exposure in humans.

## Discussion

### Herbicide Classes and their Action Mechanisms in Plants: a Brief Overview

Herbicides are used to control or eliminate undesirable plants in diverse settings, ranging from agricultural fields, along roadways, landscaping, to home gardens. Based on their chemical structure, herbicides are classified into several chemical classes (Table 1). The main herbicide mechanisms involve interferences with specific physiological or biochemical processes within plants, disrupting their growth, development, or metabolism.

**Table 1:** Herbicide chemical classes and examples

Chemical class^*^	Examples	Primary molecular target in plants	Molecular target in humans
Phenoxyacetic acids	2,4-dichloro phenoxy acetic acid, 2,4,5-trichloro phenoxy acetic acid, mecoprop	Auxin signaling pathway	No
Pyridine carboxylic acids	Picloram, clopyralid, aminopyralid	Auxin signaling pathway	No
Bipyridylium compounds	Paraquat, diquat	Photosystem I (redox cycling)	No
Imidazoline	Imazapyr, imazaquin, imazamox, imazethapyr	Acetolactate synthase (ALS)	No
Triazines	Atrazine, simazine, metribuzin	Photosystem II	No
Glycine derivative	Glyphosate	5-enolpyruvylshikimate-3-phosphate synthase (EPSPS)	No
Isoxazoles	Isoxaflutole, pyrasulfotole	4-hydroxyphenylpyruvate dioxygenase (HPPD)	HPPD
Triketones	Bicyclopyrone, sulcotrione, tembotrione	HPPD	HPPD
Dinitroanilines	Trifluralin, pendimethalin	Tubulin dimers	Tubulin dimers
Aryloxy-phenoxy-propionates	Diclofop-methyl, quizalofop-p-ethyl, clodinafop-propargyl	Acetyl-CoA carboxylase (ACC)	ACC
Chloroacetamide	Acetochlor, metolachlor	Geranylgeranyl pyrophosphate (GGPP) cyclization enzymes	No
Diphenylethers	Aclonifen, nitrofen	Protoporphyrinogen oxidase (PPO)	PPO
Oxazolidinones	Pentoxazone	PPO	PPO
Pyrimidinediones	Benzfendizone, butafenacil, saflufenacil	PPO	PPO
N-phenylethanamides	Cinidon, flumiclorac, flumioxazin	PPO	PPO
Phosphinic acids	Glufosinate, bilanafos	Glutamine synthetase (GS)	GS

* Adapted by Kraehmer et al. 2014[30]

#### Phenoxyacetic and Pyridine Carboxylic Acids

These herbicide classes are auxin receptor agonists that affect normal cell elongation, root development, and plant growth [31–33]. Additionally, phenoxyacetic herbicides can disrupt cell membranes, affecting nutrient uptake, ion transport, and signal transduction [34, 35]. They also interfere with chlorophyll synthesis and photosystem function, reducing the plant’s ability to produce energy from sunlight and reducing growth and death [36]. Furthermore, both herbicide classes induce oxidative stress, causing damage to proteins, lipids, and DNA, which leads to tissue necrosis and cell death [37, 38]. 

#### Bipyridylium Compounds

The main mechanisms of bipyridylium herbicides involve generating reactive oxygen species (ROS) [39]. These substances are not metabolized and undergo repeated redox cycling processes, producing ROS inside cells, leading to oxidative stress and damaging cellular components. They also act targets photosystem I (PSI), specifically, ferredoxin, preventing the reduction of nicotinamide adenine dinucleotide phosphate (NADP^+^) to NADPH and disrupting the synthesis of ATP, an energy source in plant cells [40]. The combined effects of oxidative stress and interference with metabolic processes that use ATP led to plant death.

#### Imidazolinones

These herbicides inhibit the enzyme acetolactate synthase (ALS), a key enzyme in the biosynthesis of branched-chain amino acids valine, leucine, and isoleucine, and its inhibition leads to the disruption of protein synthesis and plant death [41]. Other toxic mechanisms for plants include impairing mitosis and cytokinesis, leading to abnormal growth patterns and cell division defects [42]. Imidazolinone herbicides can also affect chlorophyll synthesis and thylakoid membrane structure, reducing the plant’s ability to absorb water and nutrients from the soil, leading to water stress and nutrient deficiency [43–45]. 

#### Triazines

Triazine herbicides interfere with photosynthesis by inhibiting the enzyme photosystem II (PSII) and the photosynthetic electron transport chain, reducing ATP and NADPH synthesis [46, 47]. Disruption of the electron transport chain leads to an imbalance of energy and electron flow in the chloroplasts with ROS production and oxidative stress [48, 49]. Taken together, the toxic effects of triazine herbicides lead to plant tissue necrosis and death.

#### Glycine Derivative

Compounds derived from glycine deplete glutathione, an intracellular antioxidant that protects cells from oxidative stress [50]. It also can disrupt the antioxidant enzyme derivative of glutathione and inhibit 5-enolpyruvylshikimate-3-phosphate synthase (EPSPS), a key enzyme in the shikimate pathway [51]. EPSPS is responsible for converting phosphoenolpyruvate and shikimate-3-phosphate into 5-enolpyruvylshikimate-3-phosphate. This step is essential for synthesizing aromatic amino acids, such as tryptophan. Shikimate-3-phosphate accumulation disrupts the production of aromatic amino acids. It also induces oxidative stress by increasing ROS production and impairing antioxidant defense mechanisms [52, 53]. For example, glyphosate has been shown to disrupt mitochondrial function, leading to increased ROS generation and oxidative damage to cellular components. These effects lead to oxidative stress with lipid peroxidation, protein oxidation, and DNA damage. The consequences of glyphosate-induced oxidative stress in plants include reduced photosynthetic efficiency, altered growth patterns, and compromised stress tolerance [54]. 

#### Isoxazoles and Triketones

4-hydroxyphenylpyruvate dioxygenase (HPPD) impedes the conversion of 4-hydroxyphenylpyruvate, a tyrosine degradation product, to homogentisate, an important stage in the shikimate pathway, hindering the biosynthesis of tocopherol and plastoquinone [55, 56]. Moreover, HPPD inhibitors also interfere with the synthesis of carotenoid pigments, which play a crucial role in protecting chlorophyll and facilitating photosynthesis [57, 58]. Consequently, HPPD inhibitors can lead to uncoupling of photosynthesis, reduction of photoprotection provided by carotenoids with impaired plant growth, decreased vigor, and death.

#### Dinitroanilines

Dinitroanilines interfere with the polymerization of tubulin dimers into microtubules, a protein essential in cell division, elongation, and overall plant growth [59]. Inhibition of microtubule formation disrupts the normal process of cell division, leading to abnormalities in cell shape, cell wall synthesis, and overall plant growth. It harms root development because growing root tips have a high cell division and elongation rate [59]. Inhibition of microtubules in root cells leads to stunted root growth and abnormal root development.

#### Aryloxy-phenoxy-propionates

Aryloxy-phenoxy-propionates herbicide inhibits acetyl coenzyme-A-carboxylase (ACC) enzyme that catalyzes the carboxylation of acetyl-CoA to malonyl-CoA, a crucial step in the de novo synthesis of fatty acids in plants [60]. Fatty acids are essential components of cell membranes and vital for plant growth and development. Malonyl-CoA depletion leads to reduced fatty acid biosynthesis and disruption in membrane integrity, cellular leakage, loss of cellular contents, and cell death [61]. 

#### Chloroacetamide

These herbicides inhibit microtubule formation, an essential component of the plant cytoskeleton involved in cell division [62]. Other mechanisms include the inhibition of elongation and photosynthesis, leading to stunted growth and decreased plant photosynthesis [63]. They also interfere with plant cell division and elongation due to inhibition of very long-chain fatty acid elongases (VLCFAE) and geranylgeranyl pyrophosphate (GGPP) cyclization enzymes [64]. Finally, these herbicides can induce oxidative stress in plants with oxidative damage in proteins, lipids, and DNA [65, 66]. 

#### Diphenylethers, Oxazolidinones, Pyrimidinediones, and N-phenylethanamides

These herbicides inhibit the protoporphyrinogen oxidase (PPO) enzyme involved in chlorophyll biosynthesis, a critical pigment for photosynthesis [67]. PPO catalyzes the conversion of protoporphyrinogen IX to protoporphyrin IX, and their inhibition leads to a buildup of protoporphyrinogen IX, oxidative stress, and cell death[68, 69].

#### Phosphinic Acids

These herbicides inhibit the activity of glutamine synthetase (GS), an essential enzyme that converts glutamate and ammonia to glutamine [70]. Excessive ammonia increases the production of ROS, decreasing pigment production and photosynthesis by inhibition of photosystem I and photosystem II reactions [49]. 

### Shared Plant-human Biology: Possible Mechanisms of Herbicide Toxicity in Humans?

Herbicides can affect human health through various nonspecific mechanisms, such as oxidative stress, inflammation, endocrine disruptors, epigenetic alterations, interference with neurotransmitter systems [71–73]. However, some biological pathways in humans can have similarities with plants and may therefore represent targets of herbicides.

#### 4-hydroxyphenylpyruvate dioxygenase - HPPD

HPPD is involved in the breakdown of tyrosine in humans and plants. Specifically, this enzyme catalyzes the conversion of 4-hydroxyphenylpyruvate into homogentisate in the tyrosine degradation pathway [74]. There are variations in the amino acid sequences and tertiary structures of HPPD in humans and plants [56]. Biochemically, human HPPD primarily functions in tyrosine catabolism, whereas plant HPPD plays a crucial role in the biosynthesis of plastoquinones, which is essential for photosynthetic electron transport. HPPD herbicide inhibitors seem to inhibit this enzyme in humans and other mammals [56, 75, 76]. In humans, HPPD deficiency is a rare metabolic disorder (hereditary tyrosinemia type 3) that leads to tyrosine accumulation [77, 78]. Although associated with neurological symptoms, the mechanisms underlying its neurotoxicity are poorly understood [79]. However, other types of tyrosinemia (types 1 and 2) have been extensively studied. While these types also result in tyrosine accumulation, they lead to the buildup of other toxic metabolites, as enzymatic defects occur at different points in the tyrosine degradation pathway [80]. Accumulation of tyrosine and its metabolites in rodent models of hereditary tyrosinemia types 1 and 2 is associated with impaired enzymatic and non-enzymatic antioxidant defenses and oxidative stress, resulting in toxicity to the eyes, liver, kidneys, and nervous system [81–84]. 

#### Acetyl-CoA Carboxylase - ACC

Both human and plant ACCs catalyze the carboxylation of acetyl-CoA to produce malonyl-CoA [85]. Human ACC exists as two isoforms, ACC1 and ACC2, each with distinct tissue distribution and cellular localization. ACC1 regulates fatty acid synthesis in lipogenic tissues, while ACC2 regulates fatty acid oxidation in the mitochondria of skeletal muscle, heart, and liver [86]. ACC1 is involved in T-cell activation and proliferation, regulating the innate immune response in macrophages, and influencing lipid metabolism in immune cells [87–89]. ACC is expressed in various tissues, and its inhibition in humans may have implications beyond metabolic health, including energy homeostasis, inflammation, and cellular signaling. Structurally, ACC in plants and humans is similar, and herbicides appear to inhibit this enzyme in mammals [85, 90, 91]. Evidence of the effects of ACC inhibition by herbicides in mammals, either in vivo or in vitro, is currently lacking. However, inhibiting ACC with lipogenesis drugs increases plasma triglyceride and very low-density lipoprotein levels, contributing to insulin resistance in both rodents and humans [92, 93]. This inhibition can also impair T-cell expansion and alter regulatory T-cell differentiation, both being critical for maintaining immune balance and response to infections in mammals [94, 95]. 

#### Glutamine Synthetase - GS

GS catalyzes glutamine biosynthesis from the condensation of glutamate and ammonia in humans and plants [96, 97]. There are no major structural differences in GS between mammals and plants [97]. Glutamine is a precursor for the base of nucleic acids, neurotransmitters, and immune function regulation [98–100]. GS inhibition in a rare disease reduces glutamine levels and can lead to hyperammonemia, and may impair immune cell activation and disrupt neurotransmitter levels, contributing to neurological disorders and immune dysregulation [101]. Additionally, the GS inhibition results in elevated extracellular glutamate levels, triggering excitotoxicity characterized by calcium overload, oxidative stress, mitochondrial dysfunction, and apoptotic cell death [102–104]. 

#### Tubulin Polymerization Inhibitors

Tubulin forms microtubules that are essential components of the cytoskeleton. In humans α- and β-tubulin isoforms, essentials for cell division and intracellular transport, are primarily expressed [105]. Plants possess similar tubulin isoforms but may have additional variants related to cell elongation and response to environmental stimuli [106]. Furthermore, post-translational modifications of tubulin, crucial for regulating its activity, may vary between humans and plants, influencing their respective cellular processes [107]. These proteins are conserved across diverse eukaryotic lineages, and herbicides inhibit tubulin polymerization in humans and other mammals [105, 108]. Tubulin inhibitors can disrupt oxidative phosphorylation, leading to mitochondrial dysfunction, oxidative stress, and cytotoxicity in mammalian cells [109]. Other in vitro studies have shown increased sister-chromatid exchanges, chromosomal aberrations, and micronuclei in human lymphocytes and mammalian cells exposed to tubulin inhibitors [110, 111]. 

#### Metabolites of Isoprenoid Synthesis

In humans, geranylgeranyl pyrophosphate (GGPP) serves as a substrate for isoprenoid enzymatic production and the post-translational modification of proteins as a precursor of vitamin K2 and ubiquinone [112, 113]. Isoprenoids are related to the prenylation of proteins that can affect immune cell activation, differentiation, and migration, potentially impacting immune responses [114]. Maintaining appropriate GGPP levels is critical for normal cellular function and human health. There is no evidence that herbicides inhibit GGPP pathway enzymes in humans. However, their potential toxic effects in humans include the disruption of the synthesis of isoprenoids, prenylated proteins, and biomolecules involved in cell signaling, and apoptosis [115, 116]. Enzymatic deficiency in the GGPP pathway in mammals has been associated with liver pathology and severe joint inflammation in mice [117, 118]. 

#### Protoporphyrinogen Oxidase – PPO

PPO is an enzyme found in humans that plays a crucial role in heme biosynthesis, an essential component of hemoglobin [119]. PPO inhibitors interfere with the conversion of protoporphyrinogen IX to protoporphyrin IX, accumulating heme synthesis intermediates and causing disorders, such as anemia and porphyria. In plants, PPO-inhibiting herbicides primarily target the chloroplast-localized PPO[120], which participates in chlorophyll biosynthesis by catalyzing the conversion of protoporphyrinogen IX from protoporphyrinogen IX[121]. However, evidence indicates that mitochondrial PPO in plants is also sensitive to herbicide effects.¹²² In this study, we performed a comparative analysis of human PPO and plant mitochondrial and chloroplast localized PPOs using protein and molecular databases, including UniProt, and the EMBOSS NEEDLE tool (Pairwise Sequence Alignment - PSA), available through the EMBL-EBI platform. This approach enables structural and functional inferences based on sequence analyses and supports comparative evaluations of conserved catalytic features (Supplementary Material) [122, 123]. The results indicate that sequence similarity between human PPO and plant mitochondrial PPO (~ 38.7%) is slightly lower than that observed for the chloroplast-localized PPO (~ 41.6%). While insufficient to infer functional equivalence, Qin et al. (2011) provided direct evidence that the herbicide acifluorfen binds within the flavin adenine dinucleotide (FAD)-dependent pocket of human PPO, as revealed by the crystal structure of human PPO in complex with FAD and acifluorfen [124]. Some studies have suggested that herbicides inhibit mammalian PPO [120, 125]. This inhibition leads to the accumulation of protoporphyrinogen IX, which interferes with mitochondrial function, impairs energy production, elevates ROS levels, and leads to intermediate filament protein aggregation [126, 127]. 

### Shared Plant-human Biology: Herbicide Effects and New Biomarkers Perspectives

Biomarkers of herbicide-induced effects are quantifiable biological changes in various human biological samples resulting from exposure to chemical substances [128]. These changes may occur before adverse effects or disease development. Ideally, they should be selective for exposure and simple to collect and measure [129]. Herbicide targets include isoprenoid and glutamine pathways, fatty acid and microtubule synthesis, and tyrosine breakdown, which are essential for humans and plants. Since these alterations in humans are uncommon[130–133], they may confer a certain selectivity to herbicide exposure, which is an important characteristic of effect biomarkers. Selectivity ensures that the biomarker of herbicide-induced effects specifically reflects herbicide exposure without interference from other environmental factors. Consequently, selective biomarkers can provide more precise assessments of health risks and facilitate early detection of adverse events. To date, specific effect biomarkers for herbicides have not been observed in the literature.

Biomarkers of effect induced by herbicides must be simple to measure. HPPD activity in blood is difficult to detect due to its low expression, mostly in the liver and kidneys [134]. However, 4-hydroxyphenylpyruvate, a substrate of HPPD, can be measured in plasma or urine by spectrophotometry and reverse-phase liquid chromatography-tandem mass spectrometry [135, 136]. By contrast, as ACC and GS are broadly expressed in tissues, including lymphocytes, they can be measured in blood [137, 138]. Their activity can be analyzed via spectrophotometer, ELISA, and enzyme assays [137, 139, 140]. Tubulin polymerization can be measured by biochemical fluorescent-based and cell cycle assays, turbidity assays to measure absorbance changes, and fluorescence microscopy [141–144]. Measuring GGPP levels is useful for studying protein prenylation and related cellular processes and can be quantified using liquid chromatography-mass spectrometry and ELISA [145–148]. PPO activity is typically measured employing spectrophotometric assays, which monitor changes in absorbance during the oxidation reaction, or with ELISA [149, 150]. 

Epidemiological studies generally have focused on associations between self-reported pesticide exposure or measured levels of pesticides/metabolites and nonspecific mechanistic markers such as oxidative stress, inflammation, and DNA damage [151–153]. Only a limited number of studies have specifically investigated markers of herbicide exposure, using either self-reported data or measurements of herbicide metabolites in biological samples [154, 155]. Herbicide metabolites or adducts in biological samples may indicate recent, past, or cumulative herbicide exposure [156–161]. Mechanistic markers signal pathophysiological changes, such as damage to cellular structures, and their association with genetic alterations or immune response dysregulation. These processes involve oxidative stress [162, 163], inflammation [164], genotoxicity [165], and immunotoxicity [166], which are the underlying mechanisms of chronic diseases including neurodegenerative, cardiovascular, respiratory, autoimmune, cancer, and other disorders linked to pesticide exposure [167]. Collectively, herbicide exposure, mechanistic biomarkers, and herbicide-induced effects are essential for a more comprehensive understanding of how herbicides impact human health.

Effect biomarkers can detect early changes related or unrelated to diseases. Certain molecular targets proposed in this seminar, such as herbicide-induced effects in humans are associated with immune and metabolic disturbances. For instance, reduced ACC activity has been linked to decreased T-cell activation and proliferation, regulation of the innate immune response in macrophages, and alterations in the lipid composition and metabolic state of immune cells. Additionally, GGPP pathway enzymes are involved in the prenylation of proteins that influence immune cell activation, differentiation, and migration [113, 114]. These enzymes are also implicated in cytokine production [168] and appear to play a critical role in modulating T-cell-mediated immune responses [169]. Furthermore, HPPD inhibitors may cause the accumulation of 4-hydroxyphenylpyruvate, leading to tyrosinemia-induced and thyroid hormone alterations via hepatic enzyme induction in mammals [75, 131]. These findings emphasize the importance of understanding the long-term direct effects of herbicides on molecular pathways and their potential role as environmental triggers or exacerbators of diseases in predisposed individuals [170, 171]. 

Despite their potential selectivity and the apparent feasibility of their measurement, some molecular targets proposed as early-effect biomarkers of herbicide exposure present important molecular-level limitations that should be acknowledged. Acetyl-CoA carboxylase in humans and plants is an enzyme that contains distinct catalytic sites associated with biotin carboxylase (BC) and carboxyltransferase (CT) activities [172–174]. In plants, ACC-inhibiting herbicides are known to primarily target the carboxyltransferase catalytic site, thereby blocking the conversion of acetyl-CoA to malonyl-CoA and impairing de novo fatty acid biosynthesis [175, 176]. Although human ACC catalyzes the same overall biochemical reaction as its plant counterpart, these enzymes exhibit important evolutionary and structural differences that warrant careful consideration [172, 177]. The literature directly comparing the herbicide-binding properties of plant and human ACC is scarce.

In this study, we performed a comparative analysis targeting the carboxyltransferase domain of ACC1, the functional site of action of ACC-inhibiting herbicides in plants (Supplementary Material). The sequence alignment analyses indicate that, while there is a moderate degree of structural similarity within the catalytic regions of the carboxyltransferase domain between plant and human ACC1, this level of conservation alone is insufficient to infer that ACC-inhibiting herbicides can inhibit human ACC1. Herbicide specificity is known to depend on subtle molecular determinants, including three-dimensional protein conformation, active-site geometry, and ligand-binding pocket architecture features that cannot be fully resolved by primary sequence similarity analyses alone. Therefore, sequence conservation in catalytic domains should not be interpreted as direct evidence of herbicide effect in humans.

### Additional Considerations

Certain herbicides target plant-specific processes, such as auxin receptors, VLCFAE enzymes, and unique biochemical pathways that do not exist in humans; yet they appear to have physiological effects in humans. For instance, phenoxyacetic herbicides have moderate toxicity, with symptoms including gastrointestinal issues (nausea, vomiting, diarrhea, abdominal pain), neurological effects (headache, dizziness, myotonia), respiratory distress, and renal dysfunction [178, 179]. Chloroacetamide herbicide poisoning can lead to severe neurological and cardiovascular effects, especially following oral ingestion [180]. While the symptoms of poisoning from these herbicides are well documented in the literature, their underlying pathophysiological mechanisms remain unclear. Additionally, the potential long-term effects of chronic, lower-level exposure to these herbicides should be considered. Epidemiological studies and animal research are required to provide biological plausibility and understand the mechanisms through which these herbicides act [181]. 

Growing evidence suggests that pesticides can impact on the gut microbiome [182]. They have been linked to alterations in the composition of gut microbiota and damage to the intestinal mucosa [183–185]. These changes may be associated with metabolic, inflammatory, and autoimmune diseases [186]. Since bacteria and fungi possess enzymes discussed in this seminar, such as HPPD, ACC, GS, and PPO (which are potential biomarkers of herbicide effects), microbiota may be affected by herbicide exposure [187–189]. In fact, herbicides appear to inhibit the growth of beneficial bacteria that are part of the gut microbiota, further altering its composition [190]. Additionally, microorganisms within the gut microbiome possess plant-like enzymes, such as ALS and EPSPS which are not found in humans [191]. – [192] This suggests that herbicides can impact a wide range of enzymes involved in different biochemical mechanisms within these organisms. The effects of herbicides on microbiota and their potential role in the development of chronic diseases should be explored in future research.

## Conclusion

This seminar highlights the potential molecular targets as biomarkers of human effects resulting from exposure to certain classes of herbicides. However, these molecular targets may also be relevant in other contexts beyond the specific focus of this seminar. Furthermore, a more comprehensive effort is needed to address both specific herbicide-induced effects and nonspecific effects, such as oxidative stress, inflammation, immunotoxicity, genotoxicity, and more recently, impacts on microbiota [193, 194]. Such efforts are crucial for guiding the development of herbicides with reduced risks to human health.

##  Key References

- Vaccari C, El Dib R, Gomaa H, Lopes LC, de Camargo JL. Paraquat and Parkinson's disease: a systematic review and meta-analysis of observational studies. J Toxicol Environ Health B Crit Rev. 2019;22(5-6):172-202. https://doi.org/10.1080/10937404.2019.1659197.
  - ◦ Systematic evidence epidemiological of herbicide effects on a neurodegenerative disease
- Shearer JJ, Sandler DP, Andreotti G, Murata K, Shrestha S, Parks CG, et al. Pesticide use and kidney function among farmers in the Biomarkers of Exposure and Effect in Agriculture study. Environ Res. 2021;199:111276. https://doi.org/10.1016/j.envres.2021.111276.
  - ◦ Evidence epidemiological of herbicide effects on kidney function
- Santucci A, Bernardini G, Braconi D, Petricci E, Manetti F. 4-Hydroxyphenylpyruvate Dioxygenase and Its Inhibition in Plants and Animals: Small Molecules as Herbicides and Agents for the Treatment of Human Inherited Diseases. J Med Chem. 2017;60(10):4101-4125. https://doi.org/10.1021/acs.jmedchem.6b01395.
  - ◦ Use of HPPD inhibitor herbicides in type I tyrosinemia
- Seng TW, Skillman TR, Yang N, Hammond C. Cyclohexanedione herbicides are inhibitors of rat heart acetyl-CoA carboxylase. Bioorg Med Chem Lett. 2003;13(19):3237-3242.https://doi.org/10.1016/s0960-894x(03)00664-4.
  - ◦ Herbicides appear to inhibit the ACC enzyme in mammals
- Krajewski WW, Collins R, Holmberg-Schiavone L, Jones TA, Karlberg T, Mowbray SL. Crystal structures of mammalian glutamine synthetases illustrate substrate-induced conformational changes and provide opportunities for drug and herbicide design. J Mol Biol. 2008;375(1):217-28.https://doi.org/10.1016/j.jmb.2007.10.029.
  - ◦ Herbicides targeting GS must be designed with caution due to subtle differences in structure of mammalian and plant enzymes
- Giglio A, Vommaro ML. Dinitroaniline herbicides: a comprehensive review of toxicity and side effects on animal non-target organisms. Environ Sci Pollut Res Int. 2022;29(51):76687-76711.https://doi.org/10.1007/s11356-022-23169-4.
  - ◦ Herbicides primarily target plant growth by disrupting microtubule formation, they can also induce adverse effects in animals
- Muehlebach ME, Holstein SA. Geranylgeranyl diphosphate synthase: Role in human health, disease and potential therapeutic target. Clin Transl Med. 2023;13(1):e1167. https://doi.org/10.1002/ctm2.1167.
  - ◦ Although there is no evidence regarding the impact of herbicides on GGPP levels, their effects should be investigated, since GGPP serves as a substrate for isoprenoid biosynthesis and the prenylation of proteins involved in immune system regulation.
- Jakubek M, Masařík M, Bříza T, Kaplánek R, Veselá K, Abramenko N, Martásek P. PPO-Inhibiting Herbicides and Structurally Relevant Schiff Bases: Evaluation of Inhibitory Activities against Human Protoporphyrinogen Oxidase. Processes. 2021; 9(2):383. https://doi.org/10.3390/pr9020383.
  - ◦ PPO-Inhibiting Herbicides may inhibit human protoporphyrinogen oxidase
- Tomoeda K, Awata H, Matsuura T, et al. Mutations in the 4-hydroxyphenylpyruvic acid dioxygenase gene are responsible for tyrosinemia type III and hawkinsinuria. Mol Genet Metab. 2000;71(3):506-510. https://doi.org/10.1006/mgme.2000.3085.
  - ◦ HPPD mutation and poor activity is found in a rare condition
- Bradberry SM, Watt BE, Proudfoot AT, Vale JA. Mechanisms of toxicity, clinical features, and management of acute chlorophenoxy herbicide poisoning: a review. J Toxicol Clin Toxicol. 2000;38(2):111-22.https://doi.org/10.1081/clt-100100925.
  - ◦ Despite targeting plant-specific pathways, some herbicides are toxic to humans
- Smith AC, Cronan JE. Dimerization of the bacterial biotin carboxylase subunit is required for acetyl coenzyme A carboxylase activity in vivo. J Bacteriol. 2012;194(1):72-8. doi: 10.1128/JB.06309-11.
  - ◦ ACC is found in microorganisms of the human gut microbiota
- Gancedo C, Holzer H. Enzymatic inactivation of glutamine synthetase in Enterobacteriaceae. Eur J Biochem. 1968;4(2):190-2. doi: 10.1111/j.1432-1033.1968.tb00192.x.
  - ◦ GS is also found in microorganisms of the human gut microbiota
- Mesnage R, Teixeira M, Mandrioli D, Falcioni L, Ducarmon QR, Zwittink RD, et al. Use of Shotgun Metagenomics and Metabolomics to Evaluate the Impact of Glyphosate or Roundup MON 52276 on the Gut Microbiota and Serum Metabolome of Sprague-Dawley Rats. Environ Health Perspect. 2021;129(1):17005. doi: 10.1289/EHP6990.
  - ◦ Glyphosate appears to alter the gut microbiota composition, with changes in the abundance of certain bacterial species and shifts in metabolic pathways related to amino acid and energy metabolism.

## Supplementary Information

Below is the link to the electronic supplementary material.

Supplementary Material 1 (PDF 2.40 MB)

Supplementary Material 2 (PDF 309 KB)

Supplementary Material 3 (DOCX 41.9 KB)

## Data Availability

This study used publicly available data retrieved from UniProt, EMBL, and FAOSTAT. These datasets are openly accessible, and no primary data were generated by the authors.
