# Supplementary material for: Shared Plant-human Biology: Herbicide Effects and New Biomarkers Perspectives
Source: Curr Environ Health Rep. 2026 Apr 2;13(1):16. doi: 10.1007/s40572-026-00523-z (PMC13046696; doi:10.1007/s40572-026-00523-z)
Supplement: Supplementary file 1 — Supplementary Material 1 (PDF 2.40 MB) [file 40572_2026_523_MOESM1_ESM.pdf]

Environmental Health Perspectives

Shared plant-human biology: herbicide effects and new biomarkers perspectives

--Manuscript Draft--

**Manuscript Number:**	EHP17631
**Full Title:**	Shared plant-human biology: herbicide effects and new biomarkers perspectives
**Article Type:**	Seminar
**Corresponding Author:**	Aline de Souza Espindola, Ph.D., MPH<br>Universidade Federal do Rio de Janeiro<br>Rio de Janeiro, RJ BRAZIL
**Corresponding Author Secondary Information:**	
**Corresponding Author's Institution:**	Universidade Federal do Rio de Janeiro
**Corresponding Author's Secondary Institution:**	
**First Author:**	Aline de Souza Espindola, Ph.D., MPH
**First Author Secondary Information:**	
**Order of Authors:**	Aline de Souza Espindola, Ph.D., MPH
	Christine G. Parks, MSPH, PhD
	Josino Costa Moreira, MSc, PhD
**Order of Authors Secondary Information:**	
**Abstract:**	Background<br><br>Herbicide exposure has been associated with acute poisonings and chronic diseases such as autoimmune diseases, cancer, neurodegenerative disorders, and renal diseases. Humans and plants share biological processes targeted by herbicides. Investigating these shared pathways can uncover crucial insights into the underlying mechanisms of herbicides in humans and identify potential biomarkers of herbicide-induced effects.<br><br>Objectives<br><br>This seminar reviews shared biochemical processes between humans and plants, particularly at the level, to identify potential biomarkers of herbicide-induced effects. Additionally, we discuss the underlying mechanisms of herbicides in human disease development.<br><br>Discussion<br><br>The tubulin concentrations and key enzymes of metabolic pathways, such as hydroxyphenylpyruvate dioxygenase (HPPD), acetyl coenzyme A carboxylase (ACC), glutamine synthetase (GS), and protoporphyrinogen oxidase (PPO) are molecular targets of herbicides in humans. They seem to have relative selectivity for exposure and can be simple to collect and measure with known techniques. This seminar highlights the importance of an in-depth research effort on enzymatic pathways to understand herbicide effects on humans from the perspective of biomarkers as a tool for early detection of biological alterations before adverse events or diseases develop.

Powered by Editorial Manager® and ProduXion Manager® from Aries Systems Corporation

Manuscript Click here to access/download;Manuscript;Seminar_Manuscript.docx

Shared plant-human biology: herbicide effects and new biomarkers perspectives

Authors: Aline de Souza Espindola MPH, PhD, Postdoctoral,[1‡] Christine G. Parks MSPH, PhD,[2†] Josino Costa Moreira MSc, PhD[1‡‡]

[1] Occupational and Environmental Health Branch, Public Health Institute, Universidade Federal do Rio de Janeiro

[2] Epidemiology Branch, National Institute of Environmental Health Sciences, National Institutes of Health, Research Triangle Park, US.

‡ Associate Professor at the Universidade Federal do Rio de Janeiro

† Research Scientist at the National Institute of Environmental Health Sciences

‡‡ Research Scientist at the Universidade Federal do Rio de Janeiro

Corresponding author:

Aline de Souza Espindola

Public Health Institute, Federal University of Rio de Janeiro

Avenida Horácio Macedo, s/n – Cidade Universitária, Rio de Janeiro – Brasil.

E-mail: esp.aline@iesc.ufrj.br

Conflict of interest disclosures

The authors declare that they have no known competing financial interests or personal relationships that could have appeared to influence the work reported in this paper.

Shared plant-human biology: herbicide effects and new biomarkers perspectives

Abstract

Background: Herbicide exposure has been associated with acute poisonings and chronic diseases such as autoimmune diseases, cancer, neurodegenerative disorders, and renal diseases. Humans and plants share biological processes targeted by herbicides. Investigating these shared pathways can uncover crucial insights into the underlying mechanisms of herbicides in humans and identify potential biomarkers of herbicide-induced effects.

Objectives: This seminar reviews shared biochemical processes between humans and plants, particularly at the level, to identify potential biomarkers of herbicide-induced effects. Additionally, we discuss the underlying mechanisms of herbicides in human disease development.

Discussion: The tubulin concentrations and key enzymes of metabolic pathways, such as hydroxyphenylpyruvate dioxygenase (HPPD), acetyl coenzyme A carboxylase (ACC), glutamine synthetase (GS), and protoporphyrinogen oxidase (PPO) are molecular targets of herbicides in humans. They seem to have relative selectivity for exposure and can be simple to collect and measure with known techniques. This seminar highlights the importance of an in-depth research effort on enzymatic pathways to understand herbicide effects on humans from the perspective of biomarkers as a tool for early detection of biological alterations before adverse events or diseases develop.

1. Introduction

Herbicides control or eliminate undesirable plants in agricultural fields, gardens, and others. Their use has risen globally, mainly due to the necessity of increasing food production and supporting the adoption of genetic modifications, leading to the development of herbicide-resistant weeds.[1-3] Indeed, in 2021, global consumption of various types of pesticides was estimated at 3,540,000 million tons, with herbicides accounting for nearly 50% of this total,[4] though use varies significantly from region to region based on agricultural practices, crop types, economic factors, and government regulations.[5-7]

Exposure to herbicides, particularly among agricultural workers, can lead to both acute and chronic health problems. Acute poisoning may result from accidental or intentional exposure, manifesting in a myriad of symptoms[8-14] that can vary depending on the type of herbicide, its concentration, and the duration of exposure.[15] In addition to acute effects, long-term occupational and environmental exposure to various herbicides has been associated with an increased risk of certain cancers, including non-Hodgkin lymphoma and colorectal cancer,[16-18] Parkinson's disease,[19] chronic kidney disease[20-22] rheumatoid arthritis, and systemic lupus erythematosus (SLE).[23,24]

Plants and humans have similar metabolic processes, such as cellular respiration and synthesizing proteins, nucleic acids, and lipids.[25-29] Exploring these shared biological processes can provide insights into the molecular targets of herbicide exposure in humans, identify potential biomarkers of herbicide-induced effects, and shed light on the underlying mechanisms of herbicide toxicity. We conducted a comprehensive review of the herbicide effects on plants and the metabolic processes shared between plants and humans, highlighting molecular targets to detect the early damage caused by herbicides.

2. Discussion

2.1 Herbicide classes and their action mechanisms in plants: a brief overview

Herbicides are used to control or eliminate undesirable plants in diverse settings, ranging from agricultural fields, along roadways, landscaping, to home gardens. Based on their chemical structure, herbicides are classified into several chemical classes (Table 1). The main herbicide mechanisms involve interferences with specific physiological or

biochemical processes within plants, disrupting their growth, development, or metabolism.

Table. 1 Herbicide chemical classes and examples.

**Chemical class***	**Examples**
Phenoxyacetic acids	2,4-dichloro phenoxy acetic acid, 2,4,5-trichloro phenoxy acetic acid, mecoprop
Pyridine carboxylic acids	Picloram, clopyralid, aminopyralid
Bipyridylium compounds	Paraquat, diquat
Imidazoline	Imazapyr, imazaquin, imazamox, imazethapyr
Triazines	Atrazine, simazine, metribuzin
Glycine derivative	Glyphosate
Isoxazoles	Isoxaflutole, pyrasulfotole
Triketones	Bicyclopyrone, sulcotrione, tembotrione
Dinitroanilines	Trifluralin, pendimethalin
Aryloxy-phenoxy-propionates	Diclofop-methyl, quizalofop-p-ethyl, clodinafop-propargyl
Chloroacetamide	Acetochlor, metolachlor
Diphenylethers	Aclonifen, nitrofen
Oxazolidinones	Pentoxazone
Pyrimidinediones	Benzfendizone, butafenacil, saflufenacil
N-phenylethanamides	Cinidon, flumiclorac, flumioxazin
Phosphinic acids	Glufosinate, bilanafos

* Adapted by Kraehmer et al. 2014[30]

2.1.1 Phenoxyacetic and pyridine carboxylic acids

These herbicide classes are auxin receptor agonists that affect normal cell elongation, root development, and plant growth.[31-33] Additionally, phenoxyacetic herbicides can disrupt cell membranes, affecting nutrient uptake, ion transport, and signal transduction.[34,35] They also interfere with chlorophyll synthesis and photosystem function, reducing the plant's ability to produce energy from sunlight and reducing growth and death.[36] Furthermore, both herbicide classes induce oxidative stress, causing damage to proteins, lipids, and DNA, which leads to tissue necrosis and cell death.[37,38]

2.1.2 Bipyridylium compounds

The main mechanisms of bipyridylium herbicides involve generating reactive oxygen species (ROS).[39] These substances are not metabolized and undergo repeated redox cycling processes, producing ROS inside cells, leading to oxidative stress and damaging cellular components. They also act targets photosystem I (PSI), specifically, ferredoxin,

preventing the reduction of nicotinamide adenine dinucleotide phosphate ($NADP^+$) to NADPH and disrupting the synthesis of ATP, an energy source in plant cells.[40] The combined effects of oxidative stress and interference with metabolic processes that use ATP led to plant death.

2.1.3 Imidazolinones

These herbicides inhibit the enzyme acetolactate synthase (ALS), a key enzyme in the biosynthesis of branched-chain amino acids valine, leucine, and isoleucine, and its inhibition leads to the disruption of protein synthesis and plant death.[41] Other toxic mechanisms for plants include impairing mitosis and cytokinesis, leading to abnormal growth patterns and cell division defects.[42] Imidazolinone herbicides can also affect chlorophyll synthesis and thylakoid membrane structure, reducing the plant's ability to absorb water and nutrients from the soil, leading to water stress and nutrient deficiency.[43-45]

2.1.4 Triazines

Triazine herbicides interfere with photosynthesis by inhibiting the enzyme photosystem II (PSII) and the photosynthetic electron transport chain, reducing ATP and NADPH synthesis.[46,47] Disruption of the electron transport chain leads to an imbalance of energy and electron flow in the chloroplasts with ROS production and oxidative stress.[48] They also interfere with pigment synthesis by inhibiting the enzyme protoporphyrinogen oxidase (PPO), leading to protoporphyrin accumulation in the cytosol and subsequent generation of ROS.[49] Taking together, the toxic effects of triazine herbicides lead to plant tissue necrosis and death.

2.1.5 Glycine derivative

Compounds derived from glycine deplete glutathione, an intracellular antioxidant that protects cells from oxidative stress.[50] It also can disrupt the antioxidant enzyme derivative of glutathione and inhibit 5-enolpyruvylshikimate-3-phosphate synthase (EPSPS), a key enzyme in the shikimate pathway.[51] EPSPS is responsible for converting phosphoenolpyruvate and shikimate-3-phosphate into 5-enolpyruvylshikimate-3-phosphate. This step is essential for synthesizing aromatic amino acids, such as tryptophan. Shikimate-3-phosphate accumulation disrupts the production of aromatic amino acids. It also induces oxidative stress by increasing ROS production and impairing

antioxidant defense mechanisms.[52,53] For example, glyphosate has been shown to disrupt mitochondrial function, leading to increased ROS generation and oxidative damage to cellular components. These effects lead to oxidative stress with lipid peroxidation, protein oxidation, and DNA damage. The consequences of glyphosate-induced oxidative stress in plants include reduced photosynthetic efficiency, altered growth patterns, and compromised stress tolerance.[54]

2.1.6 Isoxazoles and triketones

4-hydroxyphenylpyruvate dioxygenase (HPPD) impedes the conversion of 4-hydroxyphenylpyruvate, a tyrosine degradation product, to homogentisate, an important stage in the shikimate pathway, hindering the biosynthesis of tocopherol and plastoquinone.[55,56] Moreover, HPPD inhibitors also interfere with the synthesis of carotenoid pigments, which play a crucial role in protecting chlorophyll and facilitating photosynthesis.[57,58] Consequently, HPPD inhibitors can lead to uncoupling of photosynthesis, reduction of photoprotection provided by carotenoids with impaired plant growth, decreased vigor, and death.

2.1.7 Dinitroanilines

Dinitroanilines interfere with the polymerization of tubulin dimers into microtubules, a protein essential in cell division, elongation, and overall plant growth.[59] Inhibition of microtubule formation disrupts the normal process of cell division, leading to abnormalities in cell shape, cell wall synthesis, and overall plant growth. It harms root development because growing root tips have a high cell division and elongation rate.[59] Inhibition of microtubules in root cells leads to stunted root growth and abnormal root development.

2.1.8 Aryloxy-phenoxy-propionates

Aryloxy-phenoxy-propionates herbicide inhibits acetyl coenzyme-A-carboxylase (ACC) enzyme that catalyzes the carboxylation of acetyl-CoA to malonyl-CoA, a crucial step in the de novo synthesis of fatty acids in plants.[60] Fatty acids are essential components of cell membranes and vital for plant growth and development. Malonyl-CoA depletion leads to reduced fatty acid biosynthesis and disruption in membrane integrity, cellular leakage, loss of cellular contents, and cell death.[61]

2.1.9 Chloroacetamide

These herbicides inhibit microtubule formation, an essential component of the plant cytoskeleton involved in cell division.[62] Other mechanisms include the inhibition of elongation and photosynthesis, leading to stunted growth and decreased plant photosynthesis.[63] They also interfere with plant cell division and elongation due to inhibition of very long-chain fatty acid elongases (VLCFAE) and geranylgeranyl pyrophosphate (GGPP) cyclization enzymes.[64] Finally, these herbicides can induce oxidative stress in plants with oxidative damage in proteins, lipids, and DNA.[65,66]

2.1.10 Diphenylethers, oxazolidinones, pyrimidinediones, and N-phenylethanamides

These herbicides inhibit the protoporphyrinogen oxidase (PPO) enzyme involved in chlorophyll biosynthesis, a critical pigment for photosynthesis.[67] PPO catalyzes the conversion of protoporphyrinogen IX to protoporphyrin IX, and their inhibition leads to a buildup of protoporphyrinogen IX, oxidative stress, and cell death. [68,69]

2.1.11 Phosphinic acids

These herbicides inhibit the activity of glutamine synthetase (GS), an essential enzyme that converts glutamate and ammonia to glutamine.[70] Excessive ammonia increases the production of ROS, decreasing pigment production and photosynthesis by inhibition of photosystem I and photosystem II reactions.[49]

2.2 Shared plant-human biology: possible mechanisms of herbicide toxicity in humans?

Herbicides can affect human health through various nonspecific mechanisms, such as oxidative stress, inflammation, endocrine disruptors, epigenetic alterations, interference with neurotransmitter systems.[71-73] However, some biological pathways in humans can have similarities with plants and may therefore a target of herbicides.

2.2.1 HPPD

HPPD is involved in the breakdown of tyrosine in humans and plants. Specifically, this enzyme catalyzes the conversion of 4-hydroxyphenylpyruvate into homogentisate in the tyrosine degradation pathway.[74] There are variations in the amino acid sequences and tertiary structures of HPPD in humans and plants.[56] Biochemically, human HPPD

primarily functions in tyrosine catabolism, whereas plant HPPD plays a crucial role in the biosynthesis of plastoquinones, which is essential for photosynthetic electron transport. HPPD herbicide inhibitors seem to inhibit this enzyme in humans and other mammals.[56,75,76] In humans, HPPD deficiency is a rare metabolic disorder (hereditary tyrosinemia type 3) that leads to tyrosine accumulation.[77,78] Although associated with neurological symptoms, the mechanisms underlying its neurotoxicity are poorly understood.[79] However, other types of tyrosinemia (types 1 and 2) have been extensively studied. While these types also result in tyrosine accumulation, they lead to the buildup of other toxic metabolites, as enzymatic defects occur at different points in the tyrosine degradation pathway.[80] Accumulation of tyrosine and its metabolites in rodent models of hereditary tyrosinemia types 1 and 2 is associated with impaired enzymatic and non-enzymatic antioxidant defenses and oxidative stress, resulting in toxicity to the eyes, liver, kidneys, and nervous system.[81-84]

2.2.2 ACC

Both human and plant ACCs catalyze the carboxylation of acetyl-CoA to produce malonyl-CoA.[85] Human ACC exists as two isoforms, ACC1 and ACC2, each with distinct tissue distribution and cellular localization. ACC1 regulates fatty acid synthesis in lipogenic tissues, while ACC2 regulates fatty acid oxidation in the mitochondria of skeletal muscle, heart, and liver.[86] ACC1 is involved in T-cell activation and proliferation, regulating the innate immune response in macrophages, and influencing lipid metabolism in immune cells.[87-89] ACC is expressed in various tissues, and its inhibition in humans may have implications beyond metabolic health, including energy homeostasis, inflammation, and cellular signaling. Structurally, ACC in plants and humans is similar, and herbicides appear to inhibit this enzyme in mammals.[85,90,91] Evidence of the effects of ACC inhibition by herbicides in mammals, either in vivo or in vitro, is currently lacking. However, inhibiting ACC with lipogenesis drugs increases plasma triglyceride and very low-density lipoprotein levels, contributing to insulin resistance in both rodents and humans.[92,93] This inhibition can also impair T-cell expansion and alter regulatory T-cell differentiation, both being critical for maintaining immune balance and response to infections in mammals.[94,95]

2.2.3 GS

GS catalyzes glutamine biosynthesis from the condensation of glutamate and ammonia in humans and plants.[96,97] There are no major structural differences in GS between mammals and plants.[97] Glutamine is a precursor for the base of nucleic acids, neurotransmitters, and immune function regulation.[98-100] GS inhibition in a rare disease reduces glutamine levels and can lead to hyperammonemia, and may impair immune cell activation and disrupt neurotransmitter levels, contributing to neurological disorders and immune dysregulation.[101] Additionally, the GS inhibition results in elevated extracellular glutamate levels, triggering excitotoxicity characterized by calcium overload, oxidative stress, mitochondrial dysfunction, and apoptotic cell death.[102-104]

2.2.4 Tubulin polymerization inhibitors

Tubulin forms microtubules that are essential components of the cytoskeleton. In humans α- and β-tubulin isoforms, essentials for cell division and intracellular transport, are primarily expressed.[105] Plants possess similar tubulin isoforms but may have additional variants related to cell elongation and response to environmental stimuli.[106] Furthermore, post-translational modifications of tubulin, crucial for regulating its activity, may vary between humans and plants, influencing their respective cellular processes.[107] These proteins are conserved across diverse eukaryotic lineages, and herbicides inhibit tubulin polymerization in humans and other mammals.[105,108] Tubulin inhibitors can disrupt oxidative phosphorylation, leading to mitochondrial dysfunction, oxidative stress, and cytotoxicity in mammalian cells.[109] Other in vitro studies have shown increased sister-chromatid exchanges, chromosomal aberrations, and micronuclei in human lymphocytes and mammalian cells exposed to tubulin inhibitors.[110,111]

2.2.5 Metabolites of isoprenoid synthesis

In humans, GGPP serves as a substrate for isoprenoid enzymatic production and the post-translational modification of proteins as a precursor of vitamin K2 and ubiquinone.[112,113] Isoprenoids are related to the prenylation of proteins that can affect immune cell activation, differentiation, and migration, potentially impacting immune responses.[114] Maintaining appropriate GGPP levels is critical for normal cellular function and human health. There is no evidence that herbicides inhibit GGPP pathway enzymes in humans. However, their potential toxic effects in humans include the disruption of the synthesis of isoprenoids, prenylated proteins, and biomolecules involved in cell signaling, and

apoptosis.[115,116] Enzymatic deficiency in the GGPP pathway in mammals has been associated with liver pathology and severe joint inflammation in mice.[117,118]

2.2.6 PPO inhibitors

PPO is an enzyme found in humans that plays a crucial role in heme biosynthesis, an essential component of hemoglobin.[119] PPO inhibitors interfere with the conversion of protoporphyrinogen IX to protoporphyrin IX, accumulating heme synthesis intermediates and causing disorders, such as anemia and porphyria. In plants, PPO participates in chlorophyll biosynthesis by catalyzing the formation of protoporphyrinogen IX from protoporphyrinogen III.[120] Some studies have suggested that herbicides inhibit mammalian PPO.[120,121] PPO inhibition leads to the accumulation of protoporphyrinogen IX, which interferes with mitochondrial function, impairs energy production, elevates ROS levels, and leads to intermediate filament protein aggregation in mammals.[122,123]

2.3 Shared plant-human biology: herbicide effects and new biomarkers perspectives

Biomarkers of herbicide-induced effects are quantifiable biological changes in various human biological samples resulting from exposure to chemical substances.[124] These changes may occur before adverse effects or disease development. Ideally, they should be selective for exposure and simple to collect and measure.[124] Herbicide targets include isoprenoid and glutamine pathways, fatty acid and microtubule synthesis, and tyrosine breakdown, which are essential for humans and plants. Since these alterations in humans are uncommon,[125-128] they may confer a certain selectivity to herbicide exposure, which is an important characteristic of effect biomarkers. Selectivity ensures that the biomarker of herbicide-induced effects specifically reflects herbicide exposure without interference from other environmental factors. Consequently, selective biomarkers can provide more precise assessments of health risks and facilitate early detection of adverse events. To date, specific effect biomarkers for herbicides have not been observed in the literature.

Biomarkers of effect induced by herbicides must be simple to measure. HPPD activity in blood is difficult to detect due to its low expression, mostly in the liver and kidneys.[129] However, 4-hydroxyphenylpyruvate, a substrate of HPPD, can be measured in plasma or urine by spectrophotometry and reverse-phase liquid chromatography-tandem mass spectrometry.[130,131] By contrast, as ACC and GS are broadly expressed in tissues, including lymphocytes, they can be measured in blood.[132,133] Their activity can be

analyzed via spectrophotometer, ELISA, and enzyme assays.[132,134,135] Tubulin polymerization can be measured by biochemical fluorescent-based and cell cycle assays, turbidity assays to measure absorbance changes, and fluorescence microscopy.[136-139] Measuring GGPP levels is useful for studying protein prenylation and related cellular processes and can be quantified using liquid chromatography-mass spectrometry and ELISA.[140-143] PPO activity is typically measured employing spectrophotometric assays, which monitor changes in absorbance during the oxidation reaction, or with ELISA.[144,145] Epidemiological studies generally have focused on associations between self-reported pesticide exposure or measured levels of pesticides/metabolites and nonspecific mechanistic markers such as oxidative stress, inflammation, and DNA damage.[146-148] Only a limited number of studies have specifically investigated markers of herbicide exposure, using either self-reported data or measurements of herbicide metabolites in biological samples.[149-150] Herbicide metabolites or adducts in biological samples may indicate recent, past, or cumulative herbicide exposure.[151-156] Mechanistic markers signal pathophysiological changes, such as damage to cellular structures, and their association with genetic alterations or immune response dysregulation. These processes involve oxidative stress[157,158], inflammation[159], genotoxicity,[160,] and immunotoxicity[161], which are the underlying mechanisms of chronic diseases including neurodegenerative, cardiovascular, respiratory, autoimmune, cancer, and other disorders linked to pesticide exposure.[162] Collectively, herbicide exposure, mechanistic biomarkers, and herbicide-induced effects are essential for a more comprehensive understanding of how herbicides impact human health.

Effect biomarkers can detect early changes related or unrelated to diseases. Certain molecular targets proposed in this seminar, such as herbicide-induced effects in humans are associated with immune and metabolic disturbances. For instance, reduced ACC activity has been linked to decreased T-cell activation and proliferation, regulation of the innate immune response in macrophages, and alterations in the lipid composition and metabolic state of immune cells.[163-165] Additionally, GGPP pathway enzymes are involved in the prenylation of proteins that influence immune cell activation, differentiation, and migration.[166,167] These enzymes are also implicated in cytokine production[168] and appear to play a critical role in modulating T-cell-mediated immune responses.[169] Furthermore, HPPD inhibitors may cause the accumulation of 4-hydroxyphenylpyruvate, leading to tyrosinemia-induced and thyroid hormone alterations via hepatic enzyme induction in mammals.[75,127] These findings emphasize the importance

of understanding the long-term direct effects of herbicides on molecular pathways and their potential role as environmental triggers or exacerbators of diseases in predisposed individuals.[170,171]

2.4 Additional considerations

Certain herbicides target plant-specific processes, such as auxin receptors, VLCFAE enzymes, and unique biochemical pathways that do not exist in humans; yet they appear to have physiological effects in humans. For instance, phenoxyacetic herbicides have moderate toxicity, with symptoms including gastrointestinal issues (nausea, vomiting, diarrhea, abdominal pain), neurological effects (headache, dizziness, myotonia), respiratory distress, and renal dysfunction.[172-174] Chloroacetamide herbicide poisoning can lead to severe neurological and cardiovascular effects, especially following oral ingestion.[175] While the symptoms of poisoning from these herbicides are well documented in the literature, their underlying pathophysiological mechanisms remain unclear. Additionally, the potential long-term effects of chronic, lower-level exposure to these herbicides should be considered. Epidemiological studies and animal research are required to provide biological plausibility and understand the mechanisms through which these herbicides act.[176]

Growing evidence suggests that pesticides can impact on the gut microbiome.[177] They have been linked to alterations in the composition of gut microbiota and damage to the intestinal mucosa.[178-180] These changes may be associated with metabolic, inflammatory, and autoimmune diseases.[181] Since bacteria and fungi possess enzymes discussed in this seminar, such as HPPD, ACC, GS, and PPO (which are potential biomarkers of herbicide effects), microbiota may be affected by herbicide exposure.[182-184] In fact, herbicides appear to inhibit the growth of beneficial bacteria that are part of the gut microbiota, further altering its composition.[185] Additionally, microorganisms within the gut microbiome possess plant-like enzymes, such as ALS and EPSPS which are not found in humans.[186-187] This suggests that herbicides can impact a wide range of enzymes involved in different biochemical mechanisms within these organisms. The effects of herbicides on microbiota and their potential role in the development of chronic diseases should be explored in future research.

This seminar highlights the potential molecular targets as biomarkers of human effects resulting from exposure to certain classes of herbicides. However, these molecular targets

may also be relevant in other contexts beyond the specific focus of this seminar. Furthermore, a more comprehensive effort is needed to address both specific herbicide-induced effects and nonspecific effects, such as oxidative stress, inflammation, immunotoxicity, genotoxicity, and more recently, impacts on the microbiota.[188,189] Such efforts are crucial for guiding the development of herbicides with reduced risks to human health.

Acknowledgments

The Isabella Gaichi Romaguera Giannini, Undergraduate student in Biomedicine at Universidade Federal do Estado do Rio de Janeiro, was instrumental in gathering relevant references on "herbicide classes and their mechanisms of action in plants," as well as on "symptoms of acute herbicide poisoning," primarily from the PubMed and ScienceDirect databases, utilizing Advanced Search.

References

1. Benbrook CM. Impacts of genetically engineered crops on pesticide use in the U.S. - the first sixteen years. Environ Sci Eur. 2012; 24, 24. https://doi.org/10.1186/2190-4715-24-24
2. Kniss AR. Long-term trends in the intensity and relative toxicity of herbicide use. Nat Commun. 2017;8:14865. https://doi.org/10.1038/ncomms14865
3. Ofosu R, Agyemang ED, Márton A, Pásztor G, Taller J, Kazinczi G. Herbicide Resistance: Managing Weeds in a Changing World. Agronomy. 2023; 13(6):1595. https://doi.org/10.3390/agronomy13061595
4. Food and Agriculture Organization of the United Nations (FAO). FAOSTAT analytical brief 70: pesticides use and trade 1990–2021 [Internet]. Rome: FAO; 2023 [cited 2025 Mar 12]. Available from: [https://openknowledge.fao.org/server/api/core/bitstreams/222f250c-3764-401b-98c7-f52a699dd65c/contente]
5. Haggblade S, Minten B, Pray C, Reardon T, Zilberman D. The Herbicide Revolution in Developing Countries: Patterns, Causes, and Implications. Eur J Dev Res. 2017; 29, 533-559. https://doi.org/10.1057/s41287-017-0090-7
6. Sarkar S, Gil JDB, Keeley J, Jansen K. The use of pesticides in developing countries and their impact on health and the right to food. European Union. 2021. Available from: https://www.europarl.europa.eu/thinktank/en/document/EXPO_STU(2021)653622
7. Desquilbet M, Bullock DS, d'Arcangelo, FM. A discussion of the market and policy failures associated with the adoption of herbicide-tolerant crops. Int. J. Agric. Sustain. 2019; 17(5), 326-337. https://doi.org/10.1080/14735903.2019.1655191
8. Faria NMX, Meucci RD, Fiori NS, Carret MLV, Mello-da-Silva CA, Fassa AG. Acute Pesticide Poisoning in Tobacco Farming, According to Different Criteria. Int J Environ Res Public Health. 2023;20(4):2818. https://doi.org/10.3390/ijerph20042818

9. Sukumar CA, Shanbhag V, Shastry AB. Paraquat: The Poison Potion. Indian J Crit Care Med. 2019;23(Suppl 4):S263-S266. https://doi.org/10.5005/jp-journals-10071-23306

10. Bradberry SM, Proudfoot AT, Vale JA. Poisoning due to chlorophenoxy herbicides. Toxicol Rev. 2004;23(2):65-73. https://doi.org/10.2165/00139709-200423020-00001

11. Brvar M, Okrajsek R, Kosmina P, Staric F, Kaps R, Kozelj G, Bunc M. Metabolic acidosis in prometryn (triazine herbicide) self-poisoning. Clin Toxicol (Phila). 2008;46(3):270-3. https://doi.org/10.1080/15563650701665126

12. Huang Y, Zhang R, Meng M, Chen D, Deng Y. High-dose diquat poisoning: a case report. J Int Med Res. 2021;49(6):3000605211026117. https://doi.org/10.1177/03000605211026117

13. Pannu AK, Saroch A, Agrawal J, Sharma N. 2,4-D poisoning: a review with illustration of two cases. Trop Doct. 2018;48(4):366-368. https://doi.org/10.1177/0049475518786834

14. Suekane A, Edamoto M, Kubo K, Abe T, Nakatsutsumi K, Ameda T. Metolachlor Poisoning with Lactic Acidosis Improved by Thiamine Administration: A Case Report. Am J Case Rep. 2022;23:e937873. https://doi.org/10.12659/AJCR.937873

15. Buralli RJ, Ribeiro H, Iglesias V, Muñoz-Quezada MT, Leão RS, Marques RC, Almeida MMC, Guimarães JRD. Occupational exposure to pesticides and health symptoms among family farmers in Brazil. Rev Saude Publica. 2020;54:133. doi: 10.11606/s1518-8787.2020054002263.

16. Zhang L, Rana I, Shaffer RM, Taioli E, Sheppard L. Exposure to glyphosate-based herbicides and risk for non-Hodgkin lymphoma: A meta-analysis and supporting evidence. Mutat Res Rev Mutat Res. 2019;781:186-206. https://doi.org/10.1016/j.mrrev.2019.02.001

17. Matich EK, Laryea JA, Seely KA, Stahr S, Su LJ, Hsu PC. Association between pesticide exposure and colorectal cancer risk and incidence: A systematic review. Ecotoxicol Environ Saf. 2021;219:112327. https://doi.org/10.1016/j.ecoenv.2021.112327

18. De Roos AJ, Fritschi L, Ward MH, Monnereau A, Hofmann J, Bernstein L, Bhatti P, et al. Herbicide use in farming and other jobs in relation to non-Hodgkin's lymphoma (NHL) risk. Occup Environ Med. 2022;79(12):795-806. https://doi.org/10.1136/oemed-2022-108371

19. Vaccari C, El Dib R, Gomaa H, Lopes LC, de Camargo JL. Paraquat and Parkinson's disease: a systematic review and meta-analysis of observational studies. J Toxicol Environ Health B Crit Rev. 2019;22(5-6):172-202. https://doi.org/10.1080/10937404.2019.1659197

20. Shearer JJ, Sandler DP, Andreotti G, Murata K, Shrestha S, Parks CG, Liu D, Alavanja MC, Landgren O, Beane Freeman LE, Hofmann JN. Pesticide use and kidney function among farmers in the Biomarkers of Exposure and Effect in Agriculture study. Environ Res. 2021;199:111276. https://doi.org/10.1016/j.envres.2021.111276

21. Jayasumana C, Paranagama P, Agampodi S, Wijewardane C, Gunatilake S, Siribaddana S. Drinking well water and occupational exposure to Herbicides is associated with chronic kidney disease, in Padavi-Sripura, Sri Lanka. Environ Health. 2015;14:6. https://doi.org/10.1186/1476-069X-14-6

22. Lozano-Paniagua D, Parrón T, Alarcón R, Requena M, Lacasaña M, Hernández AF. Renal tubular dysfunction in greenhouse farmers exposed to pesticides unveiled by a panel of molecular biomarkers of kidney injury. Environ Res. 2023;238(Pt 2):117200. https://doi.org/10.1016/j.envres.2023.117200

23. Meyer A, Sandler DP, Beane Freeman LE, Hofmann JN, Parks CG. Pesticide Exposure and Risk of Rheumatoid Arthritis among Licensed Male Pesticide Applicators in the Agricultural Health Study. Environ Health Perspect. 2017;125(7):077010. https://doi.org/10.1289/EHP1013

24. Parks CG, Costenbader KH, Long S, Hofmann JN, Beane FLE, Sandler DP. Pesticide use and risk of systemic autoimmune diseases in the Agricultural Health Study. Environ Res. 2022;209:112862. https://doi.org/10.1016/j.envres.2022.112862

25. Liu X, Li M, Li Y, Chen Z, Zhuge C, Ouyang Y, Zhao Y, Lin Y, Xie Q, Yang C, Lai J. An ABHD17-like hydrolase screening system to identify de-S-acylation enzymes of protein substrates in plant cells. The Plant cell.2021;33,10: 3235-3249. https://doi.org/10.1093/plcell/koab199
26. Stiller, John W. Emerging genomic and proteomic evidence on relationships among the animal, plant and fungal kingdoms. Genomics, proteomics & bioinformatics vol. 2,2 (2004): 70-6. https://doi.org/10.1016/s1672-0229(04)02012-1
27. Lee JM, Lee H, Kang S, Park WJ. Fatty acid desaturases, polyunsaturated fatty acid regulation, and biotechnological advances. Nutrients. 2016;8(1):23. https://doi.org/10.3390/nu8010023
28. Jiménez C, Cossío BR, Rivard CJ, Berl T, Capasso JM. Cell division in the unicellular microalga Dunaliella viridis depends on phosphorylation of extracellular signal-regulated kinases (ERKs). J Exp Bot. 2007;58(5):1001-11. https://doi.org/10.1093/jxb/erl260
29. Lehti-Shiu, Melissa D, Shin-Han Shiu. Diversity, classification and function of the plant protein kinase superfamily. Philosophical transactions of the Royal Society of London. Series B, Biological sciences. 2012;367(1602): 2619-39. https://doi.org/10.1098/rstb.2012.0003
30. Kraehmer H, Laber B, Rosinger C, Schulz A. Herbicides as weed control agents: state of the art: I. Weed control research and safener technology: the path to modern agriculture. Plant Physiol. 2014;166(3):1119-31. https://doi.org/10.1104/pp.114.241901
31. Lushchak VI, Matviishyn TM, Husak VV, Storey JM, Storey KB. Pesticide toxicity: a mechanistic approach. EXCLI journal. 2018;17:1101-1136. https://doi.org/10.17179/excli2018-1710
32. Liu X, Qi C, Wang Z, Li Y, Wang Q, Guo M, Cao, A. Effect of picloram herbicide on physiological responses of Eupatorium adenophorum Spreng. Chilean journal of agricultural research. 2014; 74(4), 438-444.
33. Satoh S, Nomura Y. (2017). Promotion of root elongation by pyridine carboxylic acids known as novel cut flower care agents. Plant Root.2017;11, 40-47.
34. Chinalia FA, Regali-Seleghin MH, Correa EM. 2, 4-D toxicity: cause, effect and control. Terrestrial and Aquatic Environmental Toxicology. 2007;1(2):24-33.
35. Goggin DE, Cawthray GR, Powles SB. 2,4-D resistance in wild radish: reduced herbicide translocation via inhibition of cellular transport. J Exp Bot. 2016;67(11):3223-35. https://doi.org/10.1093/jxb/erw120
36. Pazmiño DM, Romero-Puertas MC, Sandalio LM. Insights into the toxicity mechanism of and cell response to the herbicide 2,4-D in plants. Plant signaling & behavior. 2012;7(3):425-7. https://doi.org/10.4161/psb.19124
37. Bernat P, Nykiel-Szymańska J, Stolarek P, Słaba M, Szewczyk R, Różalska S. 2,4-dichlorophenoxyacetic acid-induced oxidative stress: Metabolome and membrane modifications in Umbelopsis isabellina, a herbicide degrader. PLoS One. 2018;13(6):e0199677. https://doi.org/10.1371/journal.pone.0199677
38. Hassannejad S, Abbasvand E, Fadaei B, Shiri N, Nasirpour Z, Ghafarbi SP. Survey of salicylic acid effect on Clopyralid efficiency in control of Field bindweed (Convolvulus arvensis L.). Research Square, 2023. https://doi.org/10.21203/rs.3.rs-2875920/v1
39. Li J, Mu J, Bai J, Fu F, Zou T, An F, Zhang J, Jing H, Wang Q, Li Z, Yang S, Zuo J. Paraquat Resistant1, a Golgi-localized putative transporter protein, is involved in intracellular transport of paraquat. Plant Physiol. 2013;162(1):470-83. https://doi.org/10.1104/pp.113.213892
40. Hawkes TR. Mechanisms of resistance to paraquat in plants. Pest Manag Sci. 2014;70(9):1316-23. https://doi.org/10.1002/ps.3699

41. Zhou Q, Liu W, Zhang Y, Liu KK. Action mechanisms of acetolactate synthase-inhibiting herbicides. Pestic Biochem Physiol. 2007;89(2):89-96. https://doi.org/doi:10.1016/j.pestbp.2007.04.004
42. Liman R, Ciğerci İH, Öztürk NS. Determination of genotoxic effects of Imazethapyr herbicide in Allium cepa root cells by mitotic activity, chromosome aberration, and comet assay. Pestic Biochem Physiol. 2015;118:38-42. https://doi.org/10.1016/j.pestbp.2014.11.007
43. Qian H, Han X, Zhang Q, Sun Z, Sun L, Fu Z. Imazethapyr enantioselectively affects chlorophyll synthesis and photosynthesis in Arabidopsis thaliana. J Agric Food Chem. 2013;61(6):1172-8. https://doi.org/10.1021/jf305198g
44. Balabanova DA, Paunov M, Goltsev V, Cuypers A, Vangronsveld J, Vassilev A. Photosynthetic Performance of the Imidazolinone Resistant Sunflower Exposed to Single and Combined Treatment by the Herbicide Imazamox and an Amino Acid Extract. Front Plant Sci. 2016;7:1559. https://doi.org/10.3389/fpls.2016.01559
45. Manabe Y, Tinker N, Colville A, Miki B. CSR1, the sole target of imidazolinone herbicide in Arabidopsis thaliana. Plant Cell Physiol. 2007;48(9):1340-58. https://doi.org/10.1093/pcp/pcm105. Erratum in: Plant Cell Physiol. 2007;48(11):1658.
46. Trebst A. Chapter 8 - The Mode of Action of Triazine Herbicides in Plants. The Triazine Herbicides. Elsevier, 2008:101-110. https://doi.org/10.1016/B978-044451167-6.50011-8
47. Battaglino B, Grinzato A, Pagliano C. Binding Properties of Photosynthetic Herbicides with the QB Site of the D1 Protein in Plant Photosystem II: A Combined Functional and Molecular Docking Study. Plants (Basel). 2021;10(8):1501. https://doi.org/10.3390/plants10081501
48. Wilson PC, Whitwell T, Klaine SJ. Simazine toxicity and uptake by parrotfeather. J. Aquat. Plant Manage. 2001; 39, 112-116.
49. Traxler C, Gaines TA, Küpper A, Luemmen P, Dayan FE. The nexus between reactive oxygen species and the mechanism of action of herbicides. J Biol Chem. 2023;299(11):105267. https://doi.org/10.1016/j.jbc.2023.105267
50. Miteva, L.P.E., Ivanov, S.V. & Alexieva, V.S. Alterations in glutathione pool and some related enzymes in leaves and roots of pea plants treated with the herbicide glyphosate. Russ J Plant Physiol 57, 131–136 (2010). https://doi.org/10.1134/S1021443710010188
51. Gomes MP, Smedbol E, Chalifour A, Hénault-Ethier L, Labrecque M, Lepage L, Lucotte M, Juneau P. Alteration of plant physiology by glyphosate and its by-product aminomethylphosphonic acid: an overview. J Exp Bot. 2014;65(17):4691-703. https://doi.org/10.1093/jxb/eru269
52. Eceiza MV, Gil-Monreal M, Barco-Antoñanzas M, Zabalza A, Royuela M. The moderate oxidative stress induced by glyphosate is not detected in Amaranthus palmeri plants overexpressing EPSPS. J Plant Physiol. 2022;274:153720. https://doi.org/10.1016/j.jplph.2022.153720
53. Bortolheiro FPDAP, Brunelli-Nascentes MC, Santos HL, de Almeida Silva M. Increased hormetic dose of glyphosate causes oxidative stress and reduces yield in common bean. Plant Stress. 2023;10, 100231
54. Hoagland RE. Effects of Glyphosate on Metabolism of Phenolic Compounds: VI. Effects of Glyphosine and Glyphosate Metabolites on Phenylalanine Ammonia-Lyase Activity, Growth, and Protein, Chlorophyll, and Anthocyanin Levels in Soybean (Glycine max) Seedlings. Weed Science. 1980;28(4):393-400. https://doi.org/10.1017/S0043174500055545
55. Parthasarathy A, Cross PJ, Dobson RCJ, Adams LE, Savka MA, Hudson AO. A Three-Ring Circus: Metabolism of the Three Proteogenic Aromatic Amino Acids and Their Role in the

Health of Plants and Animals. Front Mol Biosci. 2018;5:29. https://doi.org/10.3389/fmolb.2018.00029

56. Santucci A, Bernardini G, Braconi D, Petricci E, Manetti F. 4-Hydroxyphenylpyruvate Dioxygenase and Its Inhibition in Plants and Animals: Small Molecules as Herbicides and Agents for the Treatment of Human Inherited Diseases. J Med Chem. 2017;60(10):4101-4125. https://doi.org/10.1021/acs.jmedchem.6b01395

57. Ahrens H, Lange G, Müller T, Rosinger C, Willms L, van Almsick A. 4-Hydroxyphenylpyruvate dioxygenase inhibitors in combination with safeners: solutions for modern and sustainable agriculture. Angew Chem Int Ed Engl. 2013;52(36):9388-98. https://doi.org/10.1002/anie.201302365

58. Jhala A, Kumar V, Yadav R, Jha P, Jugulam M, Williams M, et al. 4-Hydroxyphenylpyruvate dioxygenase (HPPD)-inhibiting herbicides: Past, present, and future. Weed Technology, 2023; 37(1):1-14. https://doi.org/10.1017/wet.2022.79

59. Chen J, Yu Q, Patterson E, Sayer C, Powles S. Dinitroaniline Herbicide Resistance and Mechanisms in Weeds. Front Plant Sci. 2021 Mar 25;12:634018. doi: 10.3389/fpls.2021.634018.

60. Rajak BK, Rani P, Mandal P, Chhokar RS, Singh N and Singh DV (2023) Emerging possibilities in the advancement of herbicides to combat acetyl-CoA carboxylase inhibitor resistance. Front. Agron. 5:1218824. https://doi.org/10.3389/fagro.2023.1218824

61. Takano HK, Ovejero RFL, Belchior GG, Maymone GPL, Dayan FE. (2020). ACCase-inhibiting herbicides: mechanism of action, resistance evolution and stewardship. Scientia Agricola, 78, e20190102.

62. Quanrud GM, Lyu Z, Balamurugan SV, Canizal C, Wu HT, Genereux JC. Cellular Exposure to Chloroacetanilide Herbicides Induces Distinct Protein Destabilization Profiles. ACS Chem Biol. 2023;18(7):1661-1676. https://doi.org/10.1021/acschembio.3c00338

63. Böger P, Matthes B, Schmalfuß J. Towards the primary target of chloroacetamides–new findings pave the way. Pest Management Science: formerly Pesticide Science, 2000; 56(6), 497-508.

64. Giardina A, Tampieri F, Biondo O, Marotta E, Paradisi C. Air non-thermal plasma treatment of the herbicides mesotrione and metolachlor in water. Chemical Engineering Journal, 2019; 372:171-180. https://doi.org/10.1016/j.cej.2019.04.098.

65. Bonnet JL, Bonnemoy F, Dusser M, Bohatier J. Assessment of the potential toxicity of herbicides and their degradation products to nontarget cells using two microorganisms, the bacteria Vibrio fischeri and the ciliate Tetrahymena pyriformis. Environ Toxicol. 2007;22(1):78-91. https://doi.org/10.1002/tox.20237

66. Rose MT, Cavagnaro TR, Scanlan CA, Rose TJ, Vancov T, Kimber S, Kennedy IR, Kookana Rai S, Van Zwieten L. Impact of Herbicides on Soil Biology and Function. Advances in Agronomy. 2016; (136):133-220. https://doi.org/10.1016/bs.agron.2015.11.005

67. Duke SO. Overview of herbicide mechanisms of action. Environ Health Perspect. 1990;87:263-71. https://doi.org/10.1289/ehp.9087263

68. Arnould, S, and J M Camadro. The domain structure of protoporphyrinogen oxidase, the molecular target of diphenyl ether-type herbicides. Proceedings of the National Academy of Sciences of the United States of America.1998;95(18): 10553-8. https://doi.org/10.1073/pnas.95.18.10553

69. Alves C, Costa E, Sofiatti JR, Forte CT, Winter FL, Holz CM, Kaizer RR, Galon L. Effect of herbicides in the oxidative stress in crop winter species. An Acad Bras Cienc. 2018;90(2):1533-1542. https://doi.org/10.1590/0001-3765201820170482

70. Vallejo B, Picazo C, Orozco H, Matallana E, Aranda A. Herbicide glufosinate inhibits yeast growth and extends longevity during wine fermentation. Sci Rep. 2017;7(1):12414. https://doi.org/10.1038/s41598-017-12794-6
71. McBirney M, King SE, Pappalardo M, Houser E, Unkefer M, Nilsson E, Sadler-Riggleman I, Beck D, Winchester P, Skinner MK. Atrazine induced epigenetic transgenerational inheritance of disease, lean phenotype and sperm epimutation pathology biomarkers. PLoS One. 2017;12(9):e0184306. https://doi.org/10.1371/journal.pone.0184306
72. María-Aránzazu Martínez, Irma Ares, José-Luis Rodríguez, Marta Martínez, María-Rosa Martínez-Larrañaga, Arturo Anadón. Neurotransmitter changes in rat brain regions following glyphosate exposure. Environmental Research.2018; 161:212-219.
73. Sengupta A, Manna K, Datta S, Das U, Biswas S, Chakrabarti N, Dey S. Herbicide exposure induces apoptosis, inflammation, immune modulation and suppression of cell survival mechanism in murine model. RSC advances. 2017; 7(23):13957-13970.
74. Moran GR. 4-Hydroxyphenylpyruvate dioxygenase. Arch Biochem Biophys. 2005;433(1):117-28. https://doi.org/10.1016/j.abb.2004.08.015
75. Antonenko AM, Blagaia AV, Omelchuk ST, Korshun MM, Vavrinevych HP, Milohov DS, Pelo IM, Bojar I. Mechanism of action of 4-hydroxyphenylpyruvate dioxygenase inhibitor herbicide on homoterm animals and humans. J Pre Clin Clin Res. 2015;9(2):145-150. https://doi.org/10.5604/18982395.1186496
76. Wu N. Herbicide sulcotrione. Herbicides, Theory and Applications. Soloneski S, Larramendy ML, Eds. InTech, 2011:527–544.
77. Najafi R, Mostofizadeh N, Hashemipour M. A Case of Tyrosinemia Type III with Status Epilepticus and Mental Retardation. Adv Biomed Res. 2018;7:7. https://doi.org/10.4103/2277-9175.223740
78. Vakili S, Emami M, Mobini M, Vakili R. Tyrosinemia Type III: A Case Report with a Seven Years Follow-up. J Pediatr Perspect. 2021;9(6):13853-13857. https://doi.org/10.22038/ijp.2021.56791.4451
79. Barroso F, Correia J, Bandeira A, Carmona C, Vilarinho L, Almeida M, Rocha JC, Martins E. Tyrosinemia type iii: a case report of siblings and literature review. Rev Paul Pediatr. 2020;38:e2018158. https://doi.org/10.1590/1984-0462/2020/38/2018158
80. Scott CR. The genetic tyrosinemias. Am J Med Genet C Semin Med Genet. 2006;142C(2):121-6. https://doi.org/10.1002/ajmg.c.30092
81. Colemonts-Vroninks H, Neuckermans J, Marcelis L, Claes P, Branson S, Casimir G, Goyens P, Martens GA, Vanhaecke T, De Kock J. Oxidative Stress, Glutathione Metabolism, and Liver Regeneration Pathways Are Activated in Hereditary Tyrosinemia Type 1 Mice upon Short-Term Nitisinone Discontinuation. Genes (Basel). 2020;12(1):3. https://doi.org/10.3390/genes12010003
82. de Oliveira J, Farias HR, Streck EL. Experimental evidence of tyrosine neurotoxicity: focus on mitochondrial dysfunction. Metab Brain Dis. 2021;36(7):1673-1685. https://doi.org/10.1007/s11011-021-00781-w
83. Lewis RW, Botham JW. A review of the mode of toxicity and relevance to humans of the triketone herbicide 2-(4-methylsulfonyl-2-nitrobenzoyl)-1,3-cyclohexanedione. Crit Rev Toxicol. 2013;43(3):185-99. https://doi.org/10.3109/10408444.2013.764279
84. Low K, Tasheva, M. Mesotrione. World Health Organization. 2014. Available from: https://apps.who.int/pesticide-residues-jmpr-database/Document/228
85. Brownsey RW, Zhande R, Boone AN. Isoforms of acetyl-CoA carboxylase: structures, regulatory properties and metabolic functions. Biochemical Society Transactions. 1997; 25 (4): 1232–1238. https://doi.org/10.1042/bst0251232

86. Tong L, Harwood Jr HJ. Acetyl-coenzyme A carboxylases: versatile targets for drug discovery. Journal of cellular biochemistry vol. 99,6 (2006): 1476-88. doi:10.1002/jcb.21077
87. Munday MR, Hemingway CJ. The regulation of acetyl-CoA carboxylase--a potential target for the action of hypolipidemic agents. Adv Enzyme Regul. 1999;39:205-34. https://doi.org/10.1016/s0065-2571(98)00016-8
88. Lee J, Walsh MC, Hoehn KL, James DE, Wherry EJ, Choi Y. Regulator of fatty acid metabolism, acetyl coenzyme a carboxylase 1, controls T cell immunity. J Immunol. 2014;192(7):3190-9. https://doi.org/10.4049/jimmunol.1302985
89. Yeudall S, Upchurch CM, Seegren PV, Pavelec CM, Greulich J, Lemke MC, Harris TE, et al. Macrophage acetyl-CoA carboxylase regulates acute inflammation through control of glucose and lipid metabolism. Sci Adv. 2022;8(47):eabq1984. https://doi.org/10.1126/sciadv.abq1984
90. Zu X, Zhong J, Luo D, Tan J, Zhang Q, Wu Y, Liu J, Cao R, Wen G, Cao D. Chemical genetics of acetyl-CoA carboxylases. Molecules. 2013;18(2):1704-1719. https://doi.org/10.3390/molecules18021704
91. Seng TW, Skillman TR, Yang N, Hammond C. Cyclohexanedione herbicides are inhibitors of rat heart acetyl-CoA carboxylase. Bioorg Med Chem Lett. 2003;13(19):3237-3242. https://doi.org/10.1016/s0960-894x(03)00664-4
92. Liu T, Gou L, Yan S, Huang T. Inhibition of acetyl-CoA carboxylase by PP-7a exerts beneficial effects on metabolic dysregulation in a mouse model of diet-induced obesity. Exp Ther Med. 2020;20(1):521-529. https://doi.org/10.3892/etm.2020.8700
93. Batchuluun B, Pinkosky SL, Steinberg GR. Lipogenesis inhibitors: therapeutic opportunities and challenges. Nat Rev Drug Discov. 2022;21(4):283-305. https://doi.org/10.1038/s41573-021-00367-2
94. Lochner M, Berod L, Sparwasser T. Fatty acid metabolism in the regulation of T cell function. Trends Immunol. 2015;36(2):81-91. https://doi.org/10.1016/j.it.2014.12.005
95. Byersdorfer CA. The role of Fatty Acid oxidation in the metabolic reprograming of activated t-cells. Front Immunol. 2014;5:641. https://doi.org/10.3389/fimmu.2014.00641
96. Zhang C, Yu Q, Han H, Yu C, Nyporko A, Tian X, Beckie H, Powles S. A naturally evolved mutation (Ser59Gly) in glutamine synthetase confers glufosinate resistance in plants. J Exp Bot. 2022;73(7):2251-2262. https://doi.org/10.1093/jxb/erac008
97. Krajewski WW, Collins R, Holmberg-Schiavone L, Jones TA, Karlberg T, Mowbray SL. Crystal structures of mammalian glutamine synthetases illustrate substrate-induced conformational changes and provide opportunities for drug and herbicide design. J Mol Biol. 2008;375(1):217-28. https://doi.org/10.1016/j.jmb.2007.10.029
98. Lane A.N., Fan T.W. Regulation of mammalian nucleotide metabolism and biosynthesis. Nucleic Acids Res. 2015;43:2466–2485. https://doi.org/10.1093/nar/gkv047
99. Albrecht J, Sidoryk-Wegrzynowicz M, Zielinska M, Aschner M. Roles of glutamine in neurotransmission. Neuron Glia Biol. 2010;6:263-276. https://doi.org/10.1017/S1740925X11000093
100. Newsholme P. Why is l-glutamine metabolism important to cells of the immune system in health, postinjury, surgery or infection? J. Nutr. 2001;131:2515S–2522S. https://doi.org/10.1093/jn/131.9.2515S
101. Spodenkiewicz M, Diez-Fernandez C, Rüfenacht V, Gemperle-Britschgi C, Häberle J. Minireview on Glutamine Synthetase Deficiency, an Ultra-Rare Inborn Error of Amino Acid Biosynthesis. Biology (Basel). 2016;5(4):40. https://doi.org/10.3390/biology5040040
102. Coyle JT, Puttfarcken P. Oxidative stress, glutamate, and neurodegenerative disorders. Science. 1993;262(5134):689-95. https://doi.org/10.1126/science.7901908

103. Neves D, Salazar IL, Almeida RD, Silva RM. Molecular mechanisms of ischemia and glutamate excitotoxicity. Life Sci. 2023;328:121814. https://doi.org/10.1016/j.lfs.2023.121814
104. Chávez-Castillo M, Rojas M, Bautista J. Excitotoxicity: an organized crime at the cellular level. J Neurol Neurosci. 2017;8(3):193.
105. Sulimenko V, Dráberová E, Dráber P. γ-Tubulin in microtubule nucleation and beyond. Front Cell Dev Biol. 2022;10:880761. https://doi.org/10.3389/fcell.2022.880761
106. Chen X, Wu S, Liu Z, Friml J. Environmental and endogenous control of cortical microtubule orientation. Trends Cell Biol. 2016;26(6):409-419.
107. Carmona B, Marinho HS, Matos CL, Nolasco S, Soares H. Tubulin Post-Translational Modifications: The Elusive Roles of Acetylation. Biology (Basel). 2023;12(4):561. https://doi.org/10.3390/biology12040561
108. Giglio A, Vommaro ML. Dinitroaniline herbicides: a comprehensive review of toxicity and side effects on animal non-target organisms. Environ Sci Pollut Res Int. 2022;29(51):76687-76711. https://doi.org/10.1007/s11356-022-23169-4
109. Ansari SM, Saquib Q, Attia SM, Abdel-Salam EM, Alwathnani HA, Faisal M, Alatar AA, Al-Khedhairy AA, Musarrat J. Pendimethalin induces oxidative stress, DNA damage, and mitochondrial dysfunction to trigger apoptosis in human lymphocytes and rat bone-marrow cells. Histochem Cell Biol. 2018;149(2):127-141. https://doi.org/10.1007/s00418-017-1622-0
110. Ribas G, Surrallés J, Carbonell E, Xamena N, Creus A, Marcos R. Genotoxic evaluation of the herbicide trifluralin on human lymphocytes exposed in vitro. Mutat Res. 1996;371(1-2):15-21. https://doi.org/10.1016/s0165-1218(96)90090-7
111. Sarıgöl Kılıç Z, Aydın S, Ündeğer Bucurgat Ü, Başaran N. In vitro genotoxicity assessment of pendimethalin and trifluralin. Food Chem Toxicol. 2018;113:90-98. https://doi.org/10.1016/j.fct.2018.01.034
112. Muehlebach ME, Holstein SA. Geranylgeranyl diphosphate synthase: Role in human health, disease and potential therapeutic target. Clin Transl Med. 2023;13(1):e1167. https://doi.org/10.1002/ctm2.1167
113. Kagami S, Owada T, Kanari H, Saito Y, Suto A, Ikeda K, Hirose K, Watanabe N, Iwamoto I, Nakajima H. Protein geranylgeranylation regulates the balance between Th17 cells and Foxp3+ regulatory T cells. Int Immunol. 2009;21(6):679-689. https://doi.org/10.1093/intimm/dxp037
114. Akula MK, Ibrahim MX, Ivarsson EG, Khan OM, Kumar IT, Erlandsson M, Karlsson C, Xu X, Brisslert M, Brakebusch C, Wang D, Bokarewa M, Sayin VI, Bergo MO. Protein prenylation restrains innate immunity by inhibiting Rac1 effector interactions. Nat Commun. 2019;10(1):3975. https://doi.org/10.1038/s41467-019-11606-x
115. Muehlebach ME, Holstein SA. Geranylgeranyl diphosphate synthase: Role in human health, disease and potential therapeutic target. Clin Transl Med. 2023;13(1):e1167. https://doi.org/10.1002/ctm2.1167
116. Marchwicka A, Kamińska D, Monirialamdari M, Błażewska KM, Gendaszewska-Darmach E. Protein Prenyltransferases and Their Inhibitors: Structural and Functional Characterization. International Journal of Molecular Sciences. 2022; 23(10):5424. https://doi.org/10.3390/ijms23105424
117. Yang SH, Chang SY, Tu Y, Lawson GW, Bergo MO, Fong LG, Young SG. Severe hepatocellular disease in mice lacking one or both CaaX prenyltransferases. J Lipid Res. 2012;53(1):77-86. https://doi.org/10.1194/jlr.M021220
118. Khan OM, Ibrahim MX, Jonsson IM, Karlsson C, Liu M, Sjogren AK, Olofsson FJ, Brisslert M, Andersson S, Ohlsson C, Hultén LM, Bokarewa M, Bergo MO.

Geranylgeranyltransferase type I (GGTase-I) deficiency hyperactivates macrophages and induces erosive arthritis in mice. J Clin Invest. 2011;121(2):628-39. https://doi.org/10.1172/JCI43758

119. Dailey TA, Dailey HA. Human protoporphyrinogen oxidase: expression, purification, and characterization of the cloned enzyme. Protein Sci. 1996;5(1):98-105. https://doi.org/10.1002/pro.5560050112

120. Hao GF, Zuo Y, Yang SG, Yang GF. Protoporphyrinogen oxidase inhibitor: an ideal target for herbicide discovery. Chimia (Aarau). 2011;65(12):961-969. https://doi.org/10.2533/chimia.2011.961

121. Matringe M, Camadro JM, Labbe P, Scalla, R. Protoporphyrinogen oxidase as a molecular target for diphenyl ether herbicides. Biochem. J. 260, 231–235.

122. Corrigall AV, Hift RJ, Adams PA, Kirsch RE. Inhibition of mammalian protoporphyrinogen oxidase by acifluorfen. Biochem Mol Biol Int. 1994;34(6):1283-9. PMID: 7697001.

123. Iwashita K, Hosokawa Y, Ihara R, Miyamoto T, Otani M, Abe J, Asano K, Mercier O, Miyata K, Barlow S. Flumioxazin, a PPO inhibitor: A weight-of-evidence consideration of its mode of action as a developmental toxicant in the rat and its relevance to humans. Toxicology. 2022;472:153160. https://doi.org/10.1016/j.tox.2022.153160

124. Byrnes SA, Weigl BH. Selecting analytical biomarkers for diagnostic applications: a first principles approach. Expert Rev Mol Diagn. 2018;18(1):19-26. https://doi.org/10.1080/14737159.2018.1412258

125. Jakubek M, Masařík M, Bříza T, Kaplánek R, Veselá K, Abramenko N, Martásek P. PPO-Inhibiting Herbicides and Structurally Relevant Schiff Bases: Evaluation of Inhibitory Activities against Human Protoporphyrinogen Oxidase. Processes. 2021; 9(2):383. https://doi.org/10.3390/pr9020383

126. Shepherd M, Dailey HA. A continuous fluorimetric assay for protoporphyrinogen oxidase by monitoring porphyrin accumulation. Anal Biochem. 2005;344(1):115-121. https://doi.org/10.1016/j.ab.2005.06.012

127. Cheng Z, Lu X, Feng B. A review of research progress of antitumor drugs based on tubulin targets. Transl Cancer Res. 2020;9(6):4020-4027. https://doi.org/10.21037/tcr-20-682

128. Tomoeda K, Awata H, Matsuura T, et al. Mutations in the 4-hydroxyphenylpyruvic acid dioxygenase gene are responsible for tyrosinemia type III and hawkinsinuria. Mol Genet Metab. 2000;71(3):506-510. https://doi.org/10.1006/mgme.2000.3085

129. Rüetschi U, Dellsén A, Sahlin P, Stenman G, Rymo L, Lindstedt S. Human 4-hydroxyphenylpyruvate dioxygenase. Primary structure and chromosomal localization of the gene. Eur J Biochem. 1993;213(3):1081-9. https://doi.org/10.1111/j.1432-1033.1993.tb17857.x

130. Hughes AT, Milan AM, Shweihdi E, Gallagher J, Ranganath L. Method development and validation for analysis of phenylalanine, 4-hydroxyphenyllactic acid and 4-hydroxyphenylpyruvic acid in serum and urine. JIMD Rep. 2022;63(4):341-350. https://doi.org/10.1002/jmd2.12287

131. Kennaway NG, Buist NR, Fellman JH. The origin of urinary p-hydroxyphenylpyruvate in a patient with hepatic cytosol tyrosine aminotransferase deficiency. Clin Chim Acta. 1972;41:157-61. https://doi.org/10.1016/0009-8981(72)90506-2

132. Iverson AJ, Bianchi A, Nordlund AC, Witters LA. Immunological analysis of acetyl-CoA carboxylase mass, tissue distribution and subunit composition. Biochem J. 1990;269(2):365-71. https://doi.org/10.1042/bj2690365

133. Castegna A, Menga A. Glutamine Synthetase: Localization Dictates Outcome. Genes (Basel). 2018;9(2):108. https://doi.org/10.3390/genes9020108

134. Takahashi M, Stanton E, Moreno JI, Jackowski G. Immunoassay for serum glutamine synthetase in serum: development, reference values, and preliminary study in dementias. Clin Chem. 2002;48(2):375-8.
135. Peng IC, Bott AJ, Zong WX. Spectrophotometric Determination of Glutamine Synthetase Activity in Cultured Cells. Bio Protoc. 2016;6(19):e1959. https://doi.org/10.21769/BioProtoc.1959
136. Sum CS, Nickischer D, Lei M, Weston A, Zhang L, Schweizer L. Establishing a High-content Analysis Method for Tubulin Polymerization to Evaluate Both the Stabilizing and Destabilizing Activities of Compounds. Curr Chem Genom Transl Med. 2014;8(Suppl 1):16-26. https://doi.org/10.2174/2213988501408010016
137. Laisne MC, Michallet S, Lafanechère L. Characterization of Microtubule Destabilizing Drugs: A Quantitative Cell-Based Assay That Bridges the Gap between Tubulin Based- and Cytotoxicity Assays. Cancers (Basel). 2021;13(20):5226. https://doi.org/10.3390/cancers13205226
138. Gaskin F. Analysis of microtubule assembly kinetics using turbidimetry. Methods Mol Biol. 2011;777:99-105. https://doi.org/10.1007/978-1-61779-252-6_7
139. Mirigian M, Mukherjee K, Bane SL, Sackett DL. Measurement of in vitro microtubule polymerization by turbidity and fluorescence. Methods Cell Biol. 2013;115:215-229. https://doi.org/10.1016/B978-0-12-407757-7.00014-1
140. Chhonker YS, Haney SL, Bala V, Holstein SA, Murry DJ. Simultaneous Quantitation of Isoprenoid Pyrophosphates in Plasma and Cancer Cells Using LC-MS/MS. Molecules. 2018;23(12):3275. https://doi.org/10.3390/molecules23123275
141. Holstein SA, Tong H, Kuder CH, Hohl RJ. Quantitative determination of geranyl diphosphate levels in cultured human cells. Lipids. 2009;44(11):1055-62. https://doi.org/10.1007/s11745-009-3355-x
142. Tong H, Wiemer AJ, Neighbors JD, Hohl RJ. Quantitative determination of farnesyl and geranylgeranyl diphosphate levels in mammalian tissue. Anal Biochem. 2008;378(2):138-43. https://doi.org/10.1016/j.ab.2008.04.021
143. Henneman L, van Cruchten AG, Denis SW, et al. Detection of nonsterol isoprenoids by HPLC-MS/MS. Anal Biochem. 2008;383(1):18-24. https://doi.org/10.1016/j.ab.2008.08.023
144. Jacobs NJ, Jacobs JM. Assay for enzymatic protoporphyrinogen oxidation, a late step in heme synthesis. Enzyme. 1982;28(2-3):206-19. doi: 10.1159/000459103.
145. Shepherd M, Dailey HA. A continuous fluorimetric assay for protoporphyrinogen oxidase by monitoring porphyrin accumulation. Anal Biochem. 2005;344(1):115-21. https://doi.org/10.1016/j.ab.2005.06.012
146. Lerro CC, Beane Freeman LE, Portengen L, Kang D, Lee K, Blair A, Lynch CF, Bakke B, De Roos AJ, Vermeulen RC. A longitudinal study of atrazine and 2,4-D exposure and oxidative stress markers among iowa corn farmers. Environ Mol Mutagen. 2017;58(1):30-38. doi: 10.1002/em.22069.
147. Hilgert Jacobsen-Pereira C, Dos Santos CR, Troina Maraslis F, Pimentel L, Feijó AJL, Iomara Silva C, de Medeiros GDS, Costa Zeferino R, Curi Pedrosa R, Weidner Maluf S. Markers of genotoxicity and oxidative stress in farmers exposed to pesticides. Ecotoxicol Environ Saf. 2018;148:177-183. https://doi.org/10.1016/j.ecoenv.2017.10.004
148. Zanchi MM, Marafon F, Marins K, Bagatini MD, Zamoner A. Redox imbalance and inflammation: A link to depression risk in brazilian pesticide-exposed farmers. Toxicology. 2024;501:153706. https://doi.org/10.1016/j.tox.2023.153706

149. Sidthilaw S, Sapbamrer R, Pothirat C, Wunnapuk K, Khacha-Ananda S. Effects of exposure to glyphosate on oxidative stress, inflammation, and lung function in maize farmers, Northern Thailand. BMC Public Health. 2022;22(1):1343. https://doi.org/10.1186/s12889-022-13696-7
150. Chang VC, Andreotti G, Ospina M, Parks CG, Liu D, Shearer JJ, Rothman N, Silverman DT, Sandler DP, Calafat AM, Beane Freeman LE, Hofmann JN. Glyphosate exposure and urinary oxidative stress biomarkers in the Agricultural Health Study. J Natl Cancer Inst. 2023;115(4):394-404. https://doi.org/10.1093/jnci/djac242
151. Ryan PB, Burke TA, Cohen Hubal EA, Cura JJ, McKone TE. Using biomarkers to inform cumulative risk assessment. Environ Health Perspect. 2007;115(5):833-40. https://doi.org/10.1289/ehp.9334
152. Norén E, Lindh C, Rylander L, Glynn A, Axelsson J, Littorin M, Faniband M, Larsson E, Nielsen C. Concentrations and temporal trends in pesticide biomarkers in urine of Swedish adolescents, 2000-2017. J Expo Sci Environ Epidemiol. 2020;30(4):756-767. https://doi.org/10.1038/s41370-020-0212-8
153. Anwar WA. Biomarkers of human exposure to pesticides. Environ Health Perspect. 1997;105 Suppl 4(Suppl 4):801-6. https://doi.org/10.1289/ehp.97105s4801
154. McClure GY, Freeman JP, Lay JO, Hinson JA. Haemoglobin adducts as biomarkers of exposure to the herbicides propanil and fluometuron. Biomarkers. 1996;1(2):136-40. https://doi.org/10.3109/13547509609088681
155. Dooley GP, Prenni JE, Prentiss PL, Cranmer BK, Andersen ME, Tessari JD. Identification of a novel hemoglobin adduct in Sprague Dawley rats exposed to atrazine. Chem Res Toxicol. 2006;19(5):692-700. https://doi.org/10.1021/tx060023c
156. Chu S, Letcher RJ. Bottom-up proteomics analysis for adduction of the broad-spectrum herbicide atrazine to histone. Anal Bioanal Chem. 2023;415(8):1497-1504. https://doi.org/10.1007/s00216-023-04545-6
157. Jabłońska-Trypuć A, Wołejko E, Wydro U, Butarewicz A. The impact of pesticides on oxidative stress level in human organism and their activity as an endocrine disruptor. J Environ Sci Health B. 2017;52(7):483-494. https://doi.org/10.1080/03601234.2017.1303322
158. Sule RO, Condon L, Gomes AV. A Common Feature of Pesticides: Oxidative Stress-The Role of Oxidative Stress in Pesticide-Induced Toxicity. Oxid Med Cell Longev. 2022;2022:5563759. https://doi.org/10.1155/2022/5563759
159. Lopes-Ferreira M, Farinha LRL, Costa YSO, Pinto FJ, Disner GR, da Rosa JGDS, Lima C. Pesticide-Induced Inflammation at a Glance. Toxics. 2023;11(11):896. https://doi.org/10.3390/toxics11110896
160. Bolognesi C. Genotoxicity of pesticides: a review of human biomonitoring studies. Mutat Res. 2003;543(3):251-72. https://doi.org/10.1016/s1383-5742(03)00015-2
161. Mokarizadeh A, Faryabi MR, Rezvanfar MA, Abdollahi M. A comprehensive review of pesticides and the immune dysregulation: mechanisms, evidence and consequences. Toxicol Mech Methods. 2015;25(4):258-78. https://doi.org/10.3109/15376516.2015.1020182
162. Mostafalou S, Abdollahi M. Pesticides and human chronic diseases: evidences, mechanisms, and perspectives. Toxicol Appl Pharmacol. 2013;268(2):157-77. doi: 10.1016/j.taap.2013.01.025
163. Hubler MJ, Kennedy AJ. Role of lipids in the metabolism and activation of immune cells. J Nutr Biochem. 2016;34:1-7. https://doi.org/10.1016/j.jnutbio.2015.11.002
164. Yeudall S, Upchurch CM, Seegren PV, Pavelec CM, Greulich J, Lemke MC, Harris TE, Desai BN, Hoehn KL, Leitinger N. Macrophage acetyl-CoA carboxylase regulates acute

inflammation through control of glucose and lipid metabolism. Sci Adv. 2022;8(47):eabq1984. https://doi.org/10.1126/sciadv.abq1984.

165. Lee J, Walsh MC, Hoehn KL, James DE, Wherry EJ, Choi Y. Regulator of fatty acid metabolism, acetyl coenzyme a carboxylase 1, controls T cell immunity. J Immunol. 2014;192(7):3190-9. https://doi.org/10.4049/jimmunol.1302985

166. Kagami S, Owada T, Kanari H, Saito Y, Suto A, Ikeda K, Hirose K, Watanabe N, Iwamoto I, Nakajima H. Protein geranylgeranylation regulates the balance between Th17 cells and Foxp3+ regulatory T cells. Int Immunol. 2009;21(6):679-89. https://doi.org/10.1093/intimm/dxp037

167. Akula MK, Ibrahim MX, Ivarsson EG, Khan OM, Kumar IT, Erlandsson M, Karlsson C, Xu X, Brisslert M, Brakebusch C, Wang D, Bokarewa M, Sayin VI, Bergo MO. Protein prenylation restrains innate immunity by inhibiting Rac1 effector interactions. Nat Commun. 2019;10(1):3975. https://doi.org/10.1038/s41467-019-11606-x

168. Bibby JA, Purvis HA, Hayday T, Chandra A, Okkenhaug K, Rosenzweig S, Aksentijevich I, Wood M, Lachmann HJ, Kemper C, Cope AP, Perucha E. Cholesterol metabolism drives regulatory B cell IL-10 through provision of geranylgeranyl pyrophosphate. Nat Commun. 2020;11(1):3412. https://doi.org/10.1038/s41467-020-17179-4

169. Dunn SE, Youssef S, Goldstein MJ, Prod'homme T, Weber MS, Zamvil SS, Steinman L. Isoprenoids determine Th1/Th2 fate in pathogenic T cells, providing a mechanism of modulation of autoimmunity by atorvastatin. J Exp Med. 2006;203(2):401-12. https://doi.org/10.1084/jem.20051129

170. Teodoro M, Briguglio G, Fenga C, Costa C. Genetic polymorphisms as determinants of pesticide toxicity: Recent advances. Toxicol Rep. 2019;6:564-570. https://doi.org/10.1016/j.toxrep.2019.06.004

171. Cao L, Kang Q, Tian Y. Pesticide residues: Bridging the gap between environmental exposure and chronic disease through omics. Ecotoxicol Environ Saf. 2024;287:117335. https://doi.org/10.1016/j.ecoenv.2024.117335

172. Bradberry SM, Watt BE, Proudfoot AT, Vale JA. Mechanisms of toxicity, clinical features, and management of acute chlorophenoxy herbicide poisoning: a review. J Toxicol Clin Toxicol. 2000;38(2):111-22. https://doi.org/10.1081/clt-100100925

173. Bradberry SM, Proudfoot AT, Vale JA. Poisoning due to chlorophenoxy herbicides. Toxicol Rev. 2004;23(2):65-73. https://doi.org/10.2165/00139709-200423020-00001

174. Bradberry SM, Proudfoot AT, Vale JA. Glyphosate poisoning. Toxicol Rev. 2004;23(3):159-67. https://doi.org/10.2165/00139709-200423030-00003

175. Lo YC, Yang CC, Deng JF. Acute alachlor and butachlor herbicide poisoning. Clin Toxicol (Phila). 2008;46(8):716-21. doi: 10.1080/15563650701704834

176. Mohd Ghazi R, Nik Yusoff NR, Abdul Halim NS, Wahab IRA, Ab Latif N, Hasmoni SH, Ahmad Zaini MA, Zakaria ZA. Health effects of herbicides and its current removal strategies. Bioengineered. 2023;14(1):2259526. https://doi.org/10.1080/21655979.2023.2259526

177. Lindell AE, Zimmermann-Kogadeeva M, Patil KR. Multimodal interactions of drugs, natural compounds and pollutants with the gut microbiota. Nat Rev Microbiol. 2022;20(7):431-443. doi: 10.1038/s41579-022-00681-5.

178. Mesnage R, Teixeira M, Mandrioli D, Falcioni L, Ducarmon QR, Zwittink RD, Mazzacuva F, Caldwell A, Halket J, Amiel C, Panoff JM, Belpoggi F, Antoniou MN. Use of Shotgun Metagenomics and Metabolomics to Evaluate the Impact of Glyphosate or Roundup MON 52276 on the Gut Microbiota and Serum Metabolome of Sprague-Dawley Rats. Environ Health Perspect. 2021;129(1):17005. doi: 10.1289/EHP6990.

179. Kulcsarova K, Bang C, Berg D, Schaeffer E. Pesticides and the Microbiome-Gut-Brain Axis: Convergent Pathways in the Pathogenesis of Parkinson's Disease. J Parkinsons Dis. 2023;13(7):1079-1106. doi: 10.3233/JPD-230206.

180. Ali A, AlHussaini KI. Pesticides: Unintended Impact on the Hidden World of Gut Microbiota. Metabolites. 2024;14(3):155. doi: 10.3390/metabo14030155.

181. Sharma T, Sirpu Natesh N, Pothuraju R, Batra SK, Rachagani S. Gut microbiota: a non-target victim of pesticide-induced toxicity. Gut Microbes. 2023;15(1):2187578. doi: 10.1080/19490976.2023.2187578.

182. Smith AC, Cronan JE. Dimerization of the bacterial biotin carboxylase subunit is required for acetyl coenzyme A carboxylase activity in vivo. J Bacteriol. 2012;194(1):72-8. doi: 10.1128/JB.06309-11.

183. Gancedo C, Holzer H. Enzymatic inactivation of glutamine synthetase in Enterobacteriaceae. Eur J Biochem. 1968;4(2):190-2. doi: 10.1111/j.1432-1033.1968.tb00192.x.

184. Schmaler-Ripcke J, Sugareva V, Gebhardt P, Winkler R, Kniemeyer O, Heinekamp T, Brakhage AA. Production of pyomelanin, a second type of melanin, via the tyrosine degradation pathway in Aspergillus fumigatus. Appl Environ Microbiol. 2009;75(2):493-503. doi: 10.1128/AEM.02077-08.

185. Ueyama J, Hayashi M, Hirayama M, Nishiwaki H, Ito M, Saito I, Tsuboi Y, Isobe T, Ohno K. Effects of Pesticide Intake on Gut Microbiota and Metabolites in Healthy Adults. Int J Environ Res Public Health. 2022 Dec 23;20(1):213. doi: 10.3390/ijerph20010213.

186. Pue N, Guddat LW. Acetohydroxyacid synthase: a target for antimicrobial drug discovery. Curr Pharm Des. 2014;20(5):740-53. doi: 10.2174/13816128113199990009.

187. Steinrücken, HC, Amrhein, N. The glyphosate target enzyme 5-enolpyruvylshikimate 3-phosphate synthase (EPSPS) contains several EPSPS-associated domains in fungi. Biochem Biophys Res Commun. 1984; 122(3), 1047-1053. doi: 10.1016/0006-291X(84)91193-6.

188. Del Castilo I, Neumann AS, Lemos FS, De Bastiani MA, Oliveira FL, Zimmer ER, Rêgo AM, Hardoim CCP, Antunes LCM, Lara FA, Figueiredo CP, Clarke JR. Lifelong Exposure to a Low-Dose of the Glyphosate-Based Herbicide RoundUp Causes Intestinal Damage, Gut Dysbiosis, and Behavioral Changes in Mice. Int J Mol Sci. 2022;23(10):5583. https://doi.org/10.3390/ijms23105583

189. Matsuzaki R, Gunnigle E, Geissen V, Clarke G, Nagpal J, Cryan JF. Pesticide exposure and the microbiota-gut-brain axis. ISME J. 2023;17(8):1153-1166. https://doi.org/10.1038/s41396-023-01450-9

Click here to access/download

Supplemental Material

EHP Seminar Proposal Letter_EHP16005R1.pdf

Environmental Health Perspectives

Biologia compartilhada planta-humana: efeitos de herbicidas e novas perspectivas de biomarcadores

--Manuscript Draft--

**Manuscript Number:**	EHP16005R1
**Full Title:**	Biologia compartilhada planta-humana: efeitos de herbicidas e novas perspectivas de biomarcadores
**Article Type:**	Seminar Proposal
**Section/Category:**	Toxicology
**Corresponding Author:**	Aline de Souza Espindola, Ph.D., MPH<br>Universidade Federal do Rio de Janeiro<br>Rio de Janeiro, RJ BRAZIL
**Corresponding Author Secondary Information:**	
**Corresponding Author's Institution:**	Universidade Federal do Rio de Janeiro
**First Author:**	Aline de Souza Espindola, Ph.D., MPH
**First Author Secondary Information:**	
**Order of Authors:**	Aline de Souza Espindola, Ph.D., MPH
	Isabella Gaichi Giannini Romaguera, Graduate student
	Christine Gibson Parks, MSPH, PhD
	Josino Costa Moreira, MSc, PhD
**Order of Authors Secondary Information:**	
**Manuscript Region of Origin:**	BRAZIL
**Additional Information:**	
**Question**	**Response**
**Funding Information:**	
**Manuscript Classifications:**	10.020: Epidemiology; 10.060: Toxicology; 10.070: Other Discipline; 30.230: Pesticides
**Author Comments:**	Joel D. Kaufman, MD, MPH<br>Editor-in-Chief<br>Environmental Health Perspectives<br><br>August 14, 2024<br><br>Dear Editor,<br><br>I wish to submit a seminar manuscript for publication in Environmental Health Perspectives, titled “Shared plant-human biology: Herbicide effects on humans and new biomarkers perspectives”.<br><br>Herbicide exposure has been linked to both acute poisonings and chronic diseases, yet the mechanisms underlying herbicide effects in humans remain unknown. Notably, humans and plants share biological processes that are targeted by herbicides. This seminar aims to explore these shared processes to identify potential molecular targets of herbicide exposure in humans, as well as biomarkers of effect. The results of the seminar highlighted five potential molecular targets, including tubulin levels and key enzymes crucial for metabolic pathways, such as hydroxyphenylpyruvate dioxygenase, coenzyme A carboxylase, glutamine synthetase, and protoporphyrinogen oxidase. Protein targets, including tubulin and the enzymes above, can be quantified in biological samples through established techniques. Inhibition of these targets is uncommon and may arise from some factors such as genetic mutations, exposure to

specific chemicals or medications, metabolic disorders, and rare diseases. As a result, these molecular targets may serve as promising candidates for biomarkers of herbicide-induced effects in humans.

This seminar highlights the importance of comprehensive research efforts to understand the herbicide's effects on humans from the perspective of effect biomarkers. In an era where advanced omics technologies and biostatistics aim to comprehend the detailed mechanisms of action on how pesticides interact with the organism, the use of traditional biomarkers, such as those for effects, may demarcate exposure precede adverse effects. Further, we believe that this manuscript will be of interest to the readership of your journal because it expands our understanding of how herbicides interact with human biology, shedding light on little-explored mechanisms.

This manuscript has not been published or presented elsewhere in part or entirety and is not under consideration by another journal. We have read and understood your journal's policies, and we believe that neither the manuscript nor the study violates any of these. There are no conflicts of interest to declare.

Thank you for your consideration. I look forward to hearing from you.

Sincerely,
Aline de Souza Espindola
Associate Professor of Occupational and Environmental Health
Public Health Institute
Federal University of Rio de Janeiro
ORCID: https://orcid.org/0000-0002-5498-3992

Shared plant-human biology: herbicide effects and new biomarkers perspectives - Responses to Reviewers' Comments

Dear reviewers,
We appreciate all the constructive feedback that has helped improve the manuscript. All responses in this document and revisions in the manuscript have been highlighted in red to facilitate review and readability.

Reviewer 1:
-In the seminar proposal entitled, Shared plant-human biology: herbicide effects and new biomarkers perspectives, I found the topic interesting, and with some adjustments, the seminar could be compelling.
Response: Thank you very much. I am genuinely pleased that the reviewer found the proposal interesting and valuable, and I appreciate the opportunity to discuss its refinement.
-In the author line, the authors declare their academic ranks for postdocs, grads, and undergrads. It is appreciated that the authors are including trainees in their seminar (although not necessary to include in the full seminar).
Response: Thank you for your suggestion. The trainee was responsible for gathering relevant references on "herbicide classes and their action mechanisms in plants", particularly from PubMed and ScienceDirect databases, using Advanced Search techniques under my direct supervision. Since the trainee did not participate directly in developing the proposal, we will mention their support in the acknowledgments section.
-The summary on diversifying the topics in environmental health is somewhat limited. One might expect that the authors would highlight some of the broader toxicity themes associated with herbicide exposure (i.e., inflammation, oxidative stress, etc.) to provide a deeper connection between the human and plant mechanisms and how their perspectives significantly advance the field.
Response: We agree with the suggestion and have added the following to the tracking version (lines 84-85): Identifying molecular targets of herbicides in humans and discussing their underlying and broader toxicity mechanisms, which include oxidative stress, inflammation, and immunotoxicity, could also provide insight into the long-term effects of these chemicals.

-The authors make sweeping statements at times without a thorough explanation. "Herbicide exposure has been associated with acute poisonings and chronic diseases."

Powered by Editorial Manager® and ProduXion Manager® from Aries Systems Corporation

	Which chronic diseases?<br>Response: We agree that the statement is vague and lacks precision. We have added the following (lines 104-105):Background: Herbicide exposure has been associated with acute poisonings and chronic diseases such as autoimmune diseases, cancer, neurodegenerative disorders, and renal diseases.<br><br>-The overall focus tends to favor enzymes and the inhibitory mechanisms of the herbicide; however physiological mechanisms of toxicity and their impacts are not mentioned in detail.<br>Response: We have included potential physiopathological mechanisms of herbicides based on evidence from in vitro, in vivo, and human studies when available. When experimental or human data are lacking, we provide information on the toxic mechanisms (adverse effects) of drugs that inhibit these enzymes, as they may be similar to herbicide effects. We have added the following to the tracking version:<br><br>2.2.1 HPPD (lines 237-239)<br><br>The accumulation of tyrosine and its metabolites in mammals is associated with reduced enzymatic and non-enzymatic antioxidant defenses and oxidative stress, resulting in toxicity in the eyes, liver, kidneys, and nervous system.43,44,45,46<br><br>2.2.2 ACC (lines 250-255)<br><br>Evidence for the effects of ACC inhibition by herbicides on mammals, either in vivo or in vivo, is currently lacking. However, inhibiting ACC with lipogenesis inhibitor drugs increases plasma triglyceride and very low-density lipoprotein (VLDL) levels, contributing to insulin resistance in both rodents and humans.50,51 This inhibition can also impair T-cell expansion and alter the differentiation of regulatory T-cells, both critical for maintaining immune balance and response to infections in mammals.52,53<br><br>2.2.3 GS (lines 262-266)<br><br>Glutamine is a precursor for the base of nucleic acids, neurotransmitters, and immune function regulation.56,57,58 GS inhibition in a rare disease reduces glutamine levels and can lead to hyperammonemia,59 impair immune cell activation, and disrupt neurotransmitter levels, contributing to neurological disorders, and immune dysregulation.59<br><br>2.2.4Tubulin inhibitors (lines 274-278)<br><br>Tubulin inhibitors can cause mitochondrial dysfunction, oxidative stress, genotoxicity, and cytotoxicity in mammals.62 In vitro studies with human lymphocytes and mammal cells exposed to tubulin inhibitors have shown increased sister-chromatid exchanges, chromosomal aberrations, and micronuclei.63,64<br><br>2.2.5 Metabolites of isoprenoid synthesis (lines 293-297)<br><br>However, their potential toxic effects in humans include disrupting the synthesis of isoprenoids, prenylated proteins, and biomolecules involved in cell signaling, and apoptosis.67,68 GGPP pathway enzyme deficiency in mammals has been associated with liver pathologies and severe joint inflammation in mice.69,70<br><br>2.2.6 PPO Inhibitors (305-308)<br><br>PPO inhibition leads to the accumulation of protoporphyrinogen IX, which interferes with mitochondrial function, impairs energy production, elevates ROS levels, and leads to intermediate filament protein aggregation in mammals.73,74<br><br>-I am not sure if the seminar can be considered comprehensive, as the authors suggest, but rather an enzymatically focused seminar.<br>Response: We agree with the observation that our research emphasizes enzymatic mechanisms. Accordingly, we have added the following (line 120): This seminar highlights the importance of an in-depth research effort on enzymatic pathways to understand herbicide effects on humans from the perspective of biomarkers as a tool

Powered by Editorial Manager® and ProduXion Manager® from Aries Systems Corporation

for early detection of biological alterations before adverse events or diseases develop.

-The authors cover many chemical enzyme pairs for plants, but only ~5 for humans. This is where applying physiological impacts of the other herbicides could help advance the theme of shared mechanisms between plants and humans.
Response: Yes, we agree with the statement and have added a specific topic on the physiological impacts of other herbicides, including additional mechanisms of action previously suggested in the last section of the proposal, as follows (lines 348-350):
- Understanding the nonspecific mechanisms of herbicides, including oxidative stress, inflammation, and microbiome alterations95,96 is crucial for guiding the development of herbicides that pose less risk to human health.

-The end biomarkers section is a bit limited. The authors may consider expanding this discussion to metabolites of the herbicides, changes in enzymatic flux, and adducts from the herbicides in addition to the broadly mentions biomarkers in the section.
Response: We added the following topics (lines 319-350):
◦ The importance of integrating exposure and effect biomarkers towards a more comprehensive understanding of how herbicides impact human health:
-The presence of herbicide metabolites in biological samples may indicate recent or cumulative herbicide exposure.80,81
-Herbicide adducts, formed by the covalent binding of herbicide molecules or their reactive metabolites to biological macromolecules, can serve as biomarkers of exposure. Depending on the type of biomacromolecule involved, they can indicate either recent or past exposure.82-85
-Herbicide exposure biomarkers can provide clues about the toxicological responses triggered by exposure, including oxidative stress,86,87 inflammation,88 genotoxicity,89 and immune response disturbances90 directly or indirectly associated with the proposed effect biomarkers.
•Certain herbicides specifically target plant processes, such as auxin receptors, VLCFAE enzymes, and unique biochemical pathways, which are absent in humans. However, these compounds exhibit acute toxicity in humans, and their long-term effects remain under investigation.95
•Understanding the nonspecific mechanisms of herbicides, including oxidative stress, inflammation, and microbiome alterations96,97 is crucial for guiding the development of herbicides that pose less risk to human health.

Overall, this is an interesting approach. I think it needs some fine tuning with regards to the mechanisms and biomarkers if selected for a seminar.

Reviewer 2:

This seminar article proposal is focused on overlapping plant and human biology important in herbicide toxicity. There are several strengths. The authorship team has all the required expertise, adding diversity across disciplines to the field. Herbicide use is persistent and, in some areas, increasing. Herbicides have been linked to many adverse health effects in humans. The focus on shared biological responses to herbicides in plants and humans may highlight overlapping efficacy (desired plant toxicity) and adverse outcomes (undesired toxicity in humans). Specific metabolic pathways are mentioned. There is a need for such an article and full submission development is supported.
Response: I am grateful for the support received for the submitted proposal and am pleased to know that it is of interest for a subsequent full submission.

In developing a full submission, there are some key suggestions:

1)Section 2.3 is superficially developed relative to other sections. The biomarker paragraphs should be far more detailed than the outline indicates, addressing biomarkers of exposure and toxicity.

Response: We added the following topics (lines 319-339):

◦ The importance of integrating exposure and effect biomarkers towards a more comprehensive understanding of how herbicides impact human health:
-The presence of herbicide metabolites in biological samples may indicate recent or

	cumulative herbicide exposure.80,81<br>-Herbicide adducts, formed by the covalent binding of herbicide molecules or their reactive metabolites to biological macromolecules, can serve as biomarkers of exposure. Depending on the type of biomacromolecule involved, they can indicate either recent or past exposure.82-85<br>-Herbicide exposure biomarkers can provide clues about the toxicological responses triggered by exposure, including oxidative stress,86,87 inflammation,88 genotoxicity,89 and immune response disturbances90 directly or indirectly associated with the effects of herbicides on proposed effect biomarkers.<br><br>Furthermore, in the previous sections, we included the potential toxicity mechanisms associated with the impact of herbicides on the proposed effect biomarkers, as requested by the previous reviewer, which allows for a link to the final section of the article.<br><br><br>2)While the focus is on shared biology, specifically shared biological responses to herbicides, a key takeaway could be expanded focus on mechanisms that do not have shared biology. The authors should address how what is learned from their critical analysis could be used to developing herbicides that do not target human biology at sufficient magnitude to impact health.<br>Response: We added the following topics (lines 344-350):<br>•Certain herbicides specifically target plant processes, such as auxin receptors, VLCFAE enzymes, and unique biochemical pathways, which are absent in humans. However, these compounds exhibit acute toxicity in humans, and their long-term effects remain under investigation.95<br>•Understanding the nonspecific mechanisms of herbicides, including oxidative stress, inflammation, and microbiome alterations96,97 is crucial for guiding the development of herbicides that pose less risk to human health.

Powered by Editorial Manager® and ProduXion Manager® from Aries Systems Corporation

Environmental Health Perspectives

Shared plant-human biology: herbicide effects and new biomarkers perspectives

Responses to Reviewers' Comments

Dear reviewers,

We appreciate all the constructive feedback that has helped improve the manuscript. All responses in this document and revisions in the manuscript have been highlighted in red to facilitate review and readability.

Reviewer 1:

- In the seminar proposal entitled, Shared plant-human biology: herbicide effects and new biomarkers perspectives, I found the topic interesting, and with some adjustments, the seminar could be compelling.

Response: Thank you very much. I am genuinely pleased that the reviewer found the proposal interesting and valuable, and I appreciate the opportunity to discuss its refinement.

- In the author line, the authors declare their academic ranks for postdocs, grads, and undergrads. It is appreciated that the authors are including trainees in their seminar (although not necessary to include in the full seminar).

Response: Thank you for your suggestion. The trainee was responsible for gathering relevant references on "herbicide classes and their action mechanisms in plants", particularly from PubMed and ScienceDirect databases, using Advanced Search techniques under my direct supervision. Since the trainee did not participate directly in developing the proposal, we will mention their support in the acknowledgments section.

- The summary on diversifying the topics in environmental health is somewhat limited. One might expect that the authors would highlight some of the broader toxicity themes associated with herbicide exposure (i.e., inflammation, oxidative stress, etc.) to provide a deeper connection between the human and plant mechanisms and how their perspectives significantly advance the field.

Response: We agree with the suggestion and have added the following to the tracking version (lines 84-85): Identifying molecular targets of herbicides in

humans and discussing their underlying and broader toxicity mechanisms, which include oxidative stress, inflammation, and immunotoxicity, could also provide insight into the long-term effects of these chemicals.

- The authors make sweeping statements at times without a thorough explanation. "Herbicide exposure has been associated with acute poisonings and chronic diseases." Which chronic diseases?

Response: We agree that the statement is vague and lacks precision. We have added the following (lines 104-105):**Background:** Herbicide exposure has been associated with acute poisonings and chronic diseases such as autoimmune diseases, cancer, neurodegenerative disorders, and renal diseases.

- The overall focus tends to favor enzymes and the inhibitory mechanisms of the herbicide; however physiological mechanisms of toxicity and their impacts are not mentioned in detail.

Response: We have included potential physiopathological mechanisms of herbicides based on evidence from in vitro, in vivo, and human studies when available. When experimental or human data are lacking, we provide information on the toxic mechanisms (adverse effects) of drugs that inhibit these enzymes, as they may be similar to herbicide effects. We have added the following to the tracking version:

2.2.1 HPPD (lines 237-239)

The accumulation of tyrosine and its metabolites in mammals is associated with reduced enzymatic and non-enzymatic antioxidant defenses and oxidative stress, resulting in toxicity in the eyes, liver, kidneys, and nervous system.[43,44,45,46]

2.2.2 ACC (lines 250-255)

Evidence for the effects of ACC inhibition by herbicides on mammals, either in vivo or in vivo, is currently lacking. However, inhibiting ACC with lipogenesis inhibitor drugs increases plasma triglyceride and very low-density lipoprotein (VLDL) levels, contributing to insulin resistance in both rodents and humans.[50,51] This inhibition can also impair T-cell expansion and alter the differentiation of regulatory T-cells, both critical for maintaining immune balance and response to infections in mammals.[52,53]

2.2.3 GS (lines 262-266)

Glutamine is a precursor for the base of nucleic acids, neurotransmitters, and immune function regulation.[56,57,58] GS inhibition in a rare disease reduces glutamine levels and can lead to hyperammonemia,[59] impair immune cell activation, and disrupt neurotransmitter levels, contributing to neurological disorders, and immune dysregulation.[59]

2.2.4 Tubulin inhibitors (lines 274-278)

Tubulin inhibitors can cause mitochondrial dysfunction, oxidative stress, genotoxicity, and cytotoxicity in mammals.[62] In vitro studies with human lymphocytes and mammal cells exposed to tubulin inhibitors have shown increased sister-chromatid exchanges, chromosomal aberrations, and micronuclei.[63,64]

2.2.5 Metabolites of isoprenoid synthesis (lines 293-297)

However, their potential toxic effects in humans include disrupting the synthesis of isoprenoids, prenylated proteins, and biomolecules involved in cell signaling, and apoptosis.[67,68] GGPP pathway enzyme deficiency in mammals has been associated with liver pathologies and severe joint inflammation in mice.[69,70]

2.2.6 PPO Inhibitors (305-308)

PPO inhibition leads to the accumulation of protoporphyrinogen IX, which interferes with mitochondrial function, impairs energy production, elevates ROS levels, and leads to intermediate filament protein aggregation in mammals.[73,74]

- I am not sure if the seminar can be considered comprehensive, as the authors suggest, but rather an enzymatically focused seminar.

Response: We agree with the observation that our research emphasizes enzymatic mechanisms. Accordingly, we have added the following (line 120): This seminar highlights the importance of <u>an in-depth research effort on enzymatic pathways</u> to understand herbicide effects on humans from the perspective of biomarkers as a tool for early detection of biological alterations before adverse events or diseases develop.

- The authors cover many chemical enzyme pairs for plants, but only ~5 for humans. This is where applying physiological impacts of the other herbicides could help advance the theme of shared mechanisms between plants and humans.

Response: Yes, we agree with the statement and have added a specific topic on the physiological impacts of other herbicides, including additional mechanisms of

action previously suggested in the last section of the proposal, as follows (lines 348-350):

- Understanding the nonspecific mechanisms of herbicides, including oxidative stress, inflammation, and microbiome alterations[95,96] is crucial for guiding the development of herbicides that pose less risk to human health.

- The end biomarkers section is a bit limited. The authors may consider expanding this discussion to metabolites of the herbicides, changes in enzymatic flux, and adducts from the herbicides in addition to the broadly mentions biomarkers in the section.

Response: We added the following topics (lines 319-350):

◦ The importance of integrating exposure and effect biomarkers towards a more comprehensive understanding of how herbicides impact human health:

- The presence of herbicide metabolites in biological samples may indicate recent or cumulative herbicide exposure.[80,81]
- Herbicide adducts, formed by the covalent binding of herbicide molecules or their reactive metabolites to biological macromolecules, can serve as biomarkers of exposure. Depending on the type of biomacromolecule involved, they can indicate either recent or past exposure.[82-85]
- Herbicide exposure biomarkers can provide clues about the toxicological responses triggered by exposure, including oxidative stress,[86,87] inflammation,[88] genotoxicity,[89] and immune response disturbances[90] directly or indirectly associated with the proposed effect biomarkers.

- Certain herbicides specifically target plant processes, such as auxin receptors, VLCFAE enzymes, and unique biochemical pathways, which are absent in humans. However, these compounds exhibit acute toxicity in humans, and their long-term effects remain under investigation.[95]
- Understanding the nonspecific mechanisms of herbicides, including oxidative stress, inflammation, and microbiome alterations[96,97] is crucial for guiding the development of herbicides that pose less risk to human health.

Overall, this is an interesting approach. I think it needs some fine tuning with regards to the mechanisms and biomarkers if selected for a seminar.

Reviewer 2:

This seminar article proposal is focused on overlapping plant and human biology important in herbicide toxicity. There are several strengths. The authorship team has all the required expertise, adding diversity across disciplines to the field. Herbicide use is persistent and, in some areas, increasing. Herbicides have been linked to many adverse health effects in humans. The focus on shared biological responses to herbicides in plants and humans may highlight overlapping efficacy (desired plant toxicity) and adverse outcomes (undesired toxicity in humans). Specific metabolic pathways are mentioned. There is a need for such an article and full submission development is supported.

Response: I am grateful for the support received for the submitted proposal and am pleased to know that it is of interest for a subsequent full submission.

In developing a full submission, there are some key suggestions:

1) Section 2.3 is superficially developed relative to other sections. The biomarker paragraphs should be far more detailed than the outline indicates, addressing biomarkers of exposure and toxicity.

Response: We added the following topics (lines 319-339):

◦ The importance of integrating exposure and effect biomarkers towards a more comprehensive understanding of how herbicides impact human health:

- The presence of herbicide metabolites in biological samples may indicate recent or cumulative herbicide exposure.[80,81]
- Herbicide adducts, formed by the covalent binding of herbicide molecules or their reactive metabolites to biological macromolecules, can serve as biomarkers of exposure. Depending on the type of biomacromolecule involved, they can indicate either recent or past exposure.[82-85]
- Herbicide exposure biomarkers can provide clues about the toxicological responses triggered by exposure, including oxidative stress,[86,87] inflammation,[88] genotoxicity,[89] and immune response disturbances[90] directly or indirectly associated with the effects of herbicides on proposed effect biomarkers.

Furthermore, in the previous sections, we included the potential toxicity mechanisms associated with the impact of herbicides on the proposed effect biomarkers, as requested by the previous reviewer, which allows for a link to the final section of the article.

2) While the focus is on shared biology, specifically shared biological responses to herbicides, a key takeaway could be expanded focus on

mechanisms that do not have shared biology. The authors should address how what is learned from their critical analysis could be used to developing herbicides that do not target human biology at sufficient magnitude to impact health.

Response: We added the following topics (lines 344-350):

- Certain herbicides specifically target plant processes, such as auxin receptors, VLCFAE enzymes, and unique biochemical pathways, which are absent in humans. However, these compounds exhibit acute toxicity in humans, and their long-term effects remain under investigation.[95]
- Understanding the nonspecific mechanisms of herbicides, including oxidative stress, inflammation, and microbiome alterations[96,97] is crucial for guiding the development of herbicides that pose less risk to human health.

Clean Manuscript File

Shared plant-human biology: herbicide effects and new biomarkers perspectives

Authors: Aline de Souza Espindola, BA, MPH, PhD, Postdoctoral[1‡], Isabella Gaichi Giannini Romaguera Undergraduate student[2†], Christine Gibson Parks BA, MSPH, PhD, Postdoctoral,[3††] Josino Costa Moreira BA, MSc, PhD[1‡‡]

[1] Occupational and Environmental Health Branch, Public Health Institute, Universidade Federal do Rio de Janeiro

[2] Biomedical Institute, Universidade Federal do Estado do Rio de Janeiro

[3] Epidemiology Branch, National Institute of Environmental Health Sciences, National Institutes of Health, Research Triangle Park, US.

‡ Associate Professor at the Universidade Federal do Rio de Janeiro

† Undergraduate student at the Universidade Federal do Estado do Rio de Janeiro

†† Research Scientist at the National Institute of Environmental Health Sciences

‡‡ Research Scientist at the Universidade Federal do Rio de Janeiro

Corresponding author:

Aline de Souza Espindola

Public Health Institute, Federal University of Rio de Janeiro

Avenida Horácio Macedo, s/n – Cidade Universitária, Rio de Janeiro – Brasil.

E-mail: esp.aline@iesc.ufrj.br

Conflict of interest disclosures

The authors declare that they have no known competing financial interests or personal relationships that could have appeared to influence the work reported in this paper.

Brief description of authorship expertise

1. Associate Professor Dr. Aline de Souza Espindola

Aline possesses skills and training in toxicology, environmental epidemiology, and laboratory sciences, with specific expertise in biomarker research. Currently, she is participating in coordinating research projects focused on the effects of pollutants on cytokines, adipokines, oxidative stress markers, autoantibodies, and the gut microbiome. As a principal investigator, her research interest is the impact of agricultural pesticide exposures on autoantibody levels, renal markers, and dysbiosis within the Kidney Health Cohort Study in Agricultural Areas in Rio de Janeiro, Brazil.

2. Research Scientist Dr. Christine Gibson Parks

Christine's background and training in biology, epidemiology, infections, and laboratory sciences, including specific expertise in immune-mediated disorders and biomarker research. Her current research also includes several studies on autoimmune diseases, inflammation, and immune dysfunction as measured by leukocyte telomere length, autoantibodies, and cytokine profiles. As a principal and co-investigator on several intramural projects, she is responsible for the design and interpretation of immune biomarker studies in the context of environmental data, including pesticide exposure and autoimmune diagnosis. She was a member of the NIEHS Inflammation Cross-Divisional Implementation Planning Committee and was the sub-committee lead for a recent webinar on novel biomarkers.

3. Research Scientist Dr. Josino Costa Moreira

Josino's background encompasses analytical chemistry, toxicology, and occupational health, with extensive cross-disciplinary research on environmental contamination and the health effects of pesticide exposures. Josino is a former senior researcher in Health and Human Ecology at the National School of Public Health at Oswaldo Cruz Foundation (ENSP/FIOCRUZ). Josino is currently a researcher in Occupational and Environmental Health at the Public Health Institute, Federal University of Rio de Janeiro, Brazil. He is

a principal and co-investigator on projects analyzing risk factors, systems, and methodologies for monitoring and evaluating health impacts and health surveillance of rural workers in the State of Rio de Janeiro.

4. Undergraduate student Ms. Isabella Gaichi Romaguera Giannini

Isabella is currently training in biomedicine and has developed research activities related to the exposure profile to pesticides in residents of rural areas.

How the seminar topic or authorship team helps diversify the environmental health sciences

Herbicide use is increasing globally, and exposure to these chemicals has been linked to poisoning and chronic diseases. Our goal is to combine knowledge from plant and human biology to identify common metabolic processes and molecular targets of herbicides that could serve as biomarkers of effect. This could be a useful tool in human biomonitoring, as well as in environmental health and occupational studies. Identifying molecular targets of herbicides in humans and discussing their underlying and broader toxicity mechanisms, which include oxidative stress, inflammation, and immunotoxicity, could also provide insight into the long-term effects of these chemicals. This interdisciplinary collaboration not only enhances the field but also encourages innovative approaches to assessing the health impacts of herbicides. The seminar topic and expertise of our authors significantly contribute to the diversification of environmental health sciences. Our team of experts is from diverse backgrounds, including toxicology, epidemiology, biology, and occupational health, enabling a multidisciplinary approach to this seminar.

Shared plant-human biology: herbicide effects and new biomarkers perspectives

Abstract

Background: Herbicide exposure has been associated with acute poisonings and chronic diseases such as autoimmune diseases, cancer, neurodegenerative disorders, and renal diseases. Humans and plants share biological processes targeted by herbicides. Investigating these shared pathways can uncover crucial insights into the underlying mechanisms of herbicides in humans and identify potential biomarkers of herbicide-induced effects.

Objectives: This seminar reviews shared biological processes between humans and plants, particularly at the biochemical level, to identify potential biomarkers of herbicide-induced effects. Additionally, it includes a discussion of the underlying mechanisms of herbicides in human disease development.

Discussion: The tubulin concentrations and key enzymes of metabolic pathways, such as hydroxyphenylpyruvate dioxygenase (HPPD), coenzyme A carboxylase (ACC), glutamine synthetase (GS), and protoporphyrinogen oxidase (PPO) are molecular targets of herbicides in humans. They seem to have relative selectivity for exposure and can be simple to collect and measure with known techniques. This seminar highlights the importance of an in-depth research effort on enzymatic pathways to understand herbicide effects on humans from the perspective of biomarkers as a tool for early detection of biological alterations before adverse events or diseases develop.

Seminar section

Shared plant-human biology: herbicide effects and new biomarkers perspectives

1. Introduction

- The first paragraph highlights the increase in herbicide use worldwide, some reasons for this increase, and the differences in herbicide consumption.[1,2]
- The second paragraph addressed the impacts of herbicides on humans.[3-10]
- The third paragraph discusses the similarities between plants and humans in metabolic processes and how this knowledge can help to identify molecular targets as potential biomarkers of the effects of herbicide exposure in humans.[12-14]

2. Discussion

2.1 Herbicide classes and their action mechanisms in plants: a brief overview

Herbicides are used to control or eliminate undesirable plants in agricultural fields and gardens and are classified into several chemical classes based on their chemical structure (Table 1). The main mechanisms of herbicides involve interferences with specific physiological or biochemical processes within plants, disrupting their growth, development, or metabolism.

Table. 1 Herbicides chemical classes and examples.

Chemical class	Examples
Phenoxyacetic acids	2,4-dichlorophenoxyacetic acid, 2,4,5-trichlorophenoxyacetic acid, mecoprop
Pyridine carboxylic acids	Picloram, clopyralid, aminopyralid
Bipyridylium compounds	Paraquat, diquat
Imidazoline	Imazapyr, imazaquin, imazamox, imazethapyr
Triazines	Atrazine, simazine, metribuzin
Glycine derivative	Glyphosate

Isoxazoles	Isoxaflutole, pyrasulfotole
Triketones	Bicyclopyrone, sulcotrione, tembotrione
Dinitroanilines	Trifluralin, pendimethalin
APPs	Diclofop-methyl, quizalofop-p-ethyl, clodinafop-propargyl
Chloroacetamide	Acetochlor, metolachlor
Diphenylethers	Aclonifen, nitrofen
Oxazolidinones	Pentoxazone
Pyrimidinediones	Benzfendizone, butafenacil, saflufenacil
N-phenylethanamides	Cinidon, flumiclorac, flumioxazin
Phosphinic acids	Glufosinate, bilanafos

Abbreviations: 4-hydroxyphenylpyruvate dioxygenase (HPPD); Aryloxy- phenoxy-propionates (APP); Protoporphyrinogen Oxidase (PPO); * Adapted by Kraehmer et al. 2014

2.1.1 Phenoxyacetic and pyridine carboxylic acids

- These herbicide classes are auxin receptor agonists that affect normal cell elongation, root development, plant growth, and tropism. They also interfere with photosynthesis and induce oxidative stress.[15-18]

2.1.2 Bipyridylium compounds

- The primary mechanisms of bipyridylium herbicides involve oxidative stress caused by excessive generation of reactive oxygen species (ROS) and interfering with photosynthesis.[19,20]

2.1.3 Imidazolinones

- These herbicides disrupt the process of photosynthesis and inhibit acetolactate synthase (ALS), a key enzyme in branched-chain amino acids biosynthesis.[21]

2.1.4 Triazines

- Triazine herbicides inhibit enzyme photosystem II (PSII) in the photosynthetic electron transport chain, disrupting ATP and NADPH synthesis.[22] They also inhibit the enzyme protoporphyrinogen oxidase (PPO), leading to protoporphyrin accumulation in the cytosol and subsequent generation of ROS.[23]

2.1.5 Glycine derivative

- Compounds derived from glycine deplete glutathione and can disrupt the antioxidant enzyme activities of glutathione.[24,25] They also inhibit 5-enolpyruvylshikimate-3-phosphate synthase (EPSPS), which is essential for the synthesis of aromatic amino acids, such as tryptophan.[26]

2.1.6 Isoxazoles and Triketones

- These herbicide classes are 4-hydroxyphenylpyruvate dioxygenase (HPPD) inhibitors that impede the conversion of 4-hydroxyphenylpyruvate, a tyrosine degradation product, to homogentisate, hindering tocopherol and plastoquinone biosynthesis.[27,28] They also interfere with carotenoid pigment synthesis, which plays a crucial role in protecting chlorophyll.[29]

2.1.7 Dinitroanilines

- Dinitroanilines interfere with the polymerization of tubulin dimers into microtubules, a protein that is essential in cell division, elongation, and overall plant growth.[30] Inhibition of microtubule formation disrupts cell division, leading to abnormalities in cell shape and overall plant growth.

2.1.8 Aryloxy- phenoxy-propionates (APP)

- APP herbicide inhibits acetyl coenzyme A carboxylase (ACC), a key enzyme in fatty acid biosynthesis. ACC catalyzes the carboxylation of acetyl-CoA to malonyl-CoA, a

crucial step in the de novo synthesis of fatty acids in plants.[31,32] Fatty acids are essential for plant growth and cell development.

2.1.9 Chloroacetamide

- These herbicides interfere with very-long-chain fatty acid elongases (VLCFAE) and geranylgeranyl pyrophosphate (GGPP) cyclization enzymes disrupting fatty acid and gibberellin production.[33,34]

2.1.10 Diphenylethers, oxazolidinones, pyrimidinediones, and N-phenylethanamides

- These herbicide classes inhibit the PPO enzyme, interfering with chlorophyll synthesis, leading to a buildup of protoporphyrinogen IX, ROS accumulation, oxidative stress, and cell death.[35-37]

2.1.11 Phosphinic acids

- These herbicides inhibit glutamine synthetase (GS), an essential enzyme that converts glutamate and ammonia to glutamine. Excessive ammonia increases ROS and decreases pigment production and photosynthesis.[38]

2.2 Shared plant-human biology: potential targets of herbicides in humans

- Plants share biological pathways with humans and can be the target of herbicides.[39,40]

2.2.1 HPPD

- HPPD is involved in the breakdown of tyrosine in humans and plants. HPPD binding sites in humans and plants are homodimers, and their structures present some variations. Herbicides seem to inhibit HPPD in mammals and humans.[41,42] The accumulation of tyrosine and its metabolites in mammals is associated with reduced enzymatic and non-enzymatic antioxidant defenses and oxidative stress, resulting in

toxicity in the eyes, liver, kidneys, and nervous system.[43,44,45,46] Commercial ELISA kits can be used to measure HPPD activity.

2.2.2 ACC

- Both human and plant ACCs catalyze the carboxylation of acetyl-CoA to produce malonyl-CoA.[47] Human ACC exists as two isoforms. ACC1 regulates fatty acid synthesis in lipogenic tissues, whereas ACC2 is responsible for fatty acid oxidation in the mitochondria of skeletal muscle, heart, and liver.[48] ACC in plants and humans are structurally similar, and herbicides appear to inhibit this enzyme in mammals.[49] Evidence for the effects of ACC inhibition by herbicides on mammals, either in vivo or in vivo, is currently lacking. However, inhibiting ACC with lipogenesis inhibitor drugs increases plasma triglyceride and very low-density lipoprotein (VLDL) levels, contributing to insulin resistance in both rodents and humans.[50,51] This inhibition can also impair T-cell expansion and alter the differentiation of regulatory T-cells, both critical for maintaining immune balance and response to infections in mammals.[52,53] ACC activity in serum and plasma humans can be measured by ELISA.

2.2.3 GS

- GS catalyzes glutamine biosynthesis from the condensation of glutamate and ammonia in humans and plants.[54] There are no major structural differences in GS between mammals and plants.[55] Glutamine is a precursor for the base of nucleic acids, neurotransmitters, and immune function regulation.[56,57,58] GS inhibition in a rare disease reduces glutamine levels and can lead to hyperammonemia,[59] impair immune cell activation, and disrupt neurotransmitter levels, contributing to neurological disorders, and immune dysregulation.[59] GS activity in serum and plasma can be measured using various techniques, including colorimetric assays and ELISA.

2.2.4 Tubulin inhibitors

- Tubulin is a protein that forms microtubules, essential cytoskeleton components. Humans express α-tubulin and β-tubulin isoforms, essential for cell division and

intracellular transport.[60] Tubulins are conserved across diverse eukaryotic lineages, and herbicides inhibit tubulin polymerization in mammals and humans.[61] Tubulin inhibitors can cause mitochondrial dysfunction, oxidative stress, genotoxicity, and cytotoxicity in mammals.[62] In vitro studies with human lymphocytes and mammal cells exposed to tubulin inhibitors have shown increased sister-chromatid exchanges, chromosomal aberrations, and micronuclei.[63,64] Tubulin concentration can be measured in serum or plasma samples by ELISA, turbidity, or fluorescence.

2.2.5 Metabolites of isoprenoid synthesis

- In humans, GGPP acts as a substrate for the post-translational modification of proteins as a precursor of vitamin K2 and ubiquinone.[65,66] There is no evidence of herbicides inhibiting GGPP pathway enzymes in humans. However, their potential toxic effects in humans include disrupting the synthesis of isoprenoids, prenylated proteins, and biomolecules involved in cell signaling, and apoptosis.[67,68] GGPP pathway enzyme deficiency in mammals has been associated with liver pathologies and severe joint inflammation in mice.[69,70] High-performance liquid chromatography and enzymatic assays are used for GGPP quantification.

2.2.6 PPO Inhibitors

- PPO is an enzyme found in humans that plays a crucial role in heme biosynthesis, an essential component of hemoglobin.[71] PPO inhibitors interfere with converting protoporphyrinogen IX to protoporphyrin IX, disrupting heme synthesis. Some studies have suggested that herbicides inhibit mammalian PPO.[72] PPO inhibition leads to the accumulation of protoporphyrinogen IX, which interferes with mitochondrial function, impairs energy production, elevates ROS levels, and leads to intermediate filament protein aggregation in mammals.[73,74] PPO activity can be measured using fluorometric assays and commercial ELISA kits.

2.3 Shared plant-human biology: herbicide effects and new biomarkers perspectives

- The first paragraph reviews biomarkers of effect definition and criteria to be ideal.[75]
- Herbicide potential targets in humans and selectivity: alterations are uncommon and can result from genetic mutations, medications, some metabolic disorders, and rare diseases.[76,77]
- Herbicide potential targets in humans: most targets of herbicides are measured by commercial kits, and some of them are used in clinical practice.[78,79]

◦ The importance of integrating exposure and effect biomarkers towards a more comprehensive understanding of how herbicides impact human health:

  - The presence of herbicide metabolites in biological samples may indicate recent or cumulative herbicide exposure.[80,81]
  - Herbicide adducts, formed by the covalent binding of herbicide molecules or their reactive metabolites to biological macromolecules, can serve as biomarkers of exposure. Depending on the type of biomacromolecule involved, they can indicate either recent or past exposure.[82-85]
  - Herbicide exposure biomarkers can provide clues about the toxicological responses triggered by exposure, including oxidative stress,[86,87] inflammation,[88] genotoxicity,[89] and immune response disturbances[90] directly or indirectly associated with the proposed effect biomarkers.

- Herbicide potential targets in humans: molecular targets of herbicides in humans are associated with immune disturbances and diseases. Discussion on the role of herbicides as environmental triggers and/or flare of diseases in predisposed individuals.[91-94]
- Certain herbicides specifically target plant processes, such as auxin receptors, VLCFAE enzymes, and unique biochemical pathways, which are absent in humans. However, these compounds exhibit acute toxicity in humans, and their long-term effects remain under investigation.[95]
- Understanding the nonspecific mechanisms of herbicides, including oxidative stress, inflammation, and microbiome alterations[96,97] is crucial for guiding the development of herbicides that pose less risk to human health.

References

1. Kniss AR. Long-term trends in the intensity and relative toxicity of herbicide use. Nat Commun. 2017;8:14865. doi: 10.1038/ncomms14865
2. Desquilbet M, Bullock DS, d'Arcangelo, FM. A discussion of the market and policy failures associated with the adoption of herbicide-tolerant crops. Int J Agr Sustain. 2019;17(5):326-337.
3. Delirrad M, Majidi M, Boushehri B. Clinical features and prognosis of paraquat poisoning: a review of 41 cases. Int J Clin Exp Med. 2015;8(5):8122-8.
4. Faria NMX, Meucci RD, Fiori NS, Carret MLV, Mello-da-Silva CA, Fassa AG. Acute Pesticide Poisoning in Tobacco Farming, According to Different Criteria. Int J Environ Res Public Health. 2023;20(4):2818. doi: 10.3390/ijerph20042818.
5. Bradberry SM, Proudfoot AT, Vale JA. Poisoning due to chlorophenoxy herbicides. Toxicol Rev. 2004;23(2):65-73. doi: 10.2165/00139709-200423020-00001.
6. Zhang L, Rana I, Shaffer RM, Taioli E, Sheppard L. Exposure to glyphosate-based herbicides and risk for non-Hodgkin lymphoma: A meta-analysis and supporting evidence. Rev Mutat Res. 2019;781:186-206. doi: 10.1016/j.mrrev.2019.02.001.
7. De Roos AJ, Fritschi L, Ward MH, Monnereau A, Hofmann J, Bernstein L, Bhatti P, et al. Herbicide use in farming and other jobs in relation to non-Hodgkin's lymphoma (NHL) risk. Occup Environ Med. 2022;79(12):795-806. doi: 10.1136/oemed-2022-108371.
8. Vaccari C, El Dib R, Gomaa H, Lopes LC, de Camargo JL. Paraquat and Parkinson's disease: a systematic review and meta-analysis of observational studies. J Toxicol Environ Health B Crit Rev. 2019;22(5-6):172-202. doi: 10.1080/10937404.2019.1659197.
9. Meyer A, Sandler DP, Beane Freeman LE, Hofmann JN, Parks CG. Pesticide Exposure and Risk of Rheumatoid Arthritis among Licensed Male Pesticide Applicators in the Agricultural Health Study. Environ Health Perspect. 2017;125(7):077010. doi: 10.1289/EHP1013.
10. Jayasumana C, Paranagama P, Agampodi S, Wijewardane C, Gunatilake S, Siribaddana S. Drinking well water and occupational exposure to Herbicides is associated with chronic kidney disease, in Padavi-Sripura, Sri Lanka. Environ Health. 2015;14:6. doi: 10.1186/1476-069X-14-6.
11. Xiaoshi L, Min L, Yang L, Zian C, Chun Z, Youwei O, Yawen Z, Yuxin L, Qi X, Chengwei Y, Jianbin L et al. An ABHD17-like hydrolase screening system to identify

de-S-acylation enzymes of protein substrates in plant cells. The Plant Cell. 2021;33,10: 3235-3249. doi:10.1093/plcell/koab199

12. Stiller, John W. Emerging genomic and proteomic evidence on relationships among the animal, plant and fungal kingdoms. Genom Proteom Bioinform. 2004;2(2):70-6. doi:10.1016/s1672-0229(04)02012-1

13. Lee, Je Min et al. Fatty Acid Desaturases, Polyunsaturated Fatty Acid Regulation, and Biotechnological Advances. Nutrients. 2016;8(1):23. doi:10.3390/nu8010023

14. Jiménez C, Cossío BR, Rivard CJ, Berl T, Capasso JM. Cell division in the unicellular microalga Dunaliella viridis depends on phosphorylation of extracellular signal-regulated kinases (ERKs). J Exp Bot. 2007;58(5):1001-11. doi: 10.1093/jxb/erl260

15. Lushchak VI, Matviishyn TM, Husak VV, Storey JM, Storey KB. Pesticide toxicity: a mechanistic approach. EXCLI J. 2018;17:1101-1136. doi: 10.17179/excli2018-1710.

16. Liu X, Qi C, Wang Z, Li Y, Wang Q, Guo M, Cao, A. Effect of picloram herbicide on physiological responses of Eupatorium adenophorum Spreng. Chil J Agric Res. 2014; 74(4);438-444.

17. Chinalia FA, Regali-Seleghin MH, Correa EM. 2, 4-D toxicity: cause, effect and control. Terrestrial and Aquatic Environmental Toxicology, 2007:1(2), 24-33.

18. Pazmiño DM, Romero-Puertas MC, Sandalio LM. Insights into the toxicity mechanism of and cell response to the herbicide 2,4-D in plants. Plant Signal Behav. 2012;7(3):425-7. doi: 10.4161/psb.19124.

19. Sukumar CA, Shanbhag V, Shastry AB. Paraquat: The Poison Potion. Indian J Crit Care Med. 2019;23(Suppl 4):S263-S266. doi: 10.5005/jp-journals-10071-23306.

20. Magalhães N, Carvalho F, Dinis-Oliveira RJ. Human and experimental toxicology of diquat poisoning: Toxicokinetics, mechanisms of toxicity, clinical features, and treatment. Hum Exp Toxicol. 2018;37(11):1131-1160. doi: 10.1177/0960327118765330.

21. Zhou Q, Liu W, Zhang Y, Liu KK. Action mechanisms of acetolactate synthase-inhibiting herbicides. Pestic Biochem Phys. 2007;89(2): 89-96.

22. Trebst A. The mode of action of triazine herbicides in plants. The Triazine Herbicides. Elsevier, 2008:101-110. doi:10.1016/B978-044451167-6.50011-8.

23. Traxler C, Gaines TA, Küpper A, Luemmen P, Dayan FE. The nexus between reactive oxygen species and the mechanism of action of herbicides. J Biol Chem. 2023;299(11):105267. doi: 10.1016/j.jbc.2023.105267.

24. Samsel A, Seneff S. Glyphosate, pathways to modern diseases III: Manganese, neurological diseases, and associated pathologies. Surg Neurol Int. 2015;6:45. doi: 10.4103/2152-7806.153876.

25. Gomes MP, Smedbol E, Chalifour A, Hénault-Ethier L, Labrecque M, Lepage L, Lucotte M, Juneau P. Alteration of plant physiology by glyphosate and its by-product aminomethylphosphonic acid: an overview. J Exp Bot. 2014;65(17):4691-703. doi: 10.1093/jxb/eru269.

26. Eceiza MV, Gil-Monreal M, Barco-Antoñanzas M, Zabalza A, Royuela M. The moderate oxidative stress induced by glyphosate is not detected in Amaranthus palmeri plants overexpressing EPSPS. J Plant Physiol. 2022;274:153720. doi: 10.1016/j.jplph.2022.153720.

27. Parthasarathy A, Cross PJ, Dobson RCJ, Adams LE, Savka MA, Hudson AO. A Three-Ring Circus: Metabolism of the Three Proteogenic Aromatic Amino Acids and Their Role in the Health of Plants and Animals. Front Mol Biosci. 2018;5:29. doi: 10.3389/fmolb.2018.00029.

28. Santucci A, Bernardini G, Braconi D, Petricci E, Manetti F. 4-Hydroxyphenylpyruvate Dioxygenase and Its Inhibition in Plants and Animals: Small Molecules as Herbicides and Agents for the Treatment of Human Inherited Diseases. J Med Chem. 2017;60(10):4101-4125. doi: 10.1021/acs.jmedchem.6b01395.

29. Jhala A, Kumar V, Yadav R, Jha P, Jugulam M, Williams M, Hausman N, Dayan F, Burto P, Dale R, Norsworthy J. 4-Hydroxyphenylpyruvate dioxygenase (HPPD)-inhibiting herbicides: Past, present, and future. Weed Technol. 2023; 37(1), 1-14. doi:10.1017/wet.2022.79

30. Chen J, Yu Q, Patterson E, Sayer C, Powles S. Dinitroaniline Herbicide Resistance and Mechanisms in Weeds. Front Plant Sci. 2021;12:634018. doi: 10.3389/fpls.2021.634018.

31. Rajak BK, Rani P, Mandal P, Chhokar RS, Singh N and Singh DV. Emerging possibilities in the advancement of herbicides to combat acetyl-CoA carboxylase inhibitor resistance. Front. Agron. 2023; 5:1218824. doi: 10.3389/fagro.2023.1218824

32. Takano HK, Ovejero RFL, Belchior GG, Maymone GPL, Dayan FE. ACCase-inhibiting herbicides: mechanism of action, resistance evolution and stewardship. Scientia Agricola. 2020; 78, e20190102.

33. Quanrud GM, Lyu Z, Balamurugan SV, Canizal C, Wu HT, Genereux JC. Cellular Exposure to Chloroacetanilide Herbicides Induces Distinct Protein Destabilization Profiles. ACS Chem Biol. 2023;18(7):1661-1676. doi: 10.1021/acschembio.3c00338.

34. Böger P, Matthes B, Schmalfuß J. Towards the primary target of chloroacetamides–new findings pave the way. Pest Management Science. 2000; 56(6):497-508.

35. Duke SO. Overview of herbicide mechanisms of action. Environ Health Perspect. 1990 Jul;87:263-71. doi: 10.1289/ehp.9087263.

36. Arnould S, Camadro JM. The domain structure of protoporphyrinogen oxidase, the molecular target of diphenyl ether-type herbicides. Proc Natl Acad Sci USA. 1998; 95(18): 10553-8. doi:10.1073/pnas.95.18.10553

37. Alves C, Costa E, Sofiatti JR, Forte CT, Winter FL, Holz CM, Kaizer RR, Galon L. Effect of herbicides in the oxidative stress in crop winter species. An Acad Bras Cienc. 2018;90(2):1533-1542. doi: 10.1590/0001-3765201820170482.

38. Vallejo B, Picazo C, Orozco H, Matallana E, Aranda A. Herbicide glufosinate inhibits yeast growth and extends longevity during wine fermentation. Sci Rep. 2017; 29;7(1):12414. doi: 10.1038/s41598-017-12794-6.

39. Martínez MA, Ares I, Rodríguez JL, Martínez M, Martínez-Larrañaga MR, Anadón A. Neurotransmitter changes in rat brain regions following glyphosate exposure. Environ Res. 2018;161:212-219. doi: 10.1016/j.envres.2017.10.051.

40. Sengupta A, Manna K, Datta S, Das U, Biswas S, Chakrabarti N, Dey S. Herbicide exposure induces apoptosis, inflammation, immune modulation and suppression of cell survival mechanism in murine model. RSC Advances. 2017;7(23): 13957-13970.

41. Antonenko A, Blagaia A, Omelchuk S, Korshun M, Vavrinevych H, Milohov D, Pelo I, Bojar I. Mechanism of action of 4-hydroxyphenylpyruvate dioxygenase inhibitor herbicide on homoterm animals and humans. J Pre Clin Clin Res. 2015;9(2):145-150. doi:10.5604/18982395.1186496.

42. Soloneski S, Larramendy ML. Herbicide sulcotrione. Herbicides, theory and applications. Eds. InTech, 2011: pp. 527–544.

43. Colemonts-Vroninks H, Neuckermans J, Marcelis L, Claes P, Branson S, Casimir G, Goyens P, Martens GA, Vanhaecke T, De Kock J. Oxidative Stress, Glutathione Metabolism, and Liver Regeneration Pathways Are Activated in Hereditary Tyrosinemia Type 1 Mice upon Short-Term Nitisinone Discontinuation. Genes (Basel). 2020;12(1):3. doi: 10.3390/genes12010003.

44. de Oliveira J, Farias HR, Streck EL. Experimental evidence of tyrosine neurotoxicity: focus on mitochondrial dysfunction. Metab Brain Dis. 2021;36(7):1673-1685. doi: 10.1007/s11011-021-00781-w.

45. Lewis RW, Botham JW. A review of the mode of toxicity and relevance to humans of the triketone herbicide 2-(4-methylsulfonyl-2-nitrobenzoyl)-1,3-cyclohexanedione. Crit Rev Toxicol. 2013;43(3):185-99. doi: 10.3109/10408444.2013.764279

46. Low K, Tasheva, M. Mesotrione. World Health Organization. 2014. Available from: https://apps.who.int/pesticide-residues-jmpr-database/Document/228

47. Brownsey RW, Zhande R, Boone AN. Isoforms of acetyl-CoA carboxylase: structures, regulatory properties and metabolic functions. Biochem Soc Trans. 1997; 25 (4): 1232–1238. doi:10.1042/bst0251232.

48. Tong L, Harwood HJ Jr. Acetyl-coenzyme A carboxylases: versatile targets for drug discovery. J Cell Biochem. 2006;99(6):1476-88. doi: 10.1002/jcb.21077.

49. Seng TW, Skillman TR, Yang N, Hammond C. Cyclohexanedione herbicides are inhibitors of rat heart acetyl-CoA carboxylase. Bioorg Med Chem Lett. 2003;13(19):3237-3242. doi:10.1016/s0960-894x(03)00664-4

50. Liu T, Gou L, Yan S, Huang T. Inhibition of acetyl-CoA carboxylase by PP-7a exerts beneficial effects on metabolic dysregulation in a mouse model of diet-induced obesity. Exp Ther Med. 2020 Jul;20(1):521-529. doi: 10.3892/etm.2020.8700.

51. Batchuluun B, Pinkosky SL, Steinberg GR. Lipogenesis inhibitors: therapeutic opportunities and challenges. Nat Rev Drug Discov. 2022 Apr;21(4):283-305. doi: 10.1038/s41573-021-00367-2.

52. Lochner M, Berod L, Sparwasser T. Fatty acid metabolism in the regulation of T cell function. Trends Immunol. 2015 Feb;36(2):81-91. doi: 10.1016/j.it.2014.12.005.

53. Byersdorfer CA. The role of Fatty Acid oxidation in the metabolic reprograming of activated t-cells. Front Immunol. 2014 Dec 18;5:641. doi: 10.3389/fimmu.2014.00641.

54. Zhang C, Yu Q, Han H, Yu C, Nyporko A, Tian X, Beckie H, Powles S. A naturally evolved mutation (Ser59Gly) in glutamine synthetase confers glufosinate resistance in plants. J Exp Bot. 2022;73(7):2251-2262. doi: 10.1093/jxb/erac008.

55. Krajewski WW, Collins R, Holmberg-Schiavone L, Jones TA, Karlberg T, Mowbray SL. Crystal structures of mammalian glutamine synthetases illustrate substrate-induced conformational changes and provide opportunities for drug and herbicide design. J Mol Biol. 2008;375(1):217-28. doi: 10.1016/j.jmb.2007.10.029.

56. Lane AN, Fan TW. Regulation of mammalian nucleotide metabolism and biosynthesis. Nucleic Acids Res. 2015;43:2466–2485. doi: 10.1093/nar/gkv047

57. Albrecht J, Sidoryk-Wegrzynowicz M, Zielinska M, Aschner M. Roles of glutamine in neurotransmission. Neuron Glia Biol. 2010;6:263–276. doi: 10.1017/S1740925X11000093.

58. Newsholme P. Why is l-glutamine metabolism important to cells of the immune system in health, postinjury, surgery or infection? J. Nutr. 2001;131:2515S–2522S. doi: 10.1093/jn/131.9.2515S.

59. Spodenkiewicz M, Diez-Fernandez C, Rüfenacht V, Gemperle-Britschgi C, Häberle J. Minireview on Glutamine Synthetase Deficiency, an Ultra-Rare Inborn Error of Amino Acid Biosynthesis. Biology (Basel). 2016 Oct 19;5(4):40. doi: 10.3390/biology5040040.

60. Moon YA, Shah NA, Mohapatra S, Warrington JA, Horton JD. Identification of a mammalian long chain fatty acyl elongase regulated by sterol regulatory element-binding proteins. J Biol Chem. 2001;276(48):45358-45366. doi:10.1074/jbc.M108413200

61. Sulimenko V, Dráberová E, Dráber P. γ-Tubulin in microtubule nucleation and beyond. Front Cell Dev Biol. 2022;10:880761. doi:10.3389/fcell.2022.880761

62. Giglio A, Vommaro ML. Dinitroaniline herbicides: a comprehensive review of toxicity and side effects on animal non-target organisms. Environ Sci Pollut Res Int. 2022 Nov;29(51):76687-76711. doi: 10.1007/s11356-022-23169-4.

63. Ribas G, Surrallés J, Carbonell E, Xamena N, Creus A, Marcos R. Genotoxic evaluation of the herbicide trifluralin on human lymphocytes exposed in vitro. Mutat Res. 1996 Nov 4;371(1-2):15-21. doi: 10.1016/s0165-1218(96)90090-7.

64. Sarıgöl Kılıç Z, Aydın S, Ündeğer Bucurgat Ü, Başaran N. In vitro genotoxicity assessment of dinitroaniline herbicides pendimethalin and trifluralin. Food Chem Toxicol. 2018 Mar;113:90-98. doi: 10.1016/j.fct.2018.01.034.

65. Muehlebach ME, Holstein SA. Geranylgeranyl diphosphate synthase: Role in human health, disease and potential therapeutic target. Clin Transl Med. 2023;13(1):e1167. doi:10.1002/ctm2.1167

66. Akula MK, Ibrahim MX, Ivarsson EG, Khan OM, Kumar IT, Erlandsson M, Karlsson C, Xu X, Brisslert M, Brakebusch C, Wang D, Bokarewa M, Sayin VI, Bergo MO. Protein prenylation restrains innate immunity by inhibiting Rac1 effector interactions. Nat Commun. 2019 Sep 4;10(1):3975. doi: 10.1038/s41467-019-11606-x.

67. Muehlebach ME, Holstein SA. Geranylgeranyl diphosphate synthase: Role in human health, disease and potential therapeutic target. Clin Transl Med. 2023 Jan;13(1):e1167. doi: 10.1002/ctm2.1167.

68. Marchwicka A, Kamińska D, Monirialamdari M, Błażewska KM, Gendaszewska-Darmach E. Protein Prenyltransferases and Their Inhibitors: Structural and Functional Characterization. International Journal of Molecular Sciences. 2022; 23(10):5424. https://doi.org/10.3390/ijms23105424

69. Yang SH, Chang SY, Tu Y, Lawson GW, Bergo MO, Fong LG, Young SG. Severe hepatocellular disease in mice lacking one or both CaaX prenyltransferases. J Lipid Res. 2012 Jan;53(1):77-86. doi: 10.1194/jlr.M021220.

70. Khan OM, Ibrahim MX, Jonsson IM, Karlsson C, Liu M, Sjogren AK, Olofsson FJ, Brisslert M, Andersson S, Ohlsson C, Hultén LM, Bokarewa M, Bergo MO. Geranylgeranyltransferase type I (GGTase-I) deficiency hyperactivates macrophages and induces erosive arthritis in mice. J Clin Invest. 2011 Feb;121(2):628-39. doi: 10.1172/JCI43758.

71. Hao GF, Zuo Y, Yang SG, Yang GF. Protoporphyrinogen oxidase inhibitor: an ideal target for herbicide discovery. Chimia (Aarau). 2011;65(12):961-969. doi:10.2533/chimia.2011.961

72. Matringe M, Camadro JM, Labbe P, Scalla, R. Protoporphyrinogen oxidase as a molecular target for diphenyl ether herbicides. Biochem. J. 260, 231–235.

73. Maitra D, Bragazzi Cunha J, Elenbaas JS, Bonkovsky HL, Shavit JA, Omary MB. Porphyrin-Induced Protein Oxidation and Aggregation as a Mechanism of Porphyria-Associated Cell Injury. Cell Mol Gastroenterol Hepatol. 2019;8(4):535-548. doi: 10.1016/j.jcmgh.2019.06.006.

74. Iwashita K, Hosokawa Y, Ihara R, Miyamoto T, Otani M, Abe J, Asano K, Mercier O, Miyata K, Barlow S. Flumioxazin, a PPO inhibitor: A weight-of-evidence consideration of its mode of action as a developmental toxicant in the rat and its relevance to humans. Toxicology. 2022 Apr 30;472:153160. doi: 10.1016/j.tox.2022.153160.

75. Byrnes SA, Weigl BH. Selecting analytical biomarkers for diagnostic applications: a first principles approach. Expert Rev Mol Diagn. 2018;18(1):19-26. doi: 10.1080/14737159.2018.1412258.

76. Jakubek M, Masařík M, Bříza T, Kaplánek R, Veselá K, Abramenko N, Martásek P. PPO-Inhibiting Herbicides and Structurally Relevant Schiff Bases: Evaluation of

Inhibitory Activities against Human Protoporphyrinogen Oxidase. Processes. 2021; 9(2):383. https://doi.org/10.3390/pr9020383
77. Tomoeda K, Awata H, Matsuura T, et al. Mutations in the 4-hydroxyphenylpyruvic acid dioxygenase gene are responsible for tyrosinemia type III and hawkinsinuria. Mol Genet Metab. 2000;71(3):506-510. doi:10.1006/mgme.2000.3085
78. Lopes-Ferreira M, Farinha LRL, Costa YSO, Pinto FJ, Disner GR, da Rosa JGDS, Lima C. Pesticide-Induced Inflammation at a Glance. Toxics. 2023;11(11):896. doi: 10.3390/toxics11110896.
79. Sidthilaw S, Sapbamrer R, Pothirat C, Wunnapuk K, Khacha-Ananda S. Effects of exposure to glyphosate on oxidative stress, inflammation, and lung function in maize farmers, Northern Thailand. BMC Public Health. 2022;22(1):1343. doi:10.1186/s12889-022-13696-7
80. Ryan PB, Burke TA, Cohen Hubal EA, Cura JJ, McKone TE. Using biomarkers to inform cumulative risk assessment. Environ Health Perspect. 2007 May;115(5):833-40. doi: 10.1289/ehp.9334.
81. Norén E, Lindh C, Rylander L, Glynn A, Axelsson J, Littorin M, Faniband M, Larsson E, Nielsen C. Concentrations and temporal trends in pesticide biomarkers in urine of Swedish adolescents, 2000-2017. J Expo Sci Environ Epidemiol. 2020 Jul;30(4):756-767. doi: 10.1038/s41370-020-0212-8. Epub 2020 Feb 24.
82. Anwar WA. Biomarkers of human exposure to pesticides. Environ Health Perspect. 1997;105 Suppl 4(Suppl 4):801-6. doi: 10.1289/ehp.97105s4801.
83. McClure GY, Freeman JP, Lay JO, Hinson JA. Haemoglobin adducts as biomarkers of exposure to the herbicides propanil and fluometuron. Biomarkers. 1996;1(2):136-40. doi: 10.3109/13547509609088681.
84. Dooley GP, Prenni JE, Prentiss PL, Cranmer BK, Andersen ME, Tessari JD. Identification of a novel hemoglobin adduct in Sprague Dawley rats exposed to atrazine. Chem Res Toxicol. 2006;19(5):692-700. doi: 10.1021/tx060023c.
85. Chu S, Letcher RJ. Bottom-up proteomics analysis for adduction of the broad-spectrum herbicide atrazine to histone. Anal Bioanal Chem. 2023;415(8):1497-1504. doi: 10.1007/s00216-023-04545-6.
86. Jabłońska-Trypuć A, Wołejko E, Wydro U, Butarewicz A. The impact of pesticides on oxidative stress level in human organism and their activity as an endocrine disruptor. J Environ Sci Health B. 2017;52(7):483-494. doi: 10.1080/03601234.2017.1303322.

87. Sule RO, Condon L, Gomes AV. A Common Feature of Pesticides: Oxidative Stress-The Role of Oxidative Stress in Pesticide-Induced Toxicity. Oxid Med Cell Longev. 2022;2022:5563759. doi: 10.1155/2022/5563759.

88. Lopes-Ferreira M, Farinha LRL, Costa YSO, Pinto FJ, Disner GR, da Rosa JGDS, Lima C. Pesticide-Induced Inflammation at a Glance. Toxics. 2023;11(11):896. doi: 10.3390/toxics11110896.

89. Bolognesi C. Genotoxicity of pesticides: a review of human biomonitoring studies. Mutat Res. 2003;543(3):251-72. doi: 10.1016/s1383-5742(03)00015-2.

90. Mokarizadeh A, Faryabi MR, Rezvanfar MA, Abdollahi M. A comprehensive review of pesticides and the immune dysregulation: mechanisms, evidence and consequences. Toxicol Mech Methods. 2015;25(4):258-78. doi: 10.3109/15376516.2015.1020182.

91. Hubler MJ, Kennedy AJ. Role of lipids in the metabolism and activation of immune cells. J Nutr Biochem. 2016;34:1-7. doi: 10.1016/j.jnutbio.2015.11.002.

Yeudall S, Upchurch CM, Seegren PV, Pavelec CM, Greulich J, Lemke MC, Harris TE,

92. Desai BN, Hoehn KL, Leitinger N. Macrophage acetyl-CoA carboxylase regulates acute inflammation through control of glucose and lipid metabolism. Sci Adv. 2022;8(47):eabq1984. doi: 10.1126/sciadv.abq1984.

93. Lee J, Walsh MC, Hoehn KL, James DE, Wherry EJ, Choi Y. Regulator of fatty acid metabolism, acetyl coenzyme a carboxylase 1, controls T cell immunity. J Immunol. 2014;192(7):3190-9. doi: 10.4049/jimmunol.1302985.

94. Kagami S, Owada T, Kanari H, Saito Y, Suto A, Ikeda K, Hirose K, Watanabe N, Iwamoto I, Nakajima H. Protein geranylgeranylation regulates the balance between Th17 cells and Foxp3+ regulatory T cells. Int Immunol. 2009;21(6):679-89. doi: 10.1093/intimm/dxp037.

95. Mohd Ghazi R, Nik Yusoff NR, Abdul Halim NS, Wahab IRA, Ab Latif N, Hasmoni SH, Ahmad Zaini MA, Zakaria ZA. Health effects of herbicides and its current removal strategies. Bioengineered. 2023;14(1):2259526. doi: 10.1080/21655979.2023.2259526.

96. Del Castilo I, Neumann AS, Lemos FS, De Bastiani MA, Oliveira FL, Zimmer ER, Rêgo AM, Hardoim CCP, Antunes LCM, Lara FA, Figueiredo CP, Clarke JR. Lifelong Exposure to a Low-Dose of the Glyphosate-Based Herbicide RoundUp® Causes Intestinal Damage, Gut Dysbiosis, and Behavioral Changes in Mice. Int J Mol Sci. 2022 May 17;23(10):5583. doi: 10.3390/ijms23105583.

97. Matsuzaki R, Gunnigle E, Geissen V, Clarke G, Nagpal J, Cryan JF. Pesticide exposure and the microbiota-gut-brain axis. ISME J. 2023 Aug;17(8):1153-1166. doi: 10.1038/s41396-023-01450-9.

Track Changes Manuscript File

Shared plant-human biology: herbicide effects and new biomarkers perspectives

Authors: Aline de Souza Espindola, BA, MPH, PhD, Postdoctoral[1‡], Isabella Gaichi Giannini Romaguera Undergraduate student[2†], Christine Gibson Parks BA, MSPH, PhD, Postdoctoral,[3††] Josino Costa Moreira BA, MSc, PhD[1‡‡]

[1] Occupational and Environmental Health Branch, Public Health Institute, Universidade Federal do Rio de Janeiro

[2] Biomedical Institute, Universidade Federal do Estado do Rio de Janeiro

[3] Epidemiology Branch, National Institute of Environmental Health Sciences, National Institutes of Health, Research Triangle Park, US.

‡ Associate Professor at the Universidade Federal do Rio de Janeiro

† Undergraduate student at the Universidade Federal do Estado do Rio de Janeiro

†† Research Scientist at the National Institute of Environmental Health Sciences

‡‡ Research Scientist at the Universidade Federal do Rio de Janeiro

Corresponding author:

Aline de Souza Espindola

Public Health Institute, Federal University of Rio de Janeiro

Avenida Horácio Macedo, s/n – Cidade Universitária, Rio de Janeiro – Brasil.

E-mail: esp.aline@iesc.ufrj.br

Formatted: Numbering: Continuous

Field Code Changed

Conflict of interest disclosures

The authors declare that they have no known competing financial interests or personal relationships that could have appeared to influence the work reported in this paper.

Brief description of authorship expertise

1. Associate Professor Dr. Aline de Souza Espindola

Aline possesses skills and training in toxicology, environmental epidemiology, and laboratory sciences, with specific expertise in biomarker research. Currently, she is participating in coordinating research projects focused on the effects of pollutants on cytokines, adipokines, oxidative stress markers, autoantibodies, and the gut microbiome. As a principal investigator, her research interest is the impact of agricultural pesticide exposures on autoantibody levels, renal markers, and dysbiosis within the Kidney Health Cohort Study in Agricultural Areas in Rio de Janeiro, Brazil.

2. Research Scientist Dr. Christine Gibson Parks

Christine's background and training in biology, epidemiology, infections, and laboratory sciences, including specific expertise in immune-mediated disorders and biomarker research. Her current research also includes several studies on autoimmune diseases, inflammation, and immune dysfunction as measured by leukocyte telomere length, autoantibodies, and cytokine profiles. As a principal and co-investigator on several intramural projects, she is responsible for the design and interpretation of immune biomarker studies in the context of environmental data, including pesticide exposure and autoimmune diagnosis. She was a member of the NIEHS Inflammation Cross-Divisional Implementation Planning Committee and was the sub-committee lead for a recent webinar on novel biomarkers.

3. Research Scientist Dr. Josino Costa Moreira

Josino's background encompasses analytical chemistry, toxicology, and occupational health, with extensive cross-disciplinary research on environmental contamination and the health effects of pesticide exposures. Josino is a former senior researcher in Health and Human Ecology at the National School of Public Health at Oswaldo Cruz Foundation (ENSP/FIOCRUZ). Josino is currently a researcher in Occupational and Environmental Health at the Public Health Institute, Federal University of Rio de Janeiro, Brazil. He is

a principal and co-investigator on projects analyzing risk factors, systems, and methodologies for monitoring and evaluating health impacts and health surveillance of rural workers in the State of Rio de Janeiro.

4. Undergraduate student Ms. Isabella Gaichi Romaguera Giannini

Isabella is currently training in biomedicine and has developed research activities related to the exposure profile to pesticides in residents of rural areas.

<u>How the seminar topic or authorship team helps diversify the environmental health sciences</u>

Herbicide use is increasing globally, and exposure to these chemicals has been linked to poisoning and chronic diseases. Our goal is to combine knowledge from plant and human biology to identify common metabolic processes and molecular targets of herbicides that could serve as biomarkers of effect. This could be a useful tool in human biomonitoring, as well as in environmental health and occupational studies. Identifying molecular targets of herbicides in humans and discussing their underlying and broader toxicity mechanisms, which include oxidative stress, inflammation, and immunotoxicity, could also provide insight into the long-term effects of these chemicals. This interdisciplinary collaboration not only enhances the field but also encourages innovative approaches to assessing the health impacts of herbicides. The seminar topic and expertise of our authors significantly contribute to the diversification of environmental health sciences. Our team of experts ~~are~~ is from diverse backgrounds, including toxicology, epidemiology, biology, and occupational health, enabling a multidisciplinary approach to this seminar.

Formatted: Font color: Red

Formatted: Font color: Red

Shared plant-human biology: herbicide effects and new biomarkers perspectives

Abstract

Background: Herbicide exposure has been associated with acute poisonings and chronic diseases such as autoimmune diseases, cancer, neurodegenerative disorders, and renal diseases. Humans and plants share biological processes targeted by herbicides. Investigating these shared pathways can uncover crucial insights into the underlying mechanisms of herbicides in humans and identify potential biomarkers of herbicide-induced effects.

Objectives: This seminar reviews shared biological processes between humans and plants, particularly at the biochemical level, to identify potential biomarkers of herbicide-induced effects. Additionally, it includes a discussion of the underlying mechanisms of herbicides in human disease development.

Discussion: The tubulin concentrations and key enzymes of metabolic pathways, such as hydroxyphenylpyruvate dioxygenase (HPPD), acetyl coenzyme A carboxylase (ACC), glutamine synthetase (GS), and protoporphyrinogen oxidase (PPO) are molecular targets of herbicides in humans. They seem to have relative selectivity for exposure and can be simple to collect and measure with known techniques. This seminar highlights the importance of ~~comprehensive~~ an in-depth research effort on enzymatic pathways~~s~~ to understand herbicide effects on humans from the perspective of biomarkers as a tool for early detection of biological alterations before adverse events or diseases develop.

Formatted: Font color: Red

Formatted: Font color: Red

Formatted: Font color: Red

Formatted: Font color: Red

Seminar section

Shared plant-human biology: herbicide effects and new biomarkers perspectives

1. Introduction

- The first paragraph highlights the increase in herbicide use worldwide, some reasons for this increase, and the differences in herbicide consumption.[1,2]
- The second paragraph addressed the impacts of herbicides on humans.[3-10]
- The third paragraph discusses the similarities between plants and humans in metabolic processes and how this knowledge can help to identify molecular targets as potential biomarkers of the effects of herbicide exposure in humans.[12-14]

2. Discussion

2.1 Herbicide classes and their action mechanisms in plants: a brief overview

Herbicides are used to control or eliminate undesirable plants in agricultural fields and gardens and are classified into several chemical classes based on their chemical structure (Table 1). The main mechanisms of herbicides involve interferences with specific physiological or biochemical processes within plants, disrupting their growth, development, or metabolism.

Table. 1 Herbicides chemical classes and examples.

**Chemical class**	**Examples**
Phenoxyacetic acids	2,4-dichlorophenoxyacetic acid, 2,4,5-trichlorophenoxyacetic acid, mecoprop
Pyridine carboxylic acids	Picloram, clopyralid, aminopyralid
Bipyridylium compounds	Paraquat, diquat
Imidazoline	Imazapyr, imazaquin, imazamox, imazethapyr
Triazines	Atrazine, simazine, metribuzin
Glycine derivative	Glyphosate
Isoxazoles	Isoxaflutole, pyrasulfotole

Triketones	Bicyclopyrone, sulcotrione, tembotrione
Dinitroanilines	Trifluralin, pendimethalin
APPs	Diclofop-methyl, quizalofop-p-ethyl, clodinafop-propargyl
Chloroacetamide	Acetochlor, metolachlor
Diphenylethers	Aclonifen, nitrofen
Oxazolidinones	Pentoxazone
Pyrimidinediones	Benzfendizone, butafenacil, saflufenacil
N-phenylethanamides	Cinidon, flumiclorac, flumioxazin
Phosphinic acids	Glufosinate, bilanafos

Abbreviations: 4-hydroxyphenylpyruvate dioxygenase (HPPD); Aryloxy- phenoxy-propionates (APP); Protoporphyrinogen Oxidase (PPO); * Adapted by Kraehmer et al. 2014

2.1.1 Phenoxyacetic and pyridine carboxylic acids

- These herbicide classes are auxin receptor agonists that affect normal cell elongation, root development, plant growth, and tropism. They also interfere with photosynthesis and induce oxidative stress.[15-18]

2.1.2 Bipyridylium compounds

- The primary mechanisms of bipyridylium herbicides involve oxidative stress caused by excessive generation of reactive oxygen species (ROS) and interfering with photosynthesis.[19,20]

2.1.3 Imidazolinones

- These herbicides disrupt the process of photosynthesis and inhibit acetolactate synthase (ALS), a key enzyme in branched-chain amino acids biosynthesis.[21]

2.1.4 Triazines

- Triazine herbicides inhibit enzyme photosystem II (PSII) in the photosynthetic electron transport chain, disrupting ATP and NADPH synthesis.[22] They also inhibit

the enzyme protoporphyrinogen oxidase (PPO), leading to protoporphyrin accumulation in the cytosol and subsequent generation of ROS.[23]

2.1.5 Glycine derivative

- Compounds derived from glycine deplete glutathione and can disrupt the antioxidant enzyme activities of glutathione.[24,25] They also inhibit 5-enolpyruvylshikimate-3-phosphate synthase (EPSPS), which is essential for the synthesis of aromatic amino acids, such as tryptophan.[26]

2.1.6 Isoxazoles and Triketones

- These herbicide classes are 4-hydroxyphenylpyruvate dioxygenase (HPPD) inhibitors that impede the conversion of 4-hydroxyphenylpyruvate, a tyrosine degradation product, to homogentisate, hindering tocopherol and plastoquinone biosynthesis.[27,28] They also interfere with carotenoid pigment synthesis, which plays a crucial role in protecting chlorophyll.[29]

2.1.7 Dinitroanilines

- Dinitroanilines interfere with the polymerization of tubulin dimers into microtubules, a protein that is essential in cell division, elongation, and overall plant growth.[30] Inhibition of microtubule formation disrupts cell division, leading to abnormalities in cell shape and overall plant growth.

2.1.8 Aryloxy- phenoxy-propionates (APP)

- APP herbicide inhibits acetyl coenzyme A carboxylase (ACC), a key enzyme in fatty acid biosynthesis. ACC catalyzes the carboxylation of acetyl-CoA to malonyl-CoA, a crucial step in the de novo synthesis of fatty acids in plants.[31,32] Fatty acids are essential for plant growth and cell development.

2.1.9 Chloroacetamide

- These herbicides interfere with very-long-chain fatty acid elongases (VLCFAE) and geranylgeranyl pyrophosphate (GGPP) cyclization enzymes disrupting fatty acid and gibberellin production.[33,34]

2.1.10 Diphenylethers, oxazolidinones, pyrimidinediones, and N-phenylethanamides

- These herbicide classes inhibit the PPO enzyme, interfering with chlorophyll synthesis, leading to a buildup of protoporphyrinogen IX, ROS accumulation, oxidative stress, and cell death.[35-37]

2.1.11 Phosphinic acids

- These herbicides inhibit glutamine synthetase (GS), an essential enzyme that converts glutamate and ammonia to glutamine. Excessive ammonia increases ROS and decreases pigment production and photosynthesis.[38]

2.2 Shared plant-human biology: potential targets of herbicides in humans

- Plants share biological pathways with humans and can be the target of herbicides.[39,40]

2.2.1 HPPD

- HPPD is involved in the breakdown of tyrosine in humans and plants. HPPD binding sites in humans and plants are homodimers, and their structures present some variations. Herbicides seem to inhibit HPPD in mammals and humans.[41,42] The accumulation of tyrosine and its metabolites in mammals is associated with reduced enzymatic and non-enzymatic antioxidant defenses and oxidative stress, resulting in toxicity in the eyes, liver, kidneys, and nervous system.[43,44,45,46] Commercial ELISA kits can be used to measure HPPD activity.

Formatted: Font color: Red

Formatted: Font color: Red, Superscript

Formatted: Font color: Red

Formatted: Font: Arial

2.2.2 ACC

- Both human and plant ACCs catalyze the carboxylation of acetyl-CoA to produce malonyl-CoA.[473] Human ACC exists as two isoforms. ACC1 regulates fatty acid

synthesis in lipogenic tissues, whereas ACC2 is responsible for fatty acid oxidation in the mitochondria of skeletal muscle, heart, and liver.[484] ACC in plants and humans are structurally similar, and herbicides appear to inhibit this enzyme in mammals.[495] Evidence for the effects of ACC inhibition by herbicides on mammals, either in vivo or in vivo, is currently lacking. However, inhibiting ACC with lipogenesis inhibitor drugs increases plasma triglyceride and very low-density lipoprotein (VLDL) levels, contributing to insulin resistance in both rodents and humans.[50,51] This inhibition can also impair T-cell expansion and alter the differentiation of regulatory T-cells, both critical for maintaining immune balance and response to infections in mammals.[52,53] ACC activity in serum and plasma humans can be measured by ELISA.

2.2.3 GS

- GS catalyzes glutamine biosynthesis from the condensation of glutamate and ammonia in humans and plants.[5446] There are no major structural differences in GS between mammals and plants.[5547] Glutamine is a precursor for the base of nucleic acids, neurotransmitters, and immune function regulation.[56,57,58] GS inhibition in a rare disease reduces glutamine levels and can lead to hyperammonemia[59], impair immune cell activation, and disrupt neurotransmitter levels, contributing to neurological disorders, and immune dysregulation.[59] GS activity in serum and plasma can be measured using various techniques, including colorimetric assays and ELISA.

2.2.4 Tubulin inhibitors

- Tubulin is a protein that forms microtubules, essential cytoskeleton components. Humans express α-tubulin and β-tubulin isoforms, essential for cell division and intracellular transport.[6048] Tubulins are conserved across diverse eukaryotic lineages, and herbicides inhibit tubulin polymerization in mammals and humans.[6149] Tubulin inhibitors can cause mitochondrial dysfunction, oxidative stress, genotoxicity, and cytotoxicity in mammals.[62] In vitro studies with human lymphocytes and mammal cells exposed to tubulin inhibitors have shown increased sister-chromatid exchanges, chromosomal aberrations, and micronuclei.[63,64] Tubulin concentration can be measured in serum or plasma samples by ELISA, turbidity, or fluorescence.

Formatted: Font color: Red
Formatted: Font color: Red, Superscript
Formatted: Font color: Red
Formatted: Font color: Red, Superscript
Formatted: Font color: Red
Formatted: Font color: Red
Formatted: Font color: Red, Superscript
Formatted: Font color: Red
Formatted: Font color: Red, Superscript
Formatted: Font color: Red
Formatted: Font: 8 pt
Formatted: Font color: Red
Formatted: Font color: Red, Superscript
Formatted: Font color: Red
Formatted: Font color: Red, Superscript
Formatted: Font color: Red

2.2.5 Metabolites of isoprenoid synthesis

- In humans, GGPP acts as a substrate for the post-translational modification of proteins as a precursor of vitamin K2 and ubiquinone.[65,6650] There is no evidence of herbicides inhibiting GGPP pathway enzymes in humans. However, their potential toxic effects in humans include disrupting the synthesis of isoprenoids, prenylated proteins, and biomolecules involved in cell signaling, and apoptosis.[67,68] GGPP pathway enzyme deficiency in mammals has been associated with liver pathologies and severe joint inflammation in mice.[69,70] High-performance liquid chromatography and enzymatic assays are used for GGPP quantification.

2.2.6 PPO Inhibitors

- PPO is an enzyme found in humans that plays a crucial role in heme biosynthesis, an essential component of hemoglobin.[7151] PPO inhibitors interfere with converting protoporphyrinogen IX to protoporphyrin IX, disrupting heme synthesis. Some studies have suggested that herbicides inhibit mammalian PPO.[752] PPO inhibition leads to the accumulation of protoporphyrinogen IX, which interferes with mitochondrial function, impairs energy production, elevates ROS levels, and leads to intermediate filament protein aggregation in mammals.[73,74] PPO activity can be measured using fluorometric assays and commercial ELISA kits.

2.3 Shared plant-human biology: herbicide effects and new biomarkers perspectives

- The first paragraph reviews biomarkers of effect definition and criteria to be ideal.[7553]
- Herbicide potential targets in humans and selectivity: alterations are uncommon and can result from genetic mutations, medications, some metabolic disorders, and rare diseases.[7654,77]
- Herbicide potential targets in humans: most targets of herbicides are measured by commercial kits, and some of them are used in clinical practice.[7856,579]
    - The importance of integrating exposure and effect biomarkers towards a more comprehensive understanding of how herbicides impact human health:

Formatted: Font color: Red
Formatted: Font color: Red, Superscript
Formatted: Font color: Red
Formatted: Font color: Red, Superscript
Formatted: Font color: Red
Formatted: Font color: Red, Superscript
Formatted: Font color: Red
Formatted: Font color: Red, Superscript
Formatted: Font color: Red
Formatted: Font color: Red
Formatted: Font color: Red, Superscript
Formatted: Font color: Red
Formatted: Font color: Red, Superscript
Formatted: Font color: Red
Formatted: Font color: Red
Formatted: Normal, Indent: Left: -0.25", Bulleted + Level: 1 + Aligned at: 0.25" + Indent at: 0.5"

- The presence of herbicide metabolites in biological samples may indicate recent or cumulative herbicide exposure.[80,81]
- Herbicide adducts, formed by the covalent binding of herbicide molecules or their reactive metabolites to biological macromolecules, can serve as biomarkers of exposure. Depending on the type of biomacromolecule involved, they can indicate either recent or past exposure.[82-85]
- Herbicide exposure biomarkers can provide clues about the toxicological responses triggered by exposure, including oxidative stress,[86,87] inflammation,[88] genotoxicity,[89] and immune response disturbances[90] directly or indirectly associated with the proposed effect biomarkers.

• Herbicide potential targets in humans: molecular targets of herbicides in humans are associated with immune disturbances and diseases. Discussion on the role of herbicides as environmental triggers and/or flare of diseases in predisposed individuals.[91-94]~~[58-61]~~

• Certain herbicides specifically target plant processes, such as auxin receptors, VLCFAE enzymes, and unique biochemical pathways, which are absent in humans. However, these compounds exhibit acute toxicity in humans, and their long-term effects remain under investigation.[95]

• Understanding the nonspecific mechanisms of herbicides, including oxidative stress, inflammation, and microbiome alterations[96,97] is crucial for guiding the development of herbicides that pose less risk to human health.

References

1. Kniss AR. Long-term trends in the intensity and relative toxicity of herbicide use. Nat Commun. 2017;8:14865. doi: 10.1038/ncomms14865

2. Desquilbet M, Bullock DS, d'Arcangelo, FM. A discussion of the market and policy failures associated with the adoption of herbicide-tolerant crops. Int J Agr Sustain. 2019;17(5):326-337.

3. Delirrad M, Majidi M, Boushehri B. Clinical features and prognosis of paraquat poisoning: a review of 41 cases. Int J Clin Exp Med. 2015;8(5):8122-8.

Formatted: Normal, No bullets or numbering
Formatted: Font color: Red, Superscript
Formatted: Font color: Red
Formatted: Font color: Red, Superscript
Formatted: Font color: Red
Formatted: Font: (Default) Times New Roman, 12 pt, Font color: Red
Formatted: Font color: Red
Formatted: Font: (Default) Times New Roman, 12 pt, Font color: Red
Formatted: Bulleted + Level: 1 + Aligned at: 0.23" + Indent at: 0.48"
Formatted: Font color: Red, Superscript
Formatted: Font: (Default) Times New Roman, 12 pt, Font color: Red
Formatted: Font color: Red, Superscript
Formatted: Font: (Default) Times New Roman, 12 pt, Font color: Red
Formatted: Font color: Red, Superscript
Formatted: Font: (Default) Times New Roman, 12 pt, Font color: Red
Formatted: Font color: Red, Superscript
Formatted: Font: (Default) Times New Roman, 12 pt, Font color: Red
Formatted: Not Superscript/ Subscript
Formatted: Font color: Red
Formatted: Indent: Left: 0", Hanging: 0.25"
Formatted: Font color: Red, Superscript
Formatted: Font color: Red

4. Faria NMX, Meucci RD, Fiori NS, Carret MLV, Mello-da-Silva CA, Fassa AG. Acute Pesticide Poisoning in Tobacco Farming, According to Different Criteria. Int J Environ Res Public Health. 2023;20(4):2818. doi: 10.3390/ijerph20042818.

5. Bradberry SM, Proudfoot AT, Vale JA. Poisoning due to chlorophenoxy herbicides. Toxicol Rev. 2004;23(2):65-73. doi: 10.2165/00139709-200423020-00001.

6. Zhang L, Rana I, Shaffer RM, Taioli E, Sheppard L. Exposure to glyphosate-based herbicides and risk for non-Hodgkin lymphoma: A meta-analysis and supporting evidence. Rev Mutat Res. 2019;781:186-206. doi: 10.1016/j.mrrev.2019.02.001.

7. De Roos AJ, Fritschi L, Ward MH, Monnereau A, Hofmann J, Bernstein L, Bhatti P, et al. Herbicide use in farming and other jobs in relation to non-Hodgkin's lymphoma (NHL) risk. Occup Environ Med. 2022;79(12):795-806. doi: 10.1136/oemed-2022-108371.

8. Vaccari C, El Dib R, Gomaa H, Lopes LC, de Camargo JL. Paraquat and Parkinson's disease: a systematic review and meta-analysis of observational studies. J Toxicol Environ Health B Crit Rev. 2019;22(5-6):172-202. doi: 10.1080/10937404.2019.1659197.

9. Meyer A, Sandler DP, Beane Freeman LE, Hofmann JN, Parks CG. Pesticide Exposure and Risk of Rheumatoid Arthritis among Licensed Male Pesticide Applicators in the Agricultural Health Study. Environ Health Perspect. 2017;125(7):077010. doi: 10.1289/EHP1013.

10. Jayasumana C, Paranagama P, Agampodi S, Wijewardane C, Gunatilake S, Siribaddana S. Drinking well water and occupational exposure to Herbicides is associated with chronic kidney disease, in Padavi-Sripura, Sri Lanka. Environ Health. 2015;14:6. doi: 10.1186/1476-069X-14-6.

11. Xiaoshi L, Min L, Yang L, Zian C, Chun Z, Youwei O, Yawen Z, Yuxin L, Qi X, Chengwei Y, Jianbin L et al. An ABHD17-like hydrolase screening system to identify de-S-acylation enzymes of protein substrates in plant cells. The Plant Cell. 2021;33,10: 3235-3249. doi:10.1093/plcell/koab199

12. Stiller, John W. Emerging genomic and proteomic evidence on relationships among the animal, plant and fungal kingdoms. Genom Proteom Bioinform. 2004;2(2):70-6. doi:10.1016/s1672-0229(04)02012-1

13. Lee, Je Min et al. Fatty Acid Desaturases, Polyunsaturated Fatty Acid Regulation, and Biotechnological Advances. Nutrients. 2016;8(1):23. doi:10.3390/nu8010023

14. Jiménez C, Cossío BR, Rivard CJ, Berl T, Capasso JM. Cell division in the unicellular microalga Dunaliella viridis depends on phosphorylation of extracellular signal-regulated kinases (ERKs). J Exp Bot. 2007;58(5):1001-11. doi: 10.1093/jxb/erl260

15. Lushchak VI, Matviishyn TM, Husak VV, Storey JM, Storey KB. Pesticide toxicity: a mechanistic approach. EXCLI J. 2018;17:1101-1136. doi: 10.17179/excli2018-1710.

16. Liu X, Qi C, Wang Z, Li Y, Wang Q, Guo M, Cao, A. Effect of picloram herbicide on physiological responses of Eupatorium adenophorum Spreng. Chil J Agric Res. 2014; 74(4);438-444.

17. Chinalia FA, Regali-Seleghin MH, Correa EM. 2, 4-D toxicity: cause, effect and control. Terrestrial and Aquatic Environmental Toxicology, 2007:1(2), 24-33.

18. Pazmiño DM, Romero-Puertas MC, Sandalio LM. Insights into the toxicity mechanism of and cell response to the herbicide 2,4-D in plants. Plant Signal Behav. 2012;7(3):425-7. doi: 10.4161/psb.19124.

19. Sukumar CA, Shanbhag V, Shastry AB. Paraquat: The Poison Potion. Indian J Crit Care Med. 2019;23(Suppl 4):S263-S266. doi: 10.5005/jp-journals-10071-23306.

20. Magalhães N, Carvalho F, Dinis-Oliveira RJ. Human and experimental toxicology of diquat poisoning: Toxicokinetics, mechanisms of toxicity, clinical features, and treatment. Hum Exp Toxicol. 2018;37(11):1131-1160. doi: 10.1177/0960327118765330.

21. Zhou Q, Liu W, Zhang Y, Liu KK. Action mechanisms of acetolactate synthase-inhibiting herbicides. Pestic Biochem Phys. 2007;89(2): 89-96.

22. Trebst A. The mode of action of triazine herbicides in plants. The Triazine Herbicides. Elsevier, 2008:101-110. doi:10.1016/B978-044451167-6.50011-8.

23. Traxler C, Gaines TA, Küpper A, Luemmen P, Dayan FE. The nexus between reactive oxygen species and the mechanism of action of herbicides. J Biol Chem. 2023;299(11):105267. doi: 10.1016/j.jbc.2023.105267.

24. Samsel A, Seneff S. Glyphosate, pathways to modern diseases III: Manganese, neurological diseases, and associated pathologies. Surg Neurol Int. 2015;6:45. doi: 10.4103/2152-7806.153876.

25. Gomes MP, Smedbol E, Chalifour A, Hénault-Ethier L, Labrecque M, Lepage L, Lucotte M, Juneau P. Alteration of plant physiology by glyphosate and its by-product aminomethylphosphonic acid: an overview. J Exp Bot. 2014;65(17):4691-703. doi: 10.1093/jxb/eru269.

26. Eceiza MV, Gil-Monreal M, Barco-Antoñanzas M, Zabalza A, Royuela M. The moderate oxidative stress induced by glyphosate is not detected in Amaranthus palmeri

plants overexpressing EPSPS. J Plant Physiol. 2022;274:153720. doi: 10.1016/j.jplph.2022.153720.
27. Parthasarathy A, Cross PJ, Dobson RCJ, Adams LE, Savka MA, Hudson AO. A Three-Ring Circus: Metabolism of the Three Proteogenic Aromatic Amino Acids and Their Role in the Health of Plants and Animals. Front Mol Biosci. 2018;5:29. doi: 10.3389/fmolb.2018.00029.
28. Santucci A, Bernardini G, Braconi D, Petricci E, Manetti F. 4-Hydroxyphenylpyruvate Dioxygenase and Its Inhibition in Plants and Animals: Small Molecules as Herbicides and Agents for the Treatment of Human Inherited Diseases. J Med Chem. 2017;60(10):4101-4125. doi: 10.1021/acs.jmedchem.6b01395.
29. Jhala A, Kumar V, Yadav R, Jha P, Jugulam M, Williams M, Hausman N, Dayan F, Burto P, Dale R, Norsworthy J. 4-Hydroxyphenylpyruvate dioxygenase (HPPD)-inhibiting herbicides: Past, present, and future. Weed Technol. 2023; 37(1), 1-14. doi:10.1017/wet.2022.79
30. Chen J, Yu Q, Patterson E, Sayer C, Powles S. Dinitroaniline Herbicide Resistance and Mechanisms in Weeds. Front Plant Sci. 2021;12:634018. doi: 10.3389/fpls.2021.634018.
31. Rajak BK, Rani P, Mandal P, Chhokar RS, Singh N and Singh DV. Emerging possibilities in the advancement of herbicides to combat acetyl-CoA carboxylase inhibitor resistance. Front. Agron. 2023; 5:1218824. doi: 10.3389/fagro.2023.1218824
32. Takano HK, Ovejero RFL, Belchior GG, Maymone GPL, Dayan FE. ACCase-inhibiting herbicides: mechanism of action, resistance evolution and stewardship. Scientia Agricola. 2020; 78, e20190102.
33. Quanrud GM, Lyu Z, Balamurugan SV, Canizal C, Wu HT, Genereux JC. Cellular Exposure to Chloroacetanilide Herbicides Induces Distinct Protein Destabilization Profiles. ACS Chem Biol. 2023;18(7):1661-1676. doi: 10.1021/acschembio.3c00338.
34. Böger P, Matthes B, Schmalfuß J. Towards the primary target of chloroacetamides–new findings pave the way. Pest Management Science. 2000; 56(6):497-508.
35. Duke SO. Overview of herbicide mechanisms of action. Environ Health Perspect. 1990 Jul;87:263-71. doi: 10.1289/ehp.9087263.
36. Arnould S, Camadro JM. The domain structure of protoporphyrinogen oxidase, the molecular target of diphenyl ether-type herbicides. Proc Natl Acad Sci USA. 1998; 95(18): 10553-8. doi:10.1073/pnas.95.18.10553

37. Alves C, Costa E, Sofiatti JR, Forte CT, Winter FL, Holz CM, Kaizer RR, Galon L. Effect of herbicides in the oxidative stress in crop winter species. An Acad Bras Cienc. 2018;90(2):1533-1542. doi: 10.1590/0001-3765201820170482.

38. Vallejo B, Picazo C, Orozco H, Matallana E, Aranda A. Herbicide glufosinate inhibits yeast growth and extends longevity during wine fermentation. Sci Rep. 2017; 29;7(1):12414. doi: 10.1038/s41598-017-12794-6.

39. Martínez MA, Ares I, Rodríguez JL, Martínez M, Martínez-Larrañaga MR, Anadón A. Neurotransmitter changes in rat brain regions following glyphosate exposure. Environ Res. 2018;161:212-219. doi: 10.1016/j.envres.2017.10.051.

40. Sengupta A, Manna K, Datta S, Das U, Biswas S, Chakrabarti N, Dey S. Herbicide exposure induces apoptosis, inflammation, immune modulation and suppression of cell survival mechanism in murine model. RSC Advances. 2017;7(23): 13957-13970.

41. Antonenko A, Blagaia A, Omelchuk S, Korshun M, Vavrinevych H, Milohov D, Pelo I, Bojar I. Mechanism of action of 4-hydroxyphenylpyruvate dioxygenase inhibitor herbicide on homoterm animals and humans. J Pre Clin Clin Res. 2015;9(2):145-150. doi:10.5604/18982395.1186496.

42. Soloneski S, Larramendy ML. Herbicide sulcotrione. Herbicides, theory and applications. Eds. InTech, 2011: pp. 527–544.

43. Colemonts-Vroninks H, Neuckermans J, Marcelis L, Claes P, Branson S, Casimir G, Goyens P, Martens GA, Vanhaecke T, De Kock J. Oxidative Stress, Glutathione Metabolism, and Liver Regeneration Pathways Are Activated in Hereditary Tyrosinemia Type 1 Mice upon Short-Term Nitisinone Discontinuation. Genes (Basel). 2020;12(1):3. doi: 10.3390/genes12010003.

44. de Oliveira J, Farias HR, Streck EL. Experimental evidence of tyrosine neurotoxicity: focus on mitochondrial dysfunction. Metab Brain Dis. 2021;36(7):1673-1685. doi: 10.1007/s11011-021-00781-w.

45. Lewis RW, Botham JW. A review of the mode of toxicity and relevance to humans of the triketone herbicide 2-(4-methylsulfonyl-2-nitrobenzoyl)-1,3-cyclohexanedione. Crit Rev Toxicol. 2013;43(3):185-99. doi: 10.3109/10408444.2013.764279

46. Low K, Tasheva, M. Mesotrione. World Health Organization. 2014. Available from: https://apps.who.int/pesticide-residues-jmpr-database/Document/228

47. Brownsey RW, Zhande R, Boone AN. Isoforms of acetyl-CoA carboxylase: structures, regulatory properties and metabolic functions. Biochem Soc Trans. 1997; 25 (4): 1232–1238. doi:10.1042/bst0251232.

Formatted: Font color: Red

48. Tong L, Harwood HJ Jr. Acetyl-coenzyme A carboxylases: versatile targets for drug discovery. J Cell Biochem. 2006;99(6):1476-88. doi: 10.1002/jcb.21077.

49. Seng TW, Skillman TR, Yang N, Hammond C. Cyclohexanedione herbicides are inhibitors of rat heart acetyl-CoA carboxylase. Bioorg Med Chem Lett. 2003;13(19):3237-3242. doi:10.1016/s0960-894x(03)00664-4

50. Liu T, Gou L, Yan S, Huang T. Inhibition of acetyl-CoA carboxylase by PP-7a exerts beneficial effects on metabolic dysregulation in a mouse model of diet-induced obesity. Exp Ther Med. 2020 Jul;20(1):521-529. doi: 10.3892/etm.2020.8700.

51. Batchuluun B, Pinkosky SL, Steinberg GR. Lipogenesis inhibitors: therapeutic opportunities and challenges. Nat Rev Drug Discov. 2022 Apr;21(4):283-305. doi: 10.1038/s41573-021-00367-2.

52. Lochner M, Berod L, Sparwasser T. Fatty acid metabolism in the regulation of T cell function. Trends Immunol. 2015 Feb;36(2):81-91. doi: 10.1016/j.it.2014.12.005.

53. Byersdorfer CA. The role of Fatty Acid oxidation in the metabolic reprograming of activated t-cells. Front Immunol. 2014 Dec 18;5:641. doi: 10.3389/fimmu.2014.00641.

54. Zhang C, Yu Q, Han H, Yu C, Nyporko A, Tian X, Beckie H, Powles S. A naturally evolved mutation (Ser59Gly) in glutamine synthetase confers glufosinate resistance in plants. J Exp Bot. 2022;73(7):2251-2262. doi: 10.1093/jxb/erac008.

55. Krajewski WW, Collins R, Holmberg-Schiavone L, Jones TA, Karlberg T, Mowbray SL. Crystal structures of mammalian glutamine synthetases illustrate substrate-induced conformational changes and provide opportunities for drug and herbicide design. J Mol Biol. 2008;375(1):217-28. doi: 10.1016/j.jmb.2007.10.029.

56. Lane AN, Fan TW. Regulation of mammalian nucleotide metabolism and biosynthesis. Nucleic Acids Res. 2015;43:2466–2485. doi: 10.1093/nar/gkv047

57. Albrecht J, Sidoryk-Wegrzynowicz M, Zielinska M, Aschner M. Roles of glutamine in neurotransmission. Neuron Glia Biol. 2010;6:263–276. doi: 10.1017/S1740925X11000093.

58. Newsholme P. Why is l-glutamine metabolism important to cells of the immune system in health, postinjury, surgery or infection? J. Nutr. 2001;131:2515S–2522S. doi: 10.1093/jn/131.9.2515S.

59. Spodenkiewicz M, Diez-Fernandez C, Rüfenacht V, Gemperle-Britschgi C, Häberle J. Minireview on Glutamine Synthetase Deficiency, an Ultra-Rare Inborn Error of Amino Acid Biosynthesis. Biology (Basel). 2016 Oct 19;5(4):40. doi: 10.3390/biology5040040.

60. Moon YA, Shah NA, Mohapatra S, Warrington JA, Horton JD. Identification of a mammalian long chain fatty acyl elongase regulated by sterol regulatory element-binding proteins. J Biol Chem. 2001;276(48):45358-45366. doi:10.1074/jbc.M108413200

61. Sulimenko V, Dráberová E, Dráber P. γ-Tubulin in microtubule nucleation and beyond. Front Cell Dev Biol. 2022;10:880761. doi:10.3389/fcell.2022.880761

62. Giglio A, Vommaro ML. Dinitroaniline herbicides: a comprehensive review of toxicity and side effects on animal non-target organisms. Environ Sci Pollut Res Int. 2022 Nov;29(51):76687-76711. doi: 10.1007/s11356-022-23169-4.

63. Ribas G, Surrallés J, Carbonell E, Xamena N, Creus A, Marcos R. Genotoxic evaluation of the herbicide trifluralin on human lymphocytes exposed in vitro. Mutat Res. 1996 Nov 4;371(1-2):15-21. doi: 10.1016/s0165-1218(96)90090-7.

64. Sarıgöl Kılıç Z, Aydın S, Ündeğer Bucurgat Ü, Başaran N. In vitro genotoxicity assessment of dinitroaniline herbicides pendimethalin and trifluralin. Food Chem Toxicol. 2018 Mar;113:90-98. doi: 10.1016/j.fct.2018.01.034.

65. Muehlebach ME, Holstein SA. Geranylgeranyl diphosphate synthase: Role in human health, disease and potential therapeutic target. Clin Transl Med. 2023;13(1):e1167. doi:10.1002/ctm2.1167

66. Akula MK, Ibrahim MX, Ivarsson EG, Khan OM, Kumar IT, Erlandsson M, Karlsson C, Xu X, Brisslert M, Brakebusch C, Wang D, Bokarewa M, Sayin VI, Bergo MO. Protein prenylation restrains innate immunity by inhibiting Rac1 effector interactions. Nat Commun. 2019 Sep 4;10(1):3975. doi: 10.1038/s41467-019-11606-x.

67. Muehlebach ME, Holstein SA. Geranylgeranyl diphosphate synthase: Role in human health, disease and potential therapeutic target. Clin Transl Med. 2023 Jan;13(1):e1167. doi: 10.1002/ctm2.1167.

68. Marchwicka A, Kamińska D, Monirialamdari M, Błażewska KM, Gendaszewska-Darmach E. Protein Prenyltransferases and Their Inhibitors: Structural and Functional Characterization. International Journal of Molecular Sciences. 2022; 23(10):5424. https://doi.org/10.3390/ijms23105424

69. Yang SH, Chang SY, Tu Y, Lawson GW, Bergo MO, Fong LG, Young SG. Severe hepatocellular disease in mice lacking one or both CaaX prenyltransferases. J Lipid Res. 2012 Jan;53(1):77-86. doi: 10.1194/jlr.M021220.

70. Khan OM, Ibrahim MX, Jonsson IM, Karlsson C, Liu M, Sjogren AK, Olofsson FJ, Brisslert M, Andersson S, Ohlsson C, Hultén LM, Bokarewa M, Bergo MO. Geranylgeranyltransferase type I (GGTase-I) deficiency hyperactivates macrophages and

Formatted: Font color: Red

Formatted: Font color: Red

induces erosive arthritis in mice. J Clin Invest. 2011 Feb;121(2):628-39. doi: 10.1172/JCI43758.

71. Hao GF, Zuo Y, Yang SG, Yang GF. Protoporphyrinogen oxidase inhibitor: an ideal target for herbicide discovery. Chimia (Aarau). 2011;65(12):961-969. doi:10.2533/chimia.2011.961

72. Matringe M, Camadro JM, Labbe P, Scalla, R. Protoporphyrinogen oxidase as a molecular target for diphenyl ether herbicides. Biochem. J. 260, 231–235.

73. Maitra D, Bragazzi Cunha J, Elenbaas JS, Bonkovsky HL, Shavit JA, Omary MB. Porphyrin-Induced Protein Oxidation and Aggregation as a Mechanism of Porphyria-Associated Cell Injury. Cell Mol Gastroenterol Hepatol. 2019;8(4):535-548. doi: 10.1016/j.jcmgh.2019.06.006.

74. Iwashita K, Hosokawa Y, Ihara R, Miyamoto T, Otani M, Abe J, Asano K, Mercier O, Miyata K, Barlow S. Flumioxazin, a PPO inhibitor: A weight-of-evidence consideration of its mode of action as a developmental toxicant in the rat and its relevance to humans. Toxicology. 2022 Apr 30;472:153160. doi: 10.1016/j.tox.2022.153160.

75. Byrnes SA, Weigl BH. Selecting analytical biomarkers for diagnostic applications: a first principles approach. Expert Rev Mol Diagn. 2018;18(1):19-26. doi: 10.1080/14737159.2018.1412258.

76. Jakubek M, Masařík M, Bříza T, Kaplánek R, Veselá K, Abramenko N, Martásek P. PPO-Inhibiting Herbicides and Structurally Relevant Schiff Bases: Evaluation of Inhibitory Activities against Human Protoporphyrinogen Oxidase. Processes. 2021; 9(2):383. https://doi.org/10.3390/pr9020383

77. Tomoeda K, Awata H, Matsuura T, et al. Mutations in the 4-hydroxyphenylpyruvic acid dioxygenase gene are responsible for tyrosinemia type III and hawkinsinuria. Mol Genet Metab. 2000;71(3):506-510. doi:10.1006/mgme.2000.3085

78. Lopes-Ferreira M, Farinha LRL, Costa YSO, Pinto FJ, Disner GR, da Rosa JGDS, Lima C. Pesticide-Induced Inflammation at a Glance. Toxics. 2023;11(11):896. doi: 10.3390/toxics11110896.

79. Sidthilaw S, Sapbamrer R, Pothirat C, Wunnapuk K, Khacha-Ananda S. Effects of exposure to glyphosate on oxidative stress, inflammation, and lung function in maize farmers, Northern Thailand. BMC Public Health. 2022;22(1):1343. doi:10.1186/s12889-022-13696-7

Formatted: English (United States)

Formatted: Font color: Red

Formatted: Portuguese (Brazil)

80. Ryan PB, Burke TA, Cohen Hubal EA, Cura JJ, McKone TE. Using biomarkers to inform cumulative risk assessment. Environ Health Perspect. 2007 May;115(5):833-40. doi: 10.1289/ehp.9334.

81. Norén E, Lindh C, Rylander L, Glynn A, Axelsson J, Littorin M, Faniband M, Larsson E, Nielsen C. Concentrations and temporal trends in pesticide biomarkers in urine of Swedish adolescents, 2000-2017. J Expo Sci Environ Epidemiol. 2020 Jul;30(4):756-767. doi: 10.1038/s41370-020-0212-8. Epub 2020 Feb 24.

82. Anwar WA. Biomarkers of human exposure to pesticides. Environ Health Perspect. 1997;105 Suppl 4(Suppl 4):801-6. doi: 10.1289/ehp.97105s4801.

83. McClure GY, Freeman JP, Lay JO, Hinson JA. Haemoglobin adducts as biomarkers of exposure to the herbicides propanil and fluometuron. Biomarkers. 1996;1(2):136-40. doi: 10.3109/13547509609088681.

84. Dooley GP, Prenni JE, Prentiss PL, Cranmer BK, Andersen ME, Tessari JD. Identification of a novel hemoglobin adduct in Sprague Dawley rats exposed to atrazine. Chem Res Toxicol. 2006;19(5):692-700. doi: 10.1021/tx060023c.

85. Chu S, Letcher RJ. Bottom-up proteomics analysis for adduction of the broad-spectrum herbicide atrazine to histone. Anal Bioanal Chem. 2023;415(8):1497-1504. doi: 10.1007/s00216-023-04545-6.

86. Jabłońska-Trypuć A, Wołejko E, Wydro U, Butarewicz A. The impact of pesticides on oxidative stress level in human organism and their activity as an endocrine disruptor. J Environ Sci Health B. 2017;52(7):483-494. doi: 10.1080/03601234.2017.1303322.

87. Sule RO, Condon L, Gomes AV. A Common Feature of Pesticides: Oxidative Stress-The Role of Oxidative Stress in Pesticide-Induced Toxicity. Oxid Med Cell Longev. 2022;2022:5563759. doi: 10.1155/2022/5563759.

88. Lopes-Ferreira M, Farinha LRL, Costa YSO, Pinto FJ, Disner GR, da Rosa JGDS, Lima C. Pesticide-Induced Inflammation at a Glance. Toxics. 2023;11(11):896. doi: 10.3390/toxics11110896.

89. Bolognesi C. Genotoxicity of pesticides: a review of human biomonitoring studies. Mutat Res. 2003;543(3):251-72. doi: 10.1016/s1383-5742(03)00015-2.

90. Mokarizadeh A, Faryabi MR, Rezvanfar MA, Abdollahi M. A comprehensive review of pesticides and the immune dysregulation: mechanisms, evidence and consequences. Toxicol Mech Methods. 2015;25(4):258-78. doi: 10.3109/15376516.2015.1020182.

91. Hubler MJ, Kennedy AJ. Role of lipids in the metabolism and activation of immune cells. J Nutr Biochem. 2016;34:1-7. doi: 10.1016/j.jnutbio.2015.11.002.

Formatted: Font color: Red

Formatted: Font color: Red, Portuguese (Brazil)

Formatted: Font color: Red

Formatted: Font color: Red, English (United States)

Formatted: Font color: Red

Yeudall S, Upchurch CM, Seegren PV, Pavelec CM, Greulich J, Lemke MC, Harris TE,

92. Desai BN, Hoehn KL, Leitinger N. Macrophage acetyl-CoA carboxylase regulates acute inflammation through control of glucose and lipid metabolism. Sci Adv. 2022;8(47):eabq1984. doi: 10.1126/sciadv.abq1984.

93. Lee J, Walsh MC, Hoehn KL, James DE, Wherry EJ, Choi Y. Regulator of fatty acid metabolism, acetyl coenzyme a carboxylase 1, controls T cell immunity. J Immunol. 2014;192(7):3190-9. doi: 10.4049/jimmunol.1302985.

94. Kagami S, Owada T, Kanari H, Saito Y, Suto A, Ikeda K, Hirose K, Watanabe N, Iwamoto I, Nakajima H. Protein geranylgeranylation regulates the balance between Th17 cells and Foxp3+ regulatory T cells. Int Immunol. 2009;21(6):679-89. doi: 10.1093/intimm/dxp037.

95. Mohd Ghazi R, Nik Yusoff NR, Abdul Halim NS, Wahab IRA, Ab Latif N, Hasmoni SH, Ahmad Zaini MA, Zakaria ZA. Health effects of herbicides and its current removal strategies. Bioengineered. 2023;14(1):2259526. doi: 10.1080/21655979.2023.2259526.

96. Del Castilo I, Neumann AS, Lemos FS, De Bastiani MA, Oliveira FL, Zimmer ER, Rêgo AM, Hardoim CCP, Antunes LCM, Lara FA, Figueiredo CP, Clarke JR. Lifelong Exposure to a Low-Dose of the Glyphosate-Based Herbicide RoundUp® Causes Intestinal Damage, Gut Dysbiosis, and Behavioral Changes in Mice. Int J Mol Sci. 2022 May 17;23(10):5583. doi: 10.3390/ijms23105583.

97. Matsuzaki R, Gunnigle E, Geissen V, Clarke G, Nagpal J, Cryan JF. Pesticide exposure and the microbiota-gut-brain axis. ISME J. 2023 Aug;17(8):1153-1166. doi: 10.1038/s41396-023-01450-9.

Formatted: Font color: Red

Formatted: Font color: Red, English (United States)

Formatted: Font color: Red

Formatted: Font color: Red, Portuguese (Brazil)

Formatted: Font color: Red
