# Supplementary material for: Shared Plant-human Biology: Herbicide Effects and New Biomarkers Perspectives
Source: Curr Environ Health Rep. 2026 Apr 2;13(1):16. doi: 10.1007/s40572-026-00523-z (PMC13046696; doi:10.1007/s40572-026-00523-z)
Supplement: Supplementary file 3 — Supplementary Material 3 (DOCX 41.9 KB) [file 40572_2026_523_MOESM3_ESM.docx]

**Shared plant-human biology: herbicide effects and new biomarkers perspectives**

**Supplemental Material**

**Acetyl Coa Carboxilase - ACC (*Arabidopsis thaliana* and *Homo sapiens)***

**Entire carboxyltransferase domain of ACC1 from *Arabidopsis thaliana* and *Homo sapiens***

***UNIPROT***

1. Human (Homo sapiens): ACACA_HUMAN (Q13085)

- <https://www.uniprot.org/uniprotkb/Q13085/entry>
- Region: 1576–2234 (Carboxyltransferase)

**Sequence:**PYVTKDLLQSKRFQAQSLGTTYIYDIPEMFRQSLIKLWESMSTQAFLPSPPLPSDMLTYTELVLDDQGQLVHMNRLPGGNEIGMVAWKMTFKSPEYPEGRDIIVIGNDITYRIGSFGPQEDLLFLRASELARAEGIPRIYVSANSGARIGLAEEIRHMFHVAWVDPEDPYKGYRYLYLTPQDYKRVSALNSVHCEHVEDEGESRYKITDIIGKEEGIGPENLRGSGMIAGESSLAYNEIITISLVTCRAIGIGAYLVRLGQRTIQVENSHLILTGAGALNKVLGREVYTSNNQLGGIQIMHNNGVTHCTVCDDFEGVFTVLHWLSYMPKSVHSSVPLLNSKDPIDRIIEFVPTKTPYDPRWMLAGRPHPTQKGQWLSGFFDYGSFSEIMQPWAQTVVVGRARLGGIPVGVVAVETRTVELSIPADPANLDSEAKIIQQAGQVWFPDSAFKTYQAIKDFNREGLPLMVFANWRGFSGGMKDMYDQVLKFGAYIVDGLRECCQPVLVYIPPQAELRGGSWVVIDSSINPRHMEMYADRESRGSVLEPEGTVEIKFRRKDLVKTMRRVDPVYIHLAERLGTPELSTAERKELENKLKEREEFLIPIYHQVAVQFADLHDTPGRMQEKGVISDILDWKTSRTFFYWRLRRLLLEDLVKKKIHN

2. Plant (Arabidopsis thaliana): ACC1_ARATH (Q38970)

- <https://www.uniprot.org/uniprotkb/Q38970/entry>
- Region: 1492–2150 (Carboxyltransferase)

**Sequence:**QYKPLGYLDRQRLAARRSNTTYCYDFPLAFGTALELLWASQHPGVKKPYKDTLINVKELVFSKPEGSSGTSLDLVERPPGLNDFGMVAWCLDMSTPEFPMGRKLLVIANDVTFKAGSFGPREDAFFLAVTELACAKKLPLIYLAANSGARLGVAEEVKACFKVGWSDEISPENGFQYIYLSPEDHERIGSSVIAHEVKLSSGETRWVIDTIVGKEDGIGVENLTGSGAIAGAYSKAYNETFTLTFVSGRTVGIGAYLARLGMRCIQRLDQPIILTGFSTLNKLLGREVYSSHMQLGGPKIMGTNGVVHLTVSDDLEGVSAILNWLSYIPAYVGGPLPVLAPLDPPERIVEYVPENSCDPRAAIAGVKDNTGKWLGGIFDKNSFIETLEGWARTVVTGRAKLGGIPVGVVAVETQTVMQIIPADPGQLDSHERVVPQAGQVWFPDSAAKTAQALMDFNREELPLFILANWRGFSGGQRDLFEGILQAGSTIVENLRTYRQPVFVYIPMMGELRGGAWVVVDSQINSDYVEMYADETARGNVLEPEGTIEIKFRTKELLECMGRLDQKLISLKAKLQDAKQSEAYANIELLQQQIKAREKQLLPVYIQIATKFAELHDTSMRMAAKGVIKSVVEWSGSRSFFYKKLNRRIAESSLVKNVRE

***EMBL-EBI***

- <https://www.ebi.ac.uk/jdispatcher/psa/emboss_needle>

**Results for Job ID:** emboss_needle-I20251214-200953-0355-93124045-p1m

########################################

# Program: needle

# Rundate: Sun 14 Dec 2025 20:09:57

# Commandline: needle

# -auto

# -stdout

# -asequence emboss_needle-I20251214-200953-0355-93124045-p1m.asequence

# -bsequence emboss_needle-I20251214-200953-0355-93124045-p1m.bsequence

# -datafile EBLOSUM62

# -gapopen 10.0

# -gapextend 0.5

# -endopen 10.0

# -endextend 0.5

# -aformat3 pair

# -sprotein1

# -sprotein2

# Align_format: pair

# Report_file: stdout

########################################

#=======================================

#

# Aligned_sequences: 2

# 1: EMBOSS_001

# 2: EMBOSS_001

# Matrix: EBLOSUM62

# Gap_penalty: 10.0

# Extend_penalty: 0.5

#

# Length: 667

# Identity: 332/667 (49.8%)

# Similarity: 442/667 (66.3%)

# Gaps: 16/667 ( 2.4%)

# Score: 1635.0

#

#

#=======================================

EMBOSS_001 1 PYVTKDLLQSKRFQAQSLGTTYIYDIPEMFRQSLIKLWESMSTQAFLPSP 50

.|.....|..:|..|:...|||.||.|..|..:|..||.|..... ..

EMBOSS_001 1 QYKPLGYLDRQRLAARRSNTTYCYDFPLAFGTALELLWASQHPGV---KK 47

EMBOSS_001 51 PLPSDMLTYTELVLD-DQG----QLVHMNRLPGGNEIGMVAWKMTFKSPE 95

|....::...|||.. .:| .|..:.|.||.|:.|||||.:...:||

EMBOSS_001 48 PYKDTLINVKELVFSKPEGSSGTSLDLVERPPGLNDFGMVAWCLDMSTPE 97

EMBOSS_001 96 YPEGRDIIVIGNDITYRIGSFGPQEDLLFLRASELARAEGIPRIYVSANS 145

:|.||.::||.||:|::.|||||:||..||..:|||.|:.:|.||::|||

EMBOSS_001 98 FPMGRKLLVIANDVTFKAGSFGPREDAFFLAVTELACAKKLPLIYLAANS 147

EMBOSS_001 146 GARIGLAEEIRHMFHVAWVDPEDPYKGYRYLYLTPQDYKRVSALNSVHCE 195

|||:|:|||::..|.|.|.|...|..|::|:||:|:|::|:. :||...

EMBOSS_001 148 GARLGVAEEVKACFKVGWSDEISPENGFQYIYLSPEDHERIG--SSVIAH 195

EMBOSS_001 196 HVE-DEGESRYKITDIIGKEEGIGPENLRGSGMIAGESSLAYNEIITISL 244

.|: ..||:|:.|..|:|||:|||.|||.|||.|||..|.||||..|::.

EMBOSS_001 196 EVKLSSGETRWVIDTIVGKEDGIGVENLTGSGAIAGAYSKAYNETFTLTF 245

EMBOSS_001 245 VTCRAIGIGAYLVRLGQRTIQVENSHLILTGAGALNKVLGREVYTSNNQL 294

|:.|.:||||||.|||.|.||..:..:||||...|||:||||||:|:.||

EMBOSS_001 246 VSGRTVGIGAYLARLGMRCIQRLDQPIILTGFSTLNKLLGREVYSSHMQL 295

EMBOSS_001 295 GGIQIMHNNGVTHCTVCDDFEGVFTVLHWLSYMPKSVHSSVPLLNSKDPI 344

||.:||..|||.|.||.||.|||..:|:||||:|..|...:|:|...||.

EMBOSS_001 296 GGPKIMGTNGVVHLTVSDDLEGVSAILNWLSYIPAYVGGPLPVLAPLDPP 345

EMBOSS_001 345 DRIIEFVPTKTPYDPRWMLAGRPHPTQKGQWLSGFFDYGSFSEIMQPWAQ 394

:||:|:|| :...|||..:||....| |:||.|.||..||.|.::.||:

EMBOSS_001 346 ERIVEYVP-ENSCDPRAAIAGVKDNT--GKWLGGIFDKNSFIETLEGWAR 392

EMBOSS_001 395 TVVVGRARLGGIPVGVVAVETRTVELSIPADPANLDSEAKIIQQAGQVWF 444

|||.|||:|||||||||||||:||...|||||..|||..:::.|||||||

EMBOSS_001 393 TVVTGRAKLGGIPVGVVAVETQTVMQIIPADPGQLDSHERVVPQAGQVWF 442

EMBOSS_001 445 PDSAFKTYQAIKDFNREGLPLMVFANWRGFSGGMKDMYDQVLKFGAYIVD 494

||||.||.||:.|||||.|||.:.|||||||||.:|:::.:|:.|:.||:

EMBOSS_001 443 PDSAAKTAQALMDFNREELPLFILANWRGFSGGQRDLFEGILQAGSTIVE 492

EMBOSS_001 495 GLRECCQPVLVYIPPQAELRGGSWVVIDSSINPRHMEMYADRESRGSVLE 544

.||...|||.||||...|||||:|||:||.||..::|||||..:||:|||

EMBOSS_001 493 NLRTYRQPVFVYIPMMGELRGGAWVVVDSQINSDYVEMYADETARGNVLE 542

EMBOSS_001 545 PEGTVEIKFRRKDLVKTMRRVDPVYIHLAERLGTPELST--AERKELENK 592

||||:|||||.|:|::.|.|:|...|.|..:|...:.|. |..:.|:.:

EMBOSS_001 543 PEGTIEIKFRTKELLECMGRLDQKLISLKAKLQDAKQSEAYANIELLQQQ 592

EMBOSS_001 593 LKEREEFLIPIYHQVAVQFADLHDTPGRMQEKGVISDILDWKTSRTFFYW 642

:|.||:.|:|:|.|:|.:||:||||..||..||||..:::|..||:|||.

EMBOSS_001 593 IKAREKQLLPVYIQIATKFAELHDTSMRMAAKGVIKSVVEWSGSRSFFYK 642

EMBOSS_001 643 RLRRLLLEDLVKKKIHN 659

:|.|.:.|..:.|.:..

EMBOSS_001 643 KLNRRIAESSLVKNVRE 659

#---------------------------------------

#---------------------------------------

**N-terminal subdomain of the CoA carboxyltransferase of the ACC from Arabidopsis thaliana and Homo sapiens**

***UNIPROT***

1. Human (Homo sapiens): ACACA_HUMAN (Q13085)

- <https://www.uniprot.org/uniprotkb/Q13085/entry>
- Region: 1576-1914 (CoA carboxyltransferase N-terminal)

**Sequence:**PYVTKDLLQSKRFQAQSLGTTYIYDIPEMFRQSLIKLWESMSTQAFLPSPPLPSDMLTYTELVLDDQGQLVHMNRLPGGNEIGMVAWKMTFKSPEYPEGRDIIVIGNDITYRIGSFGPQEDLLFLRASELARAEGIPRIYVSANSGARIGLAEEIRHMFHVAWVDPEDPYKGYRYLYLTPQDYKRVSALNSVHCEHVEDEGESRYKITDIIGKEEGIGPENLRGSGMIAGESSLAYNEIITISLVTCRAIGIGAYLVRLGQRTIQVENSHLILTGAGALNKVLGREVYTSNNQLGGIQIMHNNGVTHCTVCDDFEGVFTVLHWLSYMPKSVHSSVPLLN

2. Plant (Arabidopsis thaliana): Q38970 · ACC1_ARATH

- <https://www.uniprot.org/uniprotkb/Q38970/entry>
- Region: 1492-1831 (CoA carboxyltransferase N-terminal)

**Sequence:**QYKPLGYLDRQRLAARRSNTTYCYDFPLAFGTALELLWASQHPGVKKPYKDTLINVKELVFSKPEGSSGTSLDLVERPPGLNDFGMVAWCLDMSTPEFPMGRKLLVIANDVTFKAGSFGPREDAFFLAVTELACAKKLPLIYLAANSGARLGVAEEVKACFKVGWSDEISPENGFQYIYLSPEDHERIGSSVIAHEVKLSSGETRWVIDTIVGKEDGIGVENLTGSGAIAGAYSKAYNETFTLTFVSGRTVGIGAYLARLGMRCIQRLDQPIILTGFSTLNKLLGREVYSSHMQLGGPKIMGTNGVVHLTVSDDLEGVSAILNWLSYIPAYVGGPLPVLA

***EMBL-EBI***

<https://www.ebi.ac.uk/jdispatcher/psa/emboss_needle>

**Results for Job ID:** emboss_needle-I20251214-203124-0428-45520497-p1m

CoA carboxyltransferase N-terminal

########################################

# Program: needle

# Rundate: Sun 14 Dec 2025 20:31:28

# Commandline: needle

# -auto

# -stdout

# -asequence emboss_needle-I20251214-203124-0428-45520497-p1m.asequence

# -bsequence emboss_needle-I20251214-203124-0428-45520497-p1m.bsequence

# -datafile EBLOSUM62

# -gapopen 10.0

# -gapextend 0.5

# -endopen 10.0

# -endextend 0.5

# -aformat3 pair

# -sprotein1

# -sprotein2

# Align_format: pair

# Report_file: stdout

########################################

#=======================================

#

# Aligned_sequences: 2

# 1: EMBOSS_001

# 2: EMBOSS_001

# Matrix: EBLOSUM62

# Gap_penalty: 10.0

# Extend_penalty: 0.5

#

# Length: 345

# Identity: 161/345 (46.7%)

# Similarity: 216/345 (62.6%)

# Gaps: 11/345 ( 3.2%)

# Score: 767.0

#

#

#=======================================

EMBOSS_001 1 PYVTKDLLQSKRFQAQSLGTTYIYDIPEMFRQSLIKLWESMSTQAFLPSP 50

.|.....|..:|..|:...|||.||.|..|..:|..||.|..... ..

EMBOSS_001 1 QYKPLGYLDRQRLAARRSNTTYCYDFPLAFGTALELLWASQHPGV---KK 47

EMBOSS_001 51 PLPSDMLTYTELVLD-DQG----QLVHMNRLPGGNEIGMVAWKMTFKSPE 95

|....::...|||.. .:| .|..:.|.||.|:.|||||.:...:||

EMBOSS_001 48 PYKDTLINVKELVFSKPEGSSGTSLDLVERPPGLNDFGMVAWCLDMSTPE 97

EMBOSS_001 96 YPEGRDIIVIGNDITYRIGSFGPQEDLLFLRASELARAEGIPRIYVSANS 145

:|.||.::||.||:|::.|||||:||..||..:|||.|:.:|.||::|||

EMBOSS_001 98 FPMGRKLLVIANDVTFKAGSFGPREDAFFLAVTELACAKKLPLIYLAANS 147

EMBOSS_001 146 GARIGLAEEIRHMFHVAWVDPEDPYKGYRYLYLTPQDYKRVSALNSVHCE 195

|||:|:|||::..|.|.|.|...|..|::|:||:|:|::|:. :||...

EMBOSS_001 148 GARLGVAEEVKACFKVGWSDEISPENGFQYIYLSPEDHERIG--SSVIAH 195

EMBOSS_001 196 HVE-DEGESRYKITDIIGKEEGIGPENLRGSGMIAGESSLAYNEIITISL 244

.|: ..||:|:.|..|:|||:|||.|||.|||.|||..|.||||..|::.

EMBOSS_001 196 EVKLSSGETRWVIDTIVGKEDGIGVENLTGSGAIAGAYSKAYNETFTLTF 245

EMBOSS_001 245 VTCRAIGIGAYLVRLGQRTIQVENSHLILTGAGALNKVLGREVYTSNNQL 294

|:.|.:||||||.|||.|.||..:..:||||...|||:||||||:|:.||

EMBOSS_001 246 VSGRTVGIGAYLARLGMRCIQRLDQPIILTGFSTLNKLLGREVYSSHMQL 295

EMBOSS_001 295 GGIQIMHNNGVTHCTVCDDFEGVFTVLHWLSYMPKSVHSSVPLLN 339

||.:||..|||.|.||.||.|||..:|:||||:|..|...:|:|.

EMBOSS_001 296 GGPKIMGTNGVVHLTVSDDLEGVSAILNWLSYIPAYVGGPLPVLA 340

#---------------------------------------

#---------------------------------------

**C-terminal subdomain of the CoA carboxyltransferase of ACC from Arabidopsis thaliana and Homo sapiens**

***UNIPROT***

1. Human (Homo sapiens): ACACA_HUMAN (Q13085)

- <https://www.uniprot.org/uniprotkb/Q13085/entry>
- Region: 1918-2234 (CoA carboxyltransferase C-terminal)

**Sequence:**PIDRIIEFVPTKTPYDPRWMLAGRPHPTQKGQWLSGFFDYGSFSEIMQPWAQTVVVGRARLGGIPVGVVAVETRTVELSIPADPANLDSEAKIIQQAGQVWFPDSAFKTYQAIKDFNREGLPLMVFANWRGFSGGMKDMYDQVLKFGAYIVDGLRECCQPVLVYIPPQAELRGGSWVVIDSSINPRHMEMYADRESRGSVLEPEGTVEIKFRRKDLVKTMRRVDPVYIHLAERLGTPELSTAERKELENKLKEREEFLIPIYHQVAVQFADLHDTPGRMQEKGVISDILDWKTSRTFFYWRLRRLLLEDLVKKKIHN

2. Plant (Arabidopsis thaliana): Q38970 · ACC1_ARATH

- <https://www.uniprot.org/uniprotkb/Q38970/entry>
- Region: 1835-2150 (CoA carboxyltransferase C-terminal)

**Sequence:**PPERIVEYVPENSCDPRAAIAGVKDNTGKWLGGIFDKNSFIETLEGWARTVVTGRAKLGGIPVGVVAVETQTVMQIIPADPGQLDSHERVVPQAGQVWFPDSAAKTAQALMDFNREELPLFILANWRGFSGGQRDLFEGILQAGSTIVENLRTYRQPVFVYIPMMGELRGGAWVVVDSQINSDYVEMYADETARGNVLEPEGTIEIKFRTKELLECMGRLDQKLISLKAKLQDAKQSEAYANIELLQQQIKAREKQLLPVYIQIATKFAELHDTSMRMAAKGVIKSVVEWSGSRSFFYKKLNRRIAESSLVKNVRE

***EMBL-EBI***

**Results for Job ID:** emboss_needle-I20251214-203939-0861-32894654-p1m

CoA carboxyltransferase C-terminal

########################################

# Program: needle

# Rundate: Sun 14 Dec 2025 20:39:51

# Commandline: needle

# -auto

# -stdout

# -asequence emboss_needle-I20251214-203939-0861-32894654-p1m.asequence

# -bsequence emboss_needle-I20251214-203939-0861-32894654-p1m.bsequence

# -datafile EBLOSUM62

# -gapopen 10.0

# -gapextend 0.5

# -endopen 10.0

# -endextend 0.5

# -aformat3 pair

# -sprotein1

# -sprotein2

# Align_format: pair

# Report_file: stdout

########################################

#=======================================

#

# Aligned_sequences: 2

# 1: EMBOSS_001

# 2: EMBOSS_001

# Matrix: EBLOSUM62

# Gap_penalty: 10.0

# Extend_penalty: 0.5

#

# Length: 319

# Identity: 170/319 (53.3%)

# Similarity: 225/319 (70.5%)

# Gaps: 5/319 ( 1.6%)

# Score: 865.0

#

#

#=======================================

EMBOSS_001 1 PIDRIIEFVPTKTPYDPRWMLAGRPHPTQKGQWLSGFFDYGSFSEIMQPW 50

|.:||:|:|| :...|||..:||....| |:||.|.||..||.|.::.|

EMBOSS_001 1 PPERIVEYVP-ENSCDPRAAIAGVKDNT--GKWLGGIFDKNSFIETLEGW 47

EMBOSS_001 51 AQTVVVGRARLGGIPVGVVAVETRTVELSIPADPANLDSEAKIIQQAGQV 100

|:|||.|||:|||||||||||||:||...|||||..|||..:::.|||||

EMBOSS_001 48 ARTVVTGRAKLGGIPVGVVAVETQTVMQIIPADPGQLDSHERVVPQAGQV 97

EMBOSS_001 101 WFPDSAFKTYQAIKDFNREGLPLMVFANWRGFSGGMKDMYDQVLKFGAYI 150

||||||.||.||:.|||||.|||.:.|||||||||.:|:::.:|:.|:.|

EMBOSS_001 98 WFPDSAAKTAQALMDFNREELPLFILANWRGFSGGQRDLFEGILQAGSTI 147

EMBOSS_001 151 VDGLRECCQPVLVYIPPQAELRGGSWVVIDSSINPRHMEMYADRESRGSV 200

|:.||...|||.||||...|||||:|||:||.||..::|||||..:||:|

EMBOSS_001 148 VENLRTYRQPVFVYIPMMGELRGGAWVVVDSQINSDYVEMYADETARGNV 197

EMBOSS_001 201 LEPEGTVEIKFRRKDLVKTMRRVDPVYIHLAERLGTPELST--AERKELE 248

||||||:|||||.|:|::.|.|:|...|.|..:|...:.|. |..:.|:

EMBOSS_001 198 LEPEGTIEIKFRTKELLECMGRLDQKLISLKAKLQDAKQSEAYANIELLQ 247

EMBOSS_001 249 NKLKEREEFLIPIYHQVAVQFADLHDTPGRMQEKGVISDILDWKTSRTFF 298

.::|.||:.|:|:|.|:|.:||:||||..||..||||..:::|..||:||

EMBOSS_001 248 QQIKAREKQLLPVYIQIATKFAELHDTSMRMAAKGVIKSVVEWSGSRSFF 297

EMBOSS_001 299 YWRLRRLLLEDLVKKKIHN 317

|.:|.|.:.|..:.|.:..

EMBOSS_001 298 YKKLNRRIAESSLVKNVRE 316

#---------------------------------------

#---------------------------------------

**Protoporphyrinogen IX oxidase - PPOX (*Nicotiana tabacum* and *Homo sapiens)***

***UNIPROT***

1. Human (Homo sapiens): PPOX_HUMAN (P50336)

- <https://www.uniprot.org/uniprotkb/P50336/entry>
- Region: 1-477 (Protoporphyrinogen oxidase)

**Sequence:**MGRTVVVLGGGISGLAASYHLSRAPCPPKVVLVESSERLGGWIRSVRGPNGAIFELGPRGIRPAGALGARTLLLVSELGLDSEVLPVRGDHPAAQNRFLYVGGALHALPTGLRGLLRPSPPFSKPLFWAGLRELTKPRGKEPDETVHSFAQRRLGPEVASLAMDSLCRGVFAGNSRELSIRSCFPSLFQAEQTHRSILLGLLLGAGRTPQPDSALIRQALAERWSQWSLRGGLEMLPQALETHLTSRGVSVLRGQPVCGLSLQAEGRWKVSLRDSSLEADHVISAIPASVLSELLPAEAAPLARALSAITAVSVAVVNLQYQGAHLPVQGFGHLVPSSEDPGVLGIVYDSVAFPEQDGSPPGLRVTVMLGGSWLQTLEASGCVLSQELFQQRAQEAAATQLGLKEMPSHCLVHLHKNCIPQYTLGHWQKLESARQFLTAHRLPLTLAGASYEGVAVNDCIESGRQAAVSVLGTEPNS

2. Plant (Nicotiana tabacum): PPOM_TOBAC (O24164)

- <https://www.uniprot.org/uniprotkb/O24164/entry>
- Region: 1-504 (Protoporphyrinogen oxidase, mitochondrial)

**Sequence:**MAPSAGEDKHSSAKRVAVIGAGVSGLAAAYKLKIHGLNVTVFEAEGKAGGKLRSVSQDGLIWDEGANTMTESEGDVTFLIDSLGLREKQQFPLSQNKRYIARNGTPVLLPSNPIDLIKSNFLSTGSKLQMLLEPILWKNKKLSQVSDSHESVSGFFQRHFGKEVVDYLIDPFVAGTCGGDPDSLSMHHSFPELWNLEKRFGSVILGAIRSKLSPKNEKKQGPPKTSANKKRQRGSFSFLGGMQTLTDAICKDLREDELRLNSRVLELSCSCTEDSAIDSWSIISASPHKRQSEEESFDAVIMTAPLCDVKSMKIAKRGNPFLLNFIPEVDYVPLSVVITTFKRENVKYPLEGFGVLVPSKEQQHGLKTLGTLFSSMMFPDRAPNNVYLYTTFVGGSRNRELAKASRTELKEIVTSDLKQLLGAEGEPTYVNHLYWSKAFPLYGHNYDSVLDAIDKMEKNLPGLFYAGNHRGGLSVGKALSSGCNAADLVISYLESVSTDSKRHC

emboss_needle-I20251215-231032-0110-45760878-p1m

########################################

# Program: needle

# Rundate: Mon 15 Dec 2025 23:10:35

# Commandline: needle

# -auto

# -stdout

# -asequence emboss_needle-I20251215-231032-0110-45760878-p1m.asequence

# -bsequence emboss_needle-I20251215-231032-0110-45760878-p1m.bsequence

# -datafile EBLOSUM62

# -gapopen 10.0

# -gapextend 0.5

# -endopen 10.0

# -endextend 0.5

# -aformat3 pair

# -sprotein1

# -sprotein2

# Align_format: pair

# Report_file: stdout

########################################

#=======================================

#

# Aligned_sequences: 2

# 1: EMBOSS_001

# 2: EMBOSS_001

# Matrix: EBLOSUM62

# Gap_penalty: 10.0

# Extend_penalty: 0.5

#

# Length: 563

# Identity: 138/563 (24.5%)

# Similarity: 218/563 (38.7%)

# Gaps: 145/563 (25.8%)

# Score: 278.5

#

#

#=======================================

EMBOSS_001 1 -----------MGRTVVVLGGGISGLAASYHLSRAPCPPKVVLVESSERL 39

..:.|.|:|.|:|||||:|.|..... .|.:.|:..:.

EMBOSS_001 1 MAPSAGEDKHSSAKRVAVIGAGVSGLAAAYKLKIHGL--NVTVFEAEGKA 48

EMBOSS_001 40 GGWIRSVRGPNGAIFELGPRGIRPAGALGARTL--------LLVSELGLD 81

||.:||| ..:|.|:: .||.|: .|:..|||.

EMBOSS_001 49 GGKLRSV-SQDGLIWD-----------EGANTMTESEGDVTFLIDSLGLR 86

EMBOSS_001 82 SEVLPVRGDHPAAQN-RFLYVGGALHALPTGLRGLLRPS--------PPF 122

. :...|.:|| |::...|....||:....|::.: ...

EMBOSS_001 87 E-----KQQFPLSQNKRYIARNGTPVLLPSNPIDLIKSNFLSTGSKLQML 131

EMBOSS_001 123 SKPLFWAGLRELTKPRGKEPDETVHSFAQRRLGPEVASLAMDSLCRGVFA 172

.:|:.|.. ::|::. .:..|:|..|.||..|.||....:|....|...

EMBOSS_001 132 LEPILWKN-KKLSQV--SDSHESVSGFFQRHFGKEVVDYLIDPFVAGTCG 178

EMBOSS_001 173 GNSRELSIRSCFPSLFQAEQTHRSILLGLLLG-------AGRTPQPDSAL 215

|:...||:...||.|:..|:...|::||.:.. ..:.|...||.

EMBOSS_001 179 GDPDSLSMHHSFPELWNLEKRFGSVILGAIRSKLSPKNEKKQGPPKTSAN 228

EMBOSS_001 216 IRQALAERWSQWSLRGGLEMLPQAL-------ETHLTSRGVSVLRGQPVC 258

.:: :|.| :|..||::.|..|: |..|.|| ||.....|

EMBOSS_001 229 KKR---QRGS-FSFLGGMQTLTDAICKDLREDELRLNSR---VLELSCSC 271

EMBOSS_001 259 GLSLQAEGRWKVSLRDSSLEADHVISAIPASVLSELLPAEA----APLAR 304

..||::::..:|||.|....||....:| |||..

EMBOSS_001 272 -------------TEDSAIDSWSIISASPHKRQSEEESFDAVIMTAPLCD 308

EMBOSS_001 305 ALSAITA------------------VSVAVVNLQYQGAHLPVQGFGHLVP 336

..|...| :||.:...:.:....|::|||.|||

EMBOSS_001 309 VKSMKIAKRGNPFLLNFIPEVDYVPLSVVITTFKRENVKYPLEGFGVLVP 358

EMBOSS_001 337 SSEDP---GVLGIVYDSVAFPEQDGSPPGLRV-TVMLGGSWLQTLEASGC 382

|.|.. ..||.::.|:.|| |.:|..:.: |..:|||..:.|..:..

EMBOSS_001 359 SKEQQHGLKTLGTLFSSMMFP--DRAPNNVYLYTTFVGGSRNRELAKASR 406

EMBOSS_001 383 VLSQELFQQRAQEAAATQLGLKEMPSHCLVHLH-KNCIPQYTLGH----- 426

...:|:.....::. ||.:..|:: :.||: ....|.| ||

EMBOSS_001 407 TELKEIVTSDLKQL----LGAEGEPTY-VNHLYWSKAFPLY--GHNYDSV 449

EMBOSS_001 427 ---WQKLESARQFLTAHRLP-LTLAGASYEGVAVNDCIESGRQAAVSVLG 472

..|:|. .|| |..||....|::|...:.||..||..|:.

EMBOSS_001 450 LDAIDKMEK--------NLPGLFYAGNHRGGLSVGKALSSGCNAADLVIS 491

EMBOSS_001 473 -TEPNS------- 477

.|..|

EMBOSS_001 492 YLESVSTDSKRHC 504

#---------------------------------------

#---------------------------------------

**Protoporphyrinogen IX oxidase - PPOX (*Nicotiana tabacum* and *Homo sapiens)***

1. Human (Homo sapiens): PPOX_HUMAN (P50336)

- <https://www.uniprot.org/uniprotkb/P50336/entry>
- Region: 1-477 (Protoporphyrinogen oxidase)

**Sequence:**MGRTVVVLGGGISGLAASYHLSRAPCPPKVVLVESSERLGGWIRSVRGPNGAIFELGPRGIRPAGALGARTLLLVSELGLDSEVLPVRGDHPAAQNRFLYVGGALHALPTGLRGLLRPSPPFSKPLFWAGLRELTKPRGKEPDETVHSFAQRRLGPEVASLAMDSLCRGVFAGNSRELSIRSCFPSLFQAEQTHRSILLGLLLGAGRTPQPDSALIRQALAERWSQWSLRGGLEMLPQALETHLTSRGVSVLRGQPVCGLSLQAEGRWKVSLRDSSLEADHVISAIPASVLSELLPAEAAPLARALSAITAVSVAVVNLQYQGAHLPVQGFGHLVPSSEDPGVLGIVYDSVAFPEQDGSPPGLRVTVMLGGSWLQTLEASGCVLSQELFQQRAQEAAATQLGLKEMPSHCLVHLHKNCIPQYTLGHWQKLESARQFLTAHRLPLTLAGASYEGVAVNDCIESGRQAAVSVLGTEPNS

3. Plant (Nicotiana tabacum): PPOC_ARATH (P55826)

- <https://www.uniprot.org/uniprotkb/P55826/entry>

Region: 35-537 (Protoporphyrinogen oxidase 1, chloroplastic)

**Sequence:**SVAGGPTVGSSKIEGGGGTTITTDCVIVGGGISGLCIAQALATKHPDAAPNLIVTEAKDRVGGNIITREENGFLWEEGPNSFQPSDPMLTMVVDSGLKDDLVLGDPTAPRFVLWNGKLRPVPSKLTDLPFFDLMSIGGKIRAGFGALGIRPSPPGREESVEEFVRRNLGDEVFERLIEPFCSGVYAGDPSKLSMKAAFGKVWKLEQNGGSIIGGTFKAIQERKNAPKAERDPRLPKPQGQTVGSFRKGLRMLPEAISARLGSKVKLSWKLSGITKLESGGYNLTYETPDGLVSVQSKSVVMTVPSHVASGLLRPLSESAANALSKLYYPPVAAVSISYPKEAIRTECLIDGELKGFGQLHPRTQGVETLGTIYSSSLFPNRAPPGRILLLNYIGGSTNTGILSKSEGELVEAVDRDLRKMLIKPNSTDPLKLGVRVWPQAIPQFLVGHFDILDTAKSSLTSSGYEGLFLGGNYVAGVALGRCVEGAYETAIEVNNFMSRYAYK

***EMBL-EBI***

- <https://www.ebi.ac.uk/jdispatcher/psa/emboss_needle>

**Results for Job ID:** emboss_needle-I20251215-221318-0945-51046600-p1m

########################################

# Program: needle

# Rundate: Mon 15 Dec 2025 22:13:22

# Commandline: needle

# -auto

# -stdout

# -asequence emboss_needle-I20251215-221318-0945-51046600-p1m.asequence

# -bsequence emboss_needle-I20251215-221318-0945-51046600-p1m.bsequence

# -datafile EBLOSUM62

# -gapopen 10.0

# -gapextend 0.5

# -endopen 10.0

# -endextend 0.5

# -aformat3 pair

# -sprotein1

# -sprotein2

# Align_format: pair

# Report_file: stdout

########################################

#=======================================

#

# Aligned_sequences: 2

# 1: EMBOSS_001

# 2: EMBOSS_001

# Matrix: EBLOSUM62

# Gap_penalty: 10.0

# Extend_penalty: 0.5

#

# Length: 562

# Identity: 143/562 (25.4%)

# Similarity: 234/562 (41.6%)

# Gaps: 144/562 (25.6%)

# Score: 421.5

#

#

#=======================================

EMBOSS_001 1 ----------------MGRTV----VVLGGGISGLAASYHLS--RAPCPP 28

.|.|: |::|||||||..:..|: .....|

EMBOSS_001 1 SVAGGPTVGSSKIEGGGGTTITTDCVIVGGGISGLCIAQALATKHPDAAP 50

EMBOSS_001 29 KVVLVESSERLGGWIRSVRGPNGAIFELGPRGIRPAGALGARTLLLVSEL 78

.:::.|:.:|:||.| ..|..||.::|.||...:|:..: |.:|.:.

EMBOSS_001 51 NLIVTEAKDRVGGNI-ITREENGFLWEEGPNSFQPSDPM----LTMVVDS 95

EMBOSS_001 79 GLDSEVLPVRGDHPAAQNRFLYVGGALHALPTGLRGL------------- 115

||..:: |.|| |.|. ||:...|.|..:|:.|..|

EMBOSS_001 96 GLKDDL--VLGD-PTAP-RFVLWNGKLRPVPSKLTDLPFFDLMSIGGKIR 141

EMBOSS_001 116 -------LRPSPPFSKPLFWAGLRELTKPRGKEPDETVHSFAQRRLGPEV 158

:||||| |:| |:|..|.:|.||.||

EMBOSS_001 142 AGFGALGIRPSPP-----------------GRE--ESVEEFVRRNLGDEV 172

EMBOSS_001 159 ASLAMDSLCRGVFAGNSRELSIRSCFPSLFQAEQTHRSILLGLLLGA--- 205

....::..|.||:||:..:||:::.|..:::.||...||:.|.....

EMBOSS_001 173 FERLIEPFCSGVYAGDPSKLSMKAAFGKVWKLEQNGGSIIGGTFKAIQER 222

EMBOSS_001 206 ---------GRTPQPDSALIRQALAERWSQWSLRGGLEMLPQALETHLTS 246

.|.|:|....: .|.|.||.|||:|:...|

EMBOSS_001 223 KNAPKAERDPRLPKPQGQTV----------GSFRKGLRMLPEAISARL-- 260

EMBOSS_001 247 RGVSVLRGQPVCGLSLQAEGRWKVSLRDS----SLEADHVISAIPASVLS 292

|..|.....:.|::....|.:.::.... |:::..|:..:|:.|.|

EMBOSS_001 261 -GSKVKLSWKLSGITKLESGGYNLTYETPDGLVSVQSKSVVMTVPSHVAS 309

EMBOSS_001 293 ELLPAEAAPLARALSAITAVSVAVVNLQYQGAHL--------PVQGFGHL 334

.||...:...|.|||.:....||.|::.|....: .::|||.|

EMBOSS_001 310 GLLRPLSESAANALSKLYYPPVAAVSISYPKEAIRTECLIDGELKGFGQL 359

EMBOSS_001 335 VPSSEDPGVLGIVYDSVAFPEQDGSPPGLRVTVM--LGGSWLQTLEASGC 382

.|.::....||.:|.|..||.: :||| |:.:: :||| .:..

EMBOSS_001 360 HPRTQGVETLGTIYSSSLFPNR--APPG-RILLLNYIGGS------TNTG 400

EMBOSS_001 383 VLSQELFQQRAQEAAATQLGLKEM--------PSHCLVHLHKNCIPQYTL 424

:|| :...:...|....|::| |....|.:....|||:.:

EMBOSS_001 401 ILS----KSEGELVEAVDRDLRKMLIKPNSTDPLKLGVRVWPQAIPQFLV 446

EMBOSS_001 425 GHWQKLESARQFLTAHRLPLTLAGASY-EGVAVNDCIESGRQAAVSVLGT 473

||:..|::|:..||:........|.:| .|||:..|:|...:.|:.|

EMBOSS_001 447 GHFDILDTAKSSLTSSGYEGLFLGGNYVAGVALGRCVEGAYETAIEV--- 493

EMBOSS_001 474 EPNS-------- 477

|:

EMBOSS_001 494 --NNFMSRYAYK 503

#---------------------------------------

#---------------------------------------
