# Supplementary material for: Shared Plant-human Biology: Herbicide Effects and New Biomarkers Perspectives
Source: Curr Environ Health Rep. 2026 Apr 2;13(1):16. doi: 10.1007/s40572-026-00523-z (PMC13046696; doi:10.1007/s40572-026-00523-z)
Supplement: Supplementary file 2 — Supplementary Material 2 (PDF 309 KB) [file 40572_2026_523_MOESM2_ESM.pdf]

Aline Espindola <esp.aline@iesc.ufrj.br>

Query on Your Paper EHP17631 - [EMID:dbb9570192c81e51]

4 mensagens

Kim A Gaetz <em@editorialmanager.com> 19 de maio de 2025 às 17:34
Responder a: Kim A Gaetz <kim.gaetz@nih.gov>
Para: Aline de Souza Espindola <esp.aline@iesc.ufrj.br>

MS Number: EHP17631
Title: Shared plant-human biology: herbicide effects and new biomarkers perspectives
Authors: Aline de Souza Espindola; Christine G. Parks; Josino Costa Moreira

Dear Aline de Souza Espindola,

As you know from our previous email exchange, because we could no longer guarantee that we will be able to complete our usual evaluation and processing of your paper, we made the difficult decision to stop further consideration of your paper.

Please know that this decision was not taken lightly, and that the withdrawal of your submission is unrelated to the quality of your manuscript. Indeed, your paper was considered of high interest and among the few manuscripts selected for peer review and we enjoyed reading and considering your work for publication in EHP.

As mentioned in our email exchange on May 9, given the similar scope of their journal, the editors of Current Environmental Health Reports (https://link.springer.com/journal/40572) have assured us that they would accept a transfer of your seminar manuscript to their journal.
If you are interested transferring your manuscript to their journal, please let us know as soon as possible, and we can take care of transferring your manuscript and review files to them to expedite your manuscript's consideration.

The reviewer comments for your submission are included at the bottom of this letter. You may wish to include copies of the reviews if you choose to submit your submission elsewhere. We are happy to confirm the veracity of review comments to other journals and to confirm that your paper was under review and not rejected by EHP; please request confirmation via ehpsubmissions@niehs.nih.gov. This may expedite your manuscript's consideration at another journal. You can submit your paper to another journal immediately.

We appreciate your consideration of EHP and are very sorry for the delays that this might cause in publication of your important work. Please check the EHP Publishing website for updated information (https://ehp.niehs.nih.gov/)

Sincerely,

Windy Boyd
Senior Science Editor

Joel Kaufman
Editor-in-Chief
Environmental Health Perspectives

Editor comments:
This one is much improved but needs more work.

Reviewer comments:

Reviewer 1:
I would like to commend the authors for addressing the concerns from their initial submission inquiry. The authors do a better job of connecting the plant and human aspects; however, there is a limited consideration for the following points raised in the previous review:
1. The authors do not overtly state the similarities between the plant and human targets highlighted in the review. For example, how conserved are the toxicant interacting sites and the physiological responses to the herbicide?
2. The authors do not robustly consider non-enzyme targets that may affect enzyme functions. A prime example of this is the consideration that AMPK, an often-activated protein kinase to toxicants, as a modulator of ACC activity, an enzyme highlighted by the authors. These indirect effects are equally important to the overall toxicity as the direct targeting. Because of the broad conservation of signaling components, like AMPK, this may need to be addressed by the authors. Other than those points, the article is well written and highlights important knowledge in the field.

Reviewer 2:
This revised manuscript summarizes current knowledge regarding plant molecular pathways and enzymes that are targeted by herbicides. Given plants and humans share some of these pathways, the authors proceed to highlight some of these conserved pathways, which could underlie sublethal but toxic effects of herbicide exposure in humans. These mechanisms could represent opportunities for biomarker development to detect herbicide exposure in humans prior to development of pathophysiology. Finally, the authors described other mechanisms that could potentially underlie herbicide-induced effects on human physiology. The authors were receptive to feedback from the prior review and made adequate improvements to the manuscript. I have some minor comments that could further improve the manuscript. Overall, this should be a valuable contribution to an important topic in environmental toxicology.

Comments: 1. Line 80: are the authors referring to "early damage caused by herbicides" in humans? They should be more specific. 2. Table 1 is a valuable addition to this work. I suggest adding two more columns with the following information: (1) primary molecular target in plants; (2) presence of conserved molecular target in humans? 3. Line 132: "Taking" should be "Taken". 4. Line 204: change to "therefore represent targets of herbicides". 5. Enzymes names in subtitles, e.g. HPPD in 2.2.1, should be written in full together with short form.

Reviewer 3:
This is an interesting seminar style paper that explores how shared biology across plants and animals may influence response to herbicides. There are many strengths: nice job of laying out the specific herbicide chemical classes. I found prior reviewer comments to have been thoughtfully and thoroughly addressed.
Suggestions:
1. For each use class in section 2.1, estimate annual use and expected exposures where possible.
2. Section 2.2 is very heavy on acronyms.

In compliance with data protection regulations, you may request that we remove your personal registration details at any time. (Use the following URL: https://www.editorialmanager.com/ehp/login.asp?a=r). Please contact the publication office if you have any questions.

Aline Espindola <esp.aline@iesc.ufrj.br> 20 de maio de 2025 às 16:23
Para: Kim A Gaetz <kim.gaetz@nih.gov>

Dear Kim Gaetz,

Thank you for your message and the consideration given to my manuscript.

I confirm my agreement to the transfer of my seminar manuscript and the review files to *Current Environmental Health Reports*, as suggested.

I understand that your team will handle the transfer process, and I sincerely appreciate your support in facilitating this transition. Please let me know if any further action is needed on my part.

Best regards,
Aline de Souza Espindola

[Texto das mensagens anteriores oculto]

--
Aline de Souza Espíndola, MPH, PhD
Professor of Occupational and Environmental Health
Public Health Institute
Federal University of Rio de Janeiro
ORCID: https://orcid.org/0000-0002-5498-3992

Gaetz, Kim (NIH/NIEHS) [E] <kim.gaetz@nih.gov> 20 de maio de 2025 às 17:36
Para: Aline Espindola <esp.aline@iesc.ufrj.br>
Cc: "em.ehp-04ea08a8e73bfb6b@editorialmanager.com" <em.ehp-04ea08a8e73bfb6b@editorialmanager.com>, Environmental Health Perspectives <em@editorialmanager.com>

Hello Aline,

Thank you for confirming that you would like to transfer the seminar paper to *Current Environmental Health Reports*.

We will let you know if we need anything further from you to proceed.

Best wishes,

Kim Gaetz

[Texto das mensagens anteriores oculto]

CAUTION: This email originated from outside of the organization. Do not click links or open attachments unless you recognize the sender and are confident the content is safe.

Gaetz, Kim (NIH/NIEHS) [E] <kim.gaetz@nih.gov> 21 de maio de 2025 às 15:25
Para: Aline Espindola <esp.aline@iesc.ufrj.br>
Cc: EM DropBox <em.ehp-04ea08a8e73bfb6b@editorialmanager.com>

Aline,

I initiated the transfer with CEHR, so you should be hearing from them soon. Cody Adams is their managing editor.

Best wishes,

Kim

From: Aline Espindola <esp.aline@iesc.ufrj.br>
Sent: Tuesday, May 20, 2025 3:24 PM
To: Gaetz, Kim (NIH/NIEHS) [E] <kim.gaetz@nih.gov>
Subject: [EXTERNAL] Re: Query on Your Paper EHP17631 - [EMID:dbb9570192c81e51]

Dear Kim Gaetz,

[Texto das mensagens anteriores oculto]
[Texto das mensagens anteriores oculto]

CAUTION: This email originated from outside of the organization. Do not click links or open attachments unless you recognize the sender and are confident the content is safe.
